# Interconversions between RNS Revealed by Transient Voltammetry with Porphyrin‐Modified Carbon Nanopipettes in Single Living Cells

**DOI:** 10.1002/advs.202512865

**Published:** 2025-10-06

**Authors:** Hongli Cao, Dehui Yu, Yingjie Zhao, Xiaoyue Shen, Rujia Liu, Dengchao Wang

**Affiliations:** ^1^ School of Chemical Sciences University of Chinese Academy of Sciences Beijing 100049 P. R. China; ^2^ Binzhou Institute of Technology University of Chinese Academy of Sciences Weiqiao‐UCAS Science and Technology Park Binzhou Shandong 256606 P. R. China

**Keywords:** conductive nanopipette, reactive nitrogen species, single cell electrochemistry, transient voltammetry

## Abstract

Reactive nitrogen species (RNS) play crucial roles in cellular signaling, but their quantification is very challenging due to low‐abundant nature and complex conversions with other reactive molecules in the cells. The well‐established amperometry methods have been widely used to measure the RNS inside the living cells, while the nanometer‐sized tip is limited by the steady‐state studies. In this paper, conductive nanopipettes (CNPs) with porphyrin complexes are functionalized, which allow direct transient voltammetry tests to differentiate and quantify the RNS based on the peak potential and current. It is shown that the oxidation of NO_2_^‒^ can produce a nitrogen dioxide (NO_2_) intermediate, and a CEC (chemical‐electrochemical‐chemical) mechanism is proposed. Moreover, this platform with transient voltammetry enables real‐time quantification of cellular RNS and reveals the dynamic interconversions within RNS, providing valuable insights into their roles in cellular processes.

## Graphical abstract

By using porphyrin modified conductive nanopipettes, transient voltammetry can be directly applied to reveal the dynamic and complex electrochemical reactions between the reactive nitrogen species inside the single living cells.

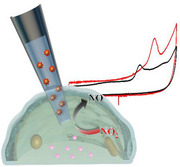

## 1 Introduction

Reactive nitrogen species (RNS), including nitric oxide (NO) and its oxidized derivatives (i.e., NO_2_, NO_2_^−^, and ONOO^−^), are essential in cellular signaling and oxidative stress.^[^ ^1^, ^2^, ^3^ ^]^ They support immune defense and vasodilation processes, and cause cellular damage and cancer and neurodegeneration diseases once they are dysregulated.^[^ ^4^, ^5^, ^6^, ^7^ ^]^ Understanding and maintaining intracellular RNS levels is thus crucial for both fundamental biological research and practical therapeutic applications. However, the dynamic, reactive, and low‐abundant nature of RNS in living cells poses significant challenges for their accurate detection and quantification.^[^ ^8^ ^]^ For example, nitric oxide (NO) is particularly difficult to monitor due to its short half‐life (< 10 s), low concentration (sub‐µm), and the ease with which it reacts with reactive oxygen species, and interconverts with nitrites (NO_2_^−^) and nitrogen dioxide (NO_2_).^[^ ^9^, ^10^ ^]^

A variety of techniques have been used to detect the RNS species, including fluorescence,^[^ ^11^, ^12^, ^13^, ^14^, ^15^ ^]^ luminescent,^[^ ^16^ ^]^ colorimetric,^[^ ^17^ ^]^ electron paramagnetic resonance,^[^ ^18^ ^]^ and high‐performance liquid chromatography.^[^ ^11^, ^19^, ^20^ ^]^ Among the various techniques, ultramicroelectrodes and nanoelectrodes based electrochemical methods have shown the advantages of easy operation, in situ and non‐invasive detection, and high spatial and temporal resolution at the single‐cell level.^[^ ^21^, ^22^, ^23^, ^24^, ^25^, ^26^, ^27^, ^28^, ^29^ ^]^ However, the nanoelectrodes are typically limited to steady‐state electrochemical studies, which have the disadvantages of having only a single characteristic time (r_0_^2^/*D*, r_0_ is the electrode radius, *D* is the diffusion coefficient).^[^ ^30^ ^]^ The transient characteristics of the reaction of interests, such as the interconversion between RNS or coupled reactions in solutions, cannot be easily revealed by the resulting cyclic voltammogram at nanoelectrodes, unless the potential scan rate goes to extreme high (i.e., > 10^7^ V s^−1^).^[^ ^31^ ^]^

**Scheme 1:**
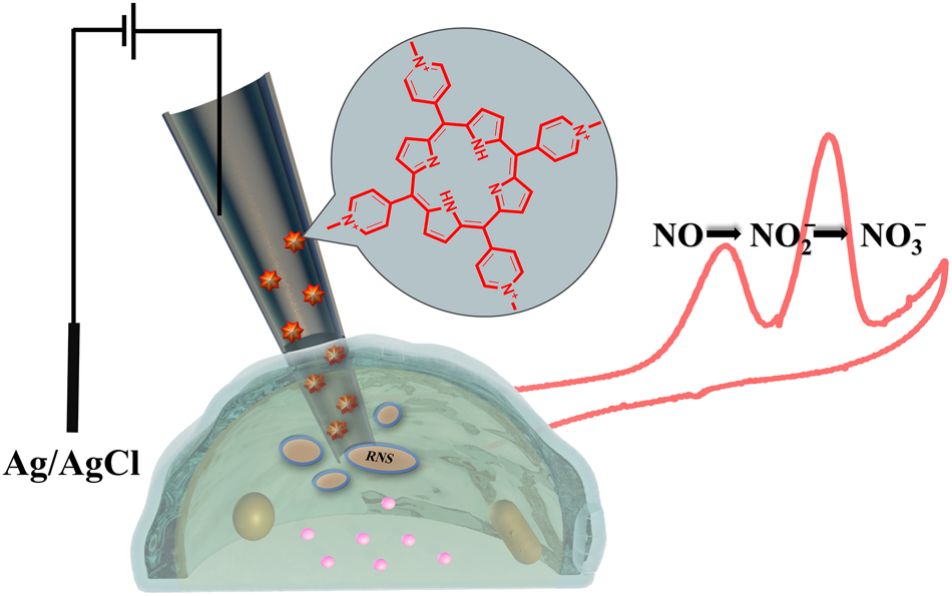
Schematic illustration of cyclic voltammetry studies of intracellular RNS with porphyrin‐modified carbon nanopipettes.

The conductive nanopipettes, with a large electroactive interface inside the nanopipette, could offer a new opportunity for transient voltammetry studies in single living cells. The nano‐sized tip would enable the non‐invasive insertion inside the cell, and the large inside cavity would lead to thin‐layer electrochemistry with tunable timescale by varying scan rate (*v*). Transient features or coupled chemical reactions can then be resolved by optimizing the potential scan rate.^[^ ^32^, ^33^ ^]^ Moreover, the large interior surface could also be modified toward specific analytes tests at nanoelectrodes, to reveal the complex reactions in single living cells (**Scheme** **1**).^[^ ^34^, ^35^, ^36^ ^]^

## 2 Results and Discussion

**Figure 1:**
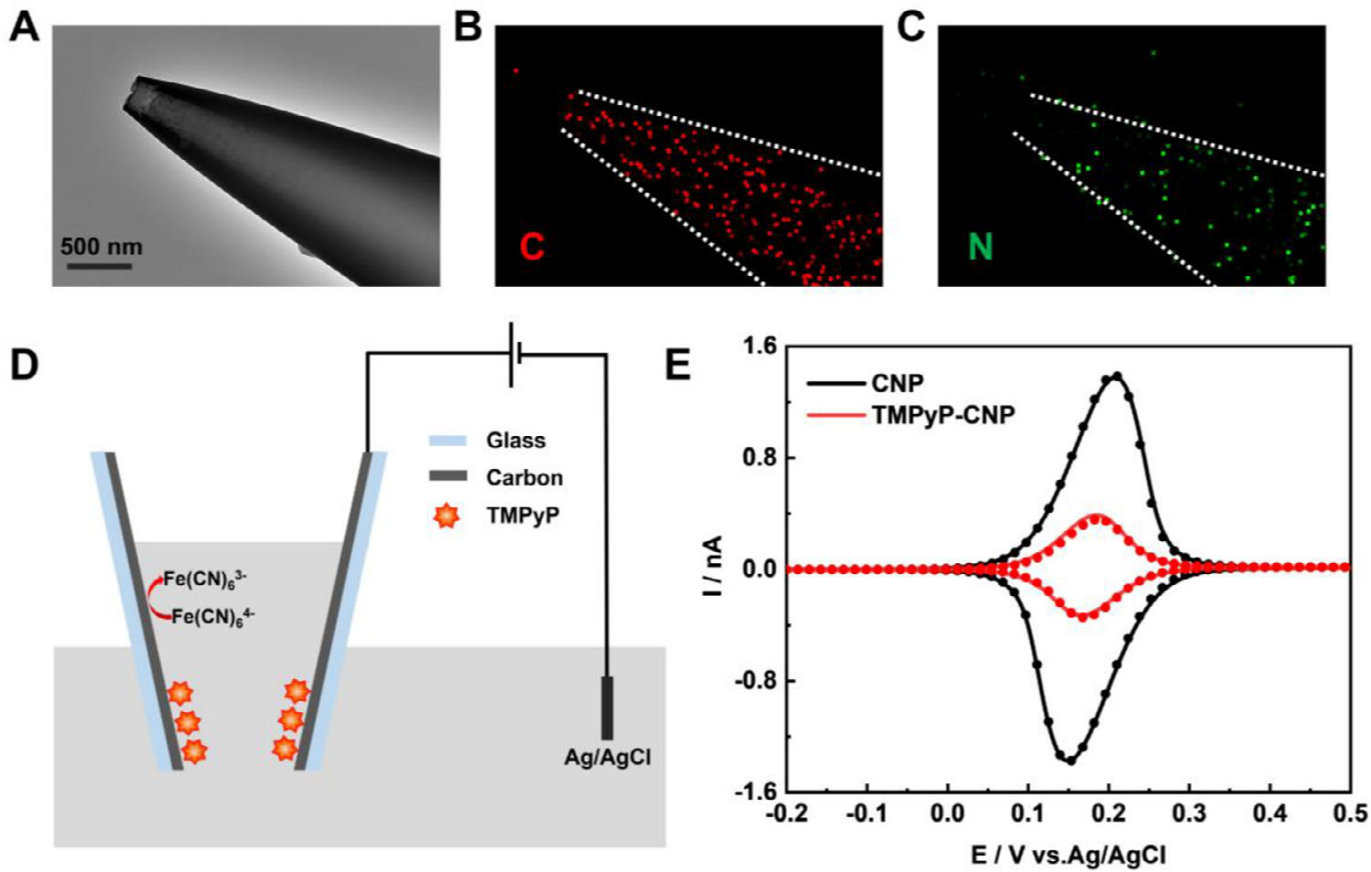
A) A representative TEM image of the TMPyP‐CNP. Corresponding EDS maps highlighting the elements of B) C and C) N. D) Electrochemical characterization of the TMPyP‐CNP. E) Experimental (curves) and simulated (dots) CVs of the CNP and TMPyP‐CNP in 1 mm K_4_Fe(CN)_6_ and 0.1 m KCl, *v* = 0.1 V s^−1^.

The carbon nanopipette (CNP) was fabricated from quartz nanopipettes with a chemical vapor deposition method described previously.^[^ ^37^ ^]^ Then, by immersing in 5,10,15,20‐tetra(4‐pyridyl)‐21H,23H‐porphine (TMPyP) solution for ≈5 min, the CNP was modified with TMPyP at its interior surface by electrostatic adsorption, to form the TMPyP‐CNP. **Figure** **1**A shows a representative transmission electron microscopy (TEM) image of TMPyP‐CNP, with a retained open pipette geometry from CNP (Figure S1A, Supporting Information). The corresponding EDS maps in panels B and C, reveal a homogeneous distribution of carbon and nitrogen elements inside the nanopipettes, confirming the successful modification of porphyrin.

Next, we also use electrochemical tests to evaluate the TMPyP modification processes. By applying potential at the carbon with respect to Ag/AgCl (Figure 1D), cyclic voltammetry (CV) was performed in 0.1 m KCl and 1 mm K_4_Fe(CN)_6_ at 0.1 V s^−1^. Shown in Figure 1E, typical thin‐layer electrochemical responses with symmetrical peaks were resulted from both CNP and TMPyP‐CNP, originating from [Fe(CN)_6_]^3−/4−^ oxidation/reduction inside the pipette. The smaller peak currents from the TMPyP‐CNP indicate the modification of TMPyP, as its conductivity is lower than that of carbon.^[^ ^38^ ^]^ The peak currents continue to decrease as the modification time increases (Figure S2, Supporting Information). At ≈20 min, nearly all the carbon surface are covered, as almost zero current was obtained. The peak currents could then be fitted to roughly evaluate the modification efficiency by conducting a numerical simulation. Based on a simple diffusional model, ≈40% of the carbon surface are covered by TMPyP.

**Figure 2:**
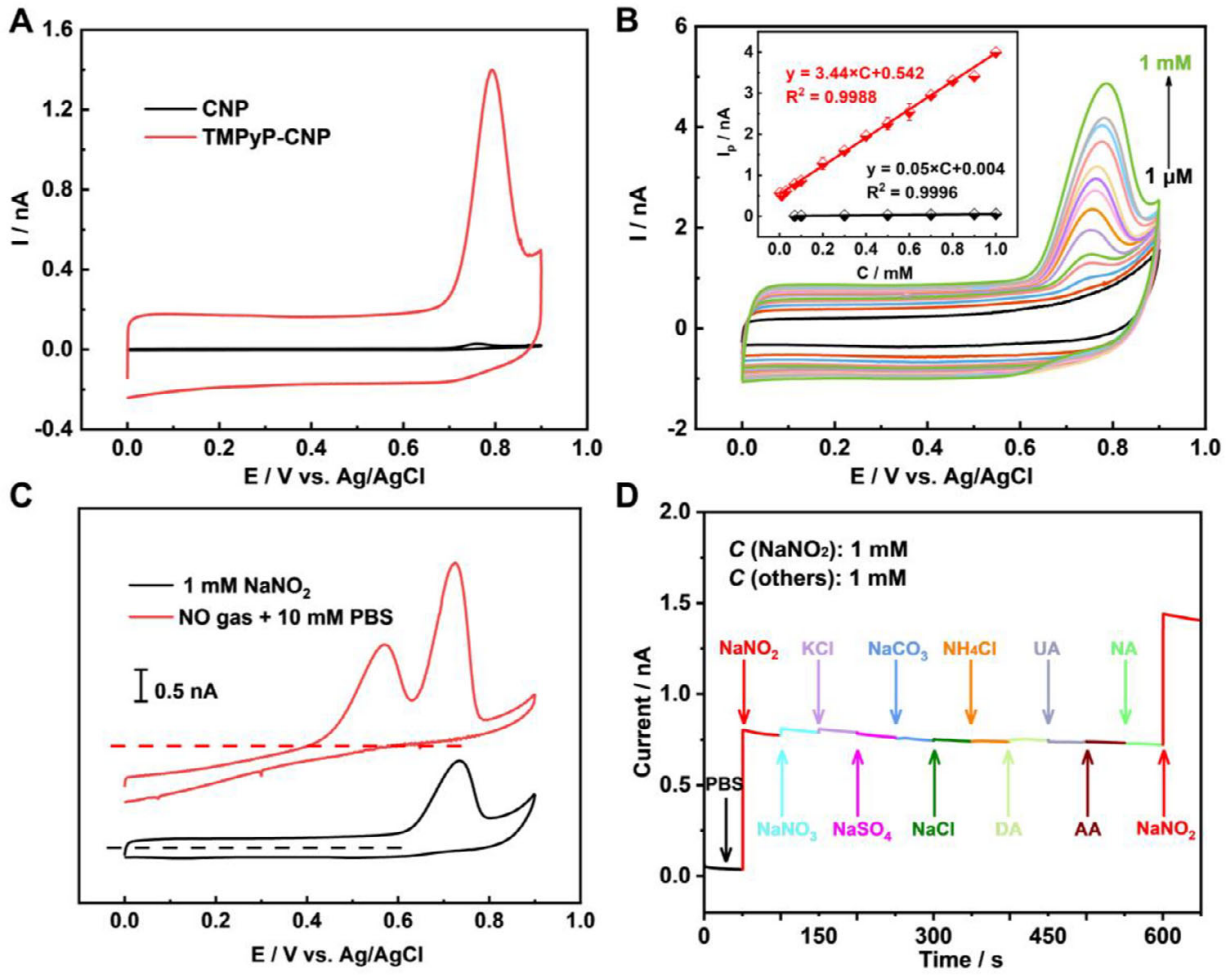
CVs of different tips in 10 mm PBS: A) The CNP and TMPyP‐CNP in 1 mm NaNO_2_, B) TMPyP‐CNP at different NaNO_2_ concentrations. Inset: calibration plot for the CNP (black line) and TMPyP‐CNP (red line). C) The TMPyP‐CNP for 0.1% NO/N_2_ in PBS and 1 mm NaNO_2_. D) Selectivity study of the TMPyP‐CNP at 0.75 V.

With the fabricated TMPyP‐CNP, the sensing performance toward nitrogen species was then studied. In 1 mm NaNO_2_ and 10 mm PBS, the TMPyP‐CNP clearly displays a much larger oxidation current than that of CNPs ≈ +0.76 V (**Figure** **2**A). The increasing peak current at higher NaNO_2_ concentrations (Figure 2B; Figure S3, Supporting Information) confirms the origin of the peak from nitrite oxidation. Linear calibration curves of the peak currents at various concentrations were then obtained and shown as the inset in Figure 2B. Based on the fitted slopes, the sensitivity of TMPyP‐CNP (3.44 nA mm ^−1^) is ≈70 times larger than that of CNP (0.05 nA mm ^−1^). Such an increase would be from the positively‐charged TMPyP that attracts NO_2_^−^ ions^[^ ^39^ ^]^ and catalyzes the nitrite oxidation.^[^ ^40^ ^]^ Overall, the TMPyP‐CNP displays a wide detection range and low detection limit when compared to previous reported sensors (Table S1, Supporting Information).

Besides NO_2_^−^, NO is also an important RNS that has complicated reaction pathways.^[^ ^41^ ^]^ Excitingly, the TMPyP‐CNP also exhibits excellent sensing performance toward NO, as demonstrated by two strong and well‐separated oxidation peaks (Figure 2C). The first peak at +0.56 V indicates the oxidation of NO into NO_2_^−^, while the second oxidation peak at +0.76 V closely matches the oxidation peak of NO_2_^−^ (black line), suggesting the further oxidation of NO_2_^−^ into NO_3_^−^. As a result, the TMPyP‐CNPs can effectively distinguish NO and NO_2_^−^ species at relative low potentials in CVs. Moreover, the fabricated electrode also displays good stability, with only ≈4.2% signal degradation after 50 consecutive CV scans (Figure S5, Supporting Information), as well as excellent selectivity (Figure 2D), as the presence of NaNO_3_, NaCl, dopamine (DA) and uric acid (UA), do not affect the current signal of NaNO_2_. Most importantly, the TMPyP‐CNP exhibits no response to H_2_O_2_ (Figure S6, Supporting Information), which would largely eliminate the interference of H_2_O_2_ in single cell analysis.

**Figure 3:**
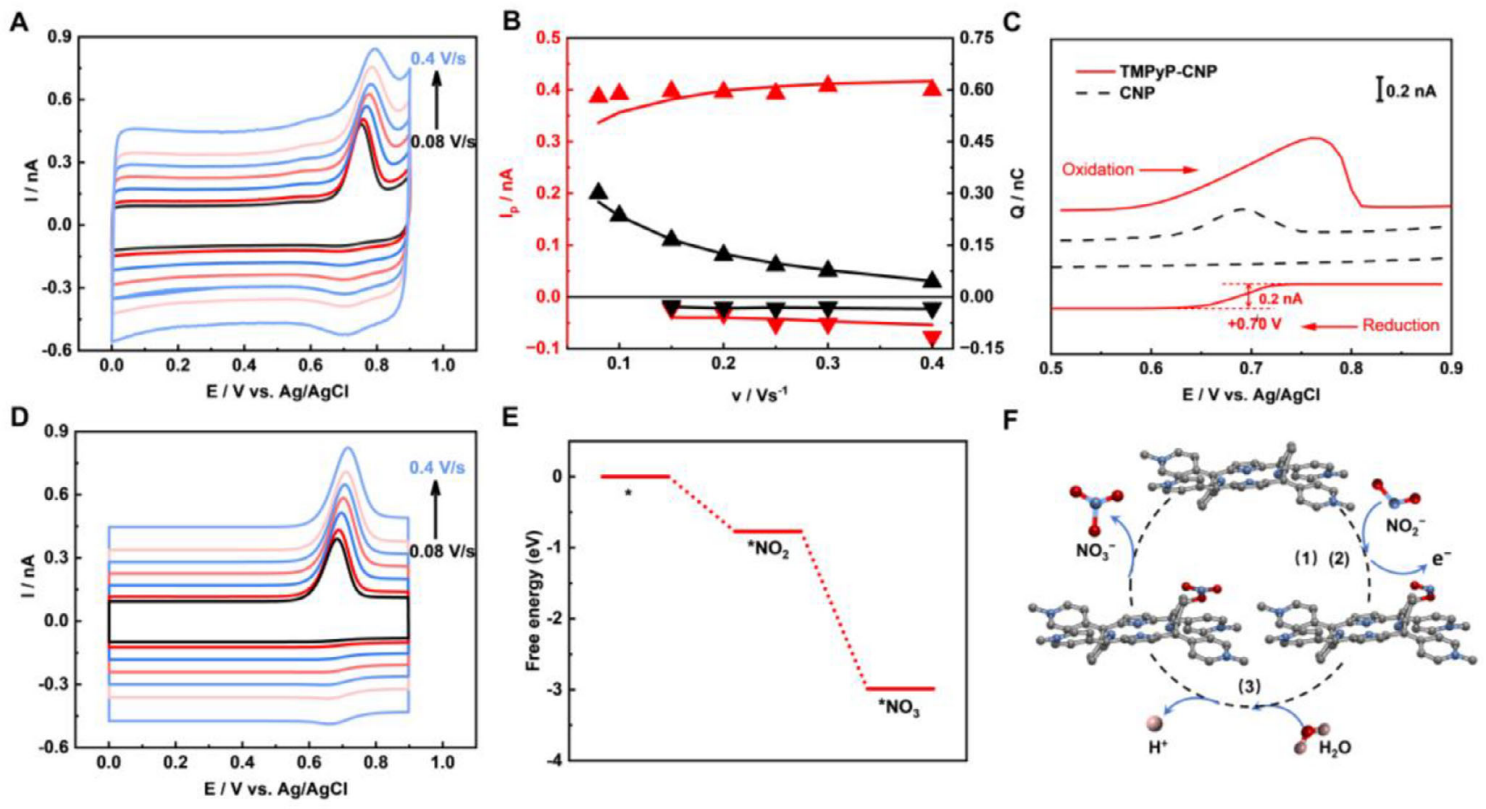
A) Experimental CVs of the TMPyP‐CNP at different *v*: 0.08, 0.1, 0.15, 0.2, 0.25, 0.3, and 0.4 V s^−1^, in 1 mm NaNO_2_ and 10 mm PBS. B) Experimental (dots) and simulated (curves) oxidation peak current (red) and enclosed charge (black) at various *v* for the TMPyP‐CNP. C) Normal Pulse Voltammograms of the TMPyP‐CNP and CNP. D) Simulated CVs based on a CEC model. E) DFT‐calculated free energy paths and F) proposed structures of the NO_2_^−^ oxidation at TMPyP‐CNPs. Gray, blue, red, and pink spheres represent C, N, O, and H, respectively.

As RNS are prone to rapid interconversion, the NO_2_^−^ oxidation kinetics were also studied by varying scan rates (*v*). Very intriguingly, the peak current plateaued at a broad range of scan rates (**Figure** **3**A,B), suggesting a possible chemical reaction‐controlled processes.^[^ ^42^ ^]^ Meanwhile, a progressively increasing reduction peak ≈ +0.7 V starts to superimpose when *v* > 0.15 V s^−1^, indicating an unstable intermediate get reduced. In comparison, at the CNP, the oxidation peak current linearly increases with *v*, and no reduction peaks are observed (Figure S7, Supporting Information). To confirm the transient feature, the normal pulse voltammetry (NPV) was conducted (Figure 3C). At the oxidation scan, the nitrite oxidation peak is clearly displayed at both CNP and TMPyP‐CNP, while a well‐defined shoulder ≈ +0.7 V only exists at the TMPyP‐CNP during the reduction scan, suggesting the TMPyP could stabilize the intermediate to undergo the following reduction processes.

Since porphyrin could effectively catalyze the oxidation of NO_2_^−^ to NO_3_^−^ via the NO_2_ intermediate,^[^ ^43^, ^44^, ^45^ ^]^ the reduction peaks are believed to be from NO_2_ reduction. In this case, NO_2_^−^ would first be oxidized into NO_2_, and then either react with H_2_O to form NO_2_^−^ to NO_3_^−^, or undergo electrochemical reduction into NO_2_^−^, depending on the time scale or *v*. At high *v*, NO_2_ do not have enough time to completely react with H_2_O, thus its reduction peaks are observed at the reverse scans; while at low *v*, all NO_2_ have already reacted with H_2_O, and no reduction peaks would be resulted. Overall, we propose a CEC reaction mechanism for the nitrite oxidation at the TMPyP‐CNP (the * represents the active sites in TMPyP):

(1) $$*+N{O_{2}}^{-}⇋\left[*\left(N{O_{2}}^{-}\right)\right]$$

(2) $$\left[*\left(N{O_{2}}^{-}\right)\right]-e^{-}⇋\left[*\left(NO_{2}\right)\right]$$

(3) $$2\left[*\left(NO_{2}\right)\right]+H_{2}O⇋2H^{+}+\left[*N{O_{2}}^{-}\right]+\left[*N{O_{3}}^{-}\right]$$

With the proposed CEC mechanism, numerical simulation was conducted in the TMPyP‐CNP, and similar CV responses were obtained (Figure 3D). Interestingly, both the oxidation and reduction peak current at various *v* can be decently fitted with the CEC model (Figure 3B), and the kinetics can then be extracted. The plateaued current indicates the surface adsorption is the rate‐determining step, with a fitted rate of 0.1 s^−1^. Meanwhile, the estimated life‐time of adsorbed *NO_2_ is ≈2.67 s, which is much longer than the typical radical lifetime of sub‐ms (see Supporting Information for more calculation details).^[^ ^46^ ^]^ Next, density functional theory (DFT) calculations were performed to better understand the catalytic nitrite oxidation processes at TMPyP. As shown in Figure 3E and Table S2 (Supporting Information), the adsorption of NO_2_^−^ on the active site of TMPyP (Equation 1) is an exothermic process, with a free energy change of −0.77 eV. Moreover, the calculated free energy for the formation of *NO_3_^−^ on TMPyP (−3.11 eV) was even more negative. Taken together, the DFT results highlight the *β*‐carbon as the active site in TMPyP, responsible for its exceptional electrocatalytic performance in the oxidation of NO_2_^−^.

**Figure 4:**
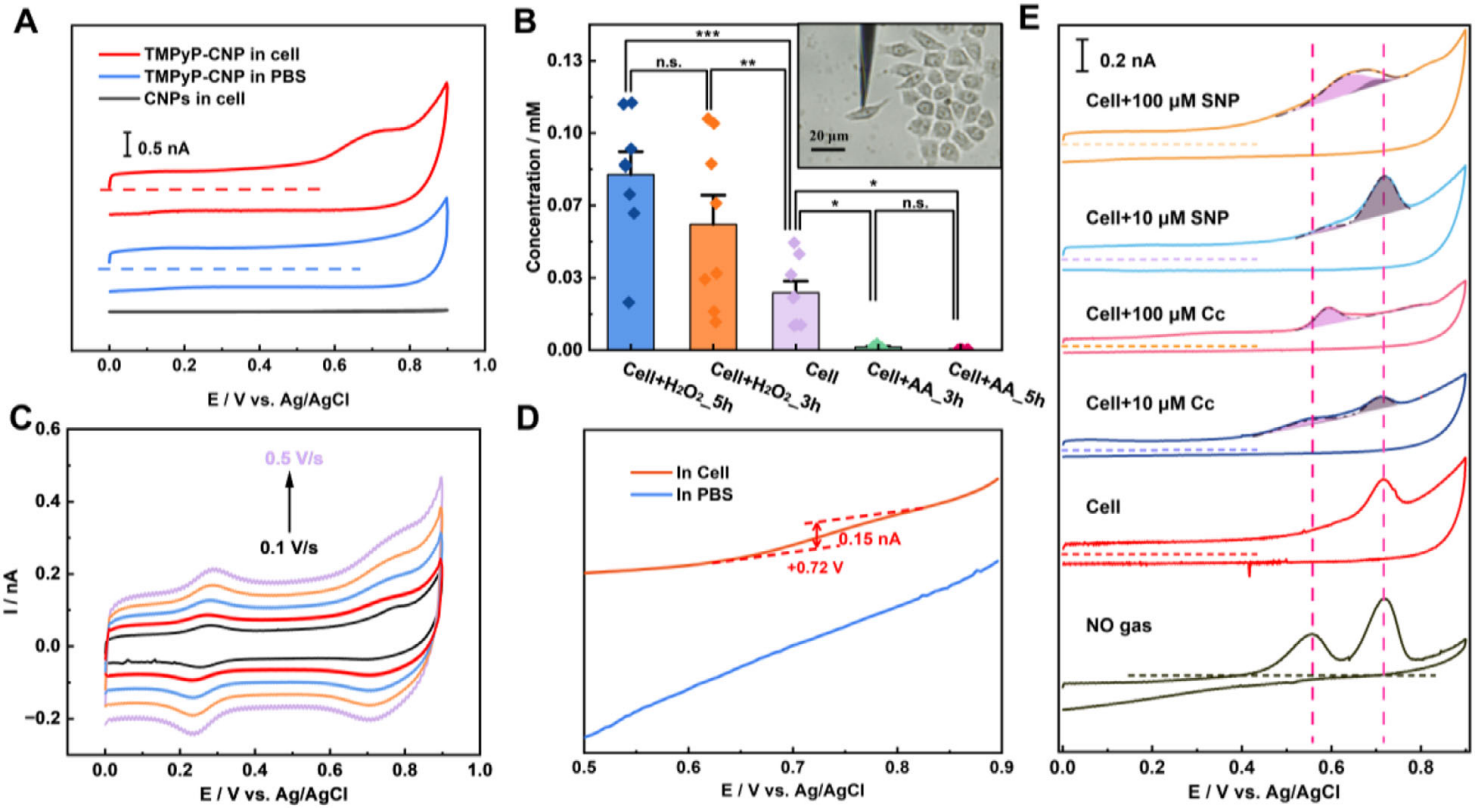
The cell experiments: A) CVs of the CNP and TMPyP‐CNP in HeLa cell. B) Statistical studies of RNS level in HeLa cells exposed to H_2_O_2_ and VC (n = 8, means ± SEM; one‐way ANOVA, ^***^ *p* ≤ 0.001, ^**^ *p* ≤ 0.01, ^*^ *p* ≤ 0.05, n.s.: not significant). Inset: optical image of electrodes in a single living cell. C) CVs of the TMPyP‐CNP at different scan rates. D) NPVs of the TMPyP‐CNP in cell and PBS. E) CVs of TMPyP‐CNPs in cells with respective treatments. *Cc* is cytochrome *c* and SNP is sodium nitroprusside.

Then, the TMPyP‐CNP was inserted into a single living HeLa cell to measure the intracellular RNS. Shown in **Figure** **4**A, it produces a broad oxidation peak round +0.7 V, indicating the oxidation of nitrogen species, most likely both NO and NO_2_^−^. To further confirm the origin of this oxidation peak, we modulated intracellular RNS levels by adding 10 µm H_2_O_2_ or vitamin C, to induce an elevated and reduced intracellular RNS concentration, respectively.^[^ ^47^ ^]^ The integrated charges enclosed in the oxidation are summarized (Figure S8, Supporting Information), and the respective RNS concentrations are calculated (Figure 4B). As expected, compared to unmedicated HeLa cells, RNS concentrations increased from 26 to 56 and 79 µm, at 3 and 5 h post‐treatment of H_2_O_2_, respectively. Conversely, when 0.1 µm VC was added, intracellular RNS concentrations markedly decreased to nearly zero, demonstrating the effectiveness of vitamin C in reducing RNS levels after oxidative stress. Note that the TMPyP‐CNP has no response to H_2_O_2_, which could largely exclude the contribution from ROS in cells. Then, the RNS oxidation kinetic in the cells are also studied by varying the *v* (Figure 4C). The same reduction peaks at +0.72 V are displayed at the reduction scans, ascribing to the NO_2_ reduction. It is worth pointing out that, due to the heterogeneity in the electrodes and cells, sometimes such NO_2_ reduction peaks are not obvious in the CVs (Figure S9, Supporting Information), while the NPV could always capture such NO_2_ signal in the cells (Figure 4D).

To further reveal the essential RNS interconversion pathways inside the living cell, we stimulated cells with given agents (Figure 4E). Cytochrome *c* (*Cc*)^[^ ^48^ ^]^ is known to stimulate living cells to generate NO by eliminating NO_2_^−^,^[^ ^49^ ^]^ and sodium nitroprusside (SNP) is one typical drug that produces NO.^[^ ^50^ ^]^ As expected, after incubating cells with 10 µm *Cc*, the pronounced NO oxidation peak is shown at 0.6 V, with a decreased NO_2_^−^ oxidation peak at 0.72 V. The higher Cc concentration of 100 µm would further intensify the NO peak and decrease NO_2_^−^ peak. Differently, when cells were incubated with SNP for 1 h, enhanced NO production was observed, with a retained NO_2_^−^ oxidation peak. These results demonstrate that the TMPyP‐CNP can effectively detect NO, NO_2_^−^, and their dynamic interconversions within single living cells via simple voltammetry studies. The ability to monitor rapid changes in RNS highlights the potential of TMPyP‐CNPs for studying dynamic NO‐related cellular processes.

## 3 Conclusion

In summary, by utilizing the thin‐layer electrochemistry inside the porphyrin‐modified carbon nanopipettes (TMPyP‐CNP), we apply transient voltammetry studies inside the single living cells and reveal the dynamic interconversion processes between intracellular RNS. The TMPyP‐CNP displays a very good sensing ability toward the NO and NO_2_^−^, and the intermediate NO_2_ in both solution and single living cells. A CEC mechanism for NO_2_^−^ oxidation via a NO_2_ intermediate is then proposed to well explain the interesting plateaued oxidation peak and transient reduction peak at high scan rates. Moreover, the TMPyP‐CNPs provide real‐time monitoring of intracellular RNS concentration and dynamic interconversions, thus shedding new light on the cellular redox processes. This work advances beyond traditional steady‐state methods by establishing a powerful nanoelectrode platform for studying transient biological reactions, with significant implications for understanding fundamental redox biology and developing novel biosensing applications.

## Conflict of Interest

The authors declare no conflict of interest.

## Supporting information



Supporting Information

Supporting Information

## Data Availability

The data that support the findings of this study are available from the corresponding author upon reasonable request.
