# Supplementary material for: Interconversions between RNS Revealed by Transient Voltammetry with Porphyrin‐Modified Carbon Nanopipettes in Single Living Cells
Source: Adv Sci (Weinh). 2025 Oct 6;12(48):e12865. doi: 10.1002/advs.202512865 (PMC12752564; doi:10.1002/advs.202512865)
Supplement: Supplementary file 1 — Supporting Information [file ADVS-12-e12865-s001.docx]

[
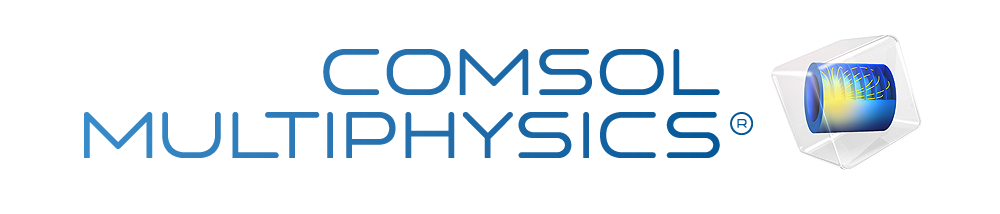
](https://www.comsol.com/)

CNP-CEC

| Report date | Apr 30, 2025 2:18:37 PM |
| --- | --- |

Contents

[1 Global Definitions 3](#_Toc210479024)

[1.1 Parameters 3](#_Toc210479025)

[2 Component 1 5](#_Toc210479026)

[2.1 Definitions 5](#_Toc210479027)

[2.2 Geometry 1 6](#_Toc210479028)

[2.3 Transport of Diluted Species 7](#_Toc210479029)

[2.4 Mesh 1 28](#_Toc210479030)

[3 Study 1 33](#_Toc210479031)

[3.1 Time Dependent 33](#_Toc210479032)

[3.2 Solver Configurations 34](#_Toc210479033)

[4 Results 38](#_Toc210479034)

[4.1 Data Sets 38](#_Toc210479035)

[4.2 Derived Values 39](#_Toc210479036)

[4.3 Plot Groups 40](#_Toc210479037)

1. Global Definitions

| Date | Mar 16, 2021 8:39:51 PM |
| --- | --- |

Global settings

| Name | CNP different reversibility.mph |
| --- | --- |
| Path | D:\simulation-other\CHL\CNP different reversibility.mph |
| Version | COMSOL Multiphysics 5.6 (Build: 280) |

Used products

| COMSOL Multiphysics |
| --- |
| Chemical Reaction Engineering Module |

Computer information

| CPU | Intel64 Family 6 Model 170 Stepping 4, 22 cores |
| --- | --- |
| Operating system | Windows 10 |

- 1. Parameters

Parameters 1

| **Name** | **Expression** | **Value** | **Description** |
| --- | --- | --- | --- |
| a | 100[nm] | 1E−7 m |  |
| depth | l*a | 8E−5 m |  |
| tan_theta | 0.1 | 0.1 |  |
| thickness | 5[nm] | 5E−9 m |  |
| k0 | 0.005[cm/s] | 5E−5 m/s |  |
| E0 | 0.7 [V] | 0.7 V |  |
| E1 | 0[V] | 0 V |  |
| F | 96485[C/mol] | 96485 C/mol |  |
| c0 | 0.01 | 0.01 |  |
| l | 800 | 800 |  |
| v | 0.3 | 0.3 |  |
| k2 | k0 | 5E−5 m/s |  |
| k3 | 0.1[1/s] | 0.1 1/s |  |
| k4 | 0[1/s] | 0 1/s |  |
| k1 | k0 | 5E−5 m/s |  |
| k8 | 1000000[L/(mol*s)] | 1000 m³/(s·mol) |  |
| k9 | 0.1[L/(mol*s)] | 1E−4 m³/(s·mol) |  |
| k5 | 5[1/s] | 5 1/s |  |
| k6 | 0.01[1/s] | 0.01 1/s |  |

1. Component 1

Settings

| **Description** | **Value** |
| --- | --- |
| Unit system | Same as global system (SI) |
| Avoid inverted elements by curving interior domain elements | Off |

- 1. Definitions
     1. Variables

#### Variables 1

Selection

| Geometric entity level | Entire model |
| --- | --- |

| **Name** | **Expression** | **Unit** | **Description** |
| --- | --- | --- | --- |
| E | 1[V]*wv1(t/1[s]) + 0.45 | V |  |

- - 1. Functions

#### Waveform 1

| Function name | wv1 |
| --- | --- |
| Function type | Waveform |


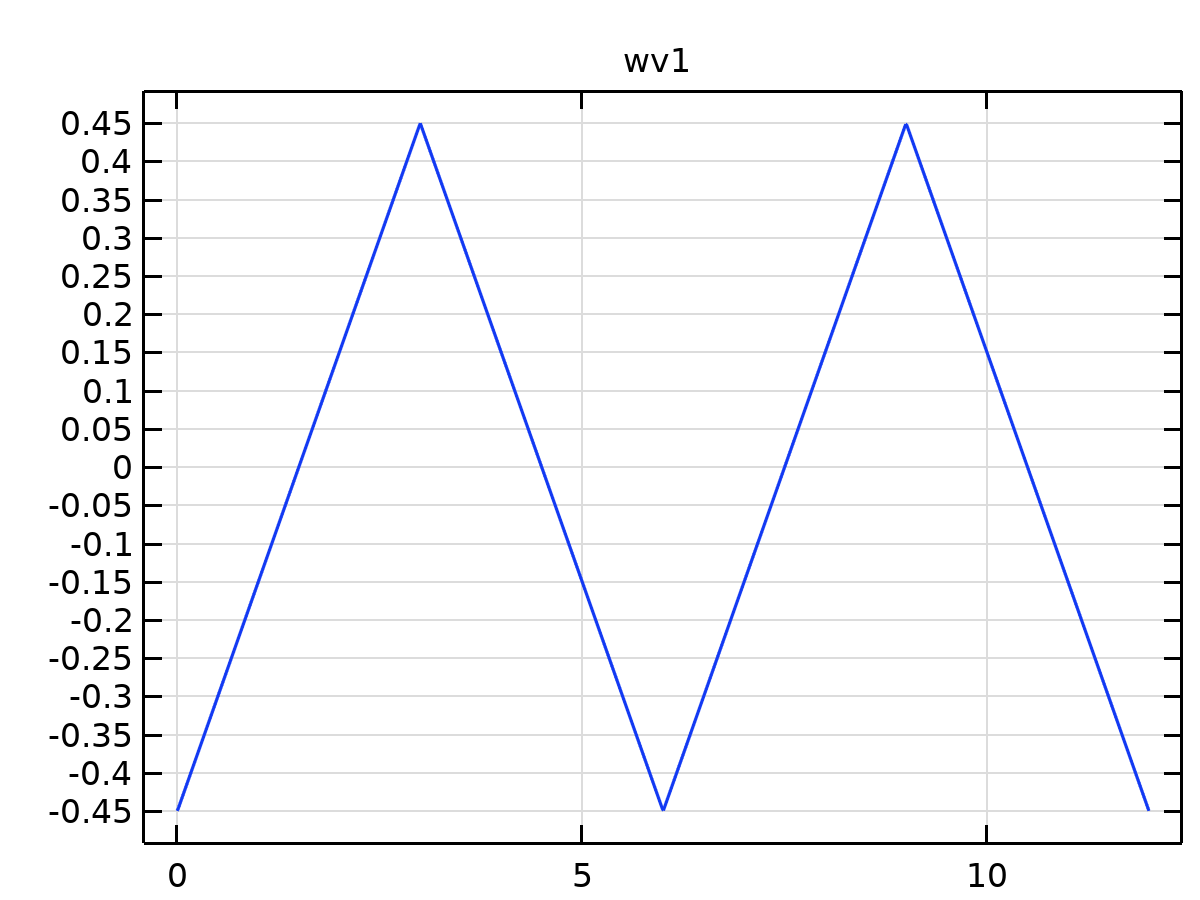


Waveform 1

Parameters

| **Description** | **Value** |
| --- | --- |
| Type | Triangle |
| Size of transition zone | 0.0001 |
| Angular frequency | v*pi/0.9 |
| Phase | -1.573 |
| Amplitude | 0.45 |

- - 1. Coordinate Systems

#### Boundary System 1

| Coordinate system type | Boundary system |
| --- | --- |
| Tag | sys1 |

Coordinate names

| **First** | **Second** | **Third** |
| --- | --- | --- |
| t1 | to | n |

- 1. Geometry 1


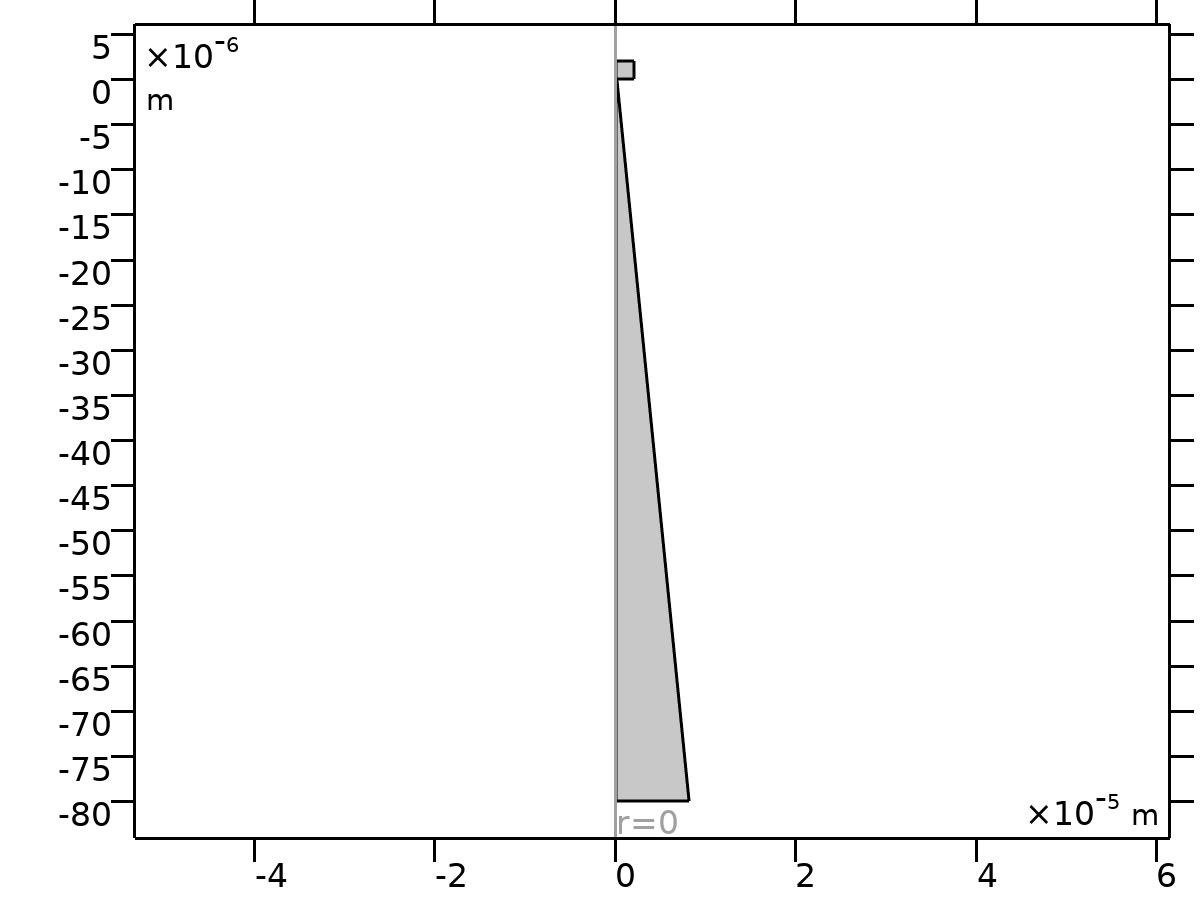


Geometry 1

Units

| Length unit | m |
| --- | --- |
| Angular unit | deg |

Geometry statistics

| **Description** | **Value** |
| --- | --- |
| Space dimension | 2 |
| Number of domains | 1 |
| Number of boundaries | 7 |
| Number of vertices | 7 |

- - 1. Polygon 1 (pol1)

Object type

| **Description** | **Value** |
| --- | --- |
| Type | Solid |

Coordinates

| **Description** | **Value** |
| --- | --- |
| Data source | Table |

Coordinates

| **r (m)** | **z (m)** |
| --- | --- |
| 0 | 2e-6 |
| 2e-6 | 2e-6 |
| 2e-6 | 0 |
| a | 0 |
| tan_theta*depth+a | -depth |
| 0 | -depth |

- - 1. Point 1 (pt1)

Point

| **Description** | **Value** |
| --- | --- |
| Point coordinate | {1.0500000000000001E-7, 0} |

- 1. Transport of Diluted Species

Used products

| COMSOL Multiphysics |
| --- |
| Chemical Reaction Engineering Module |


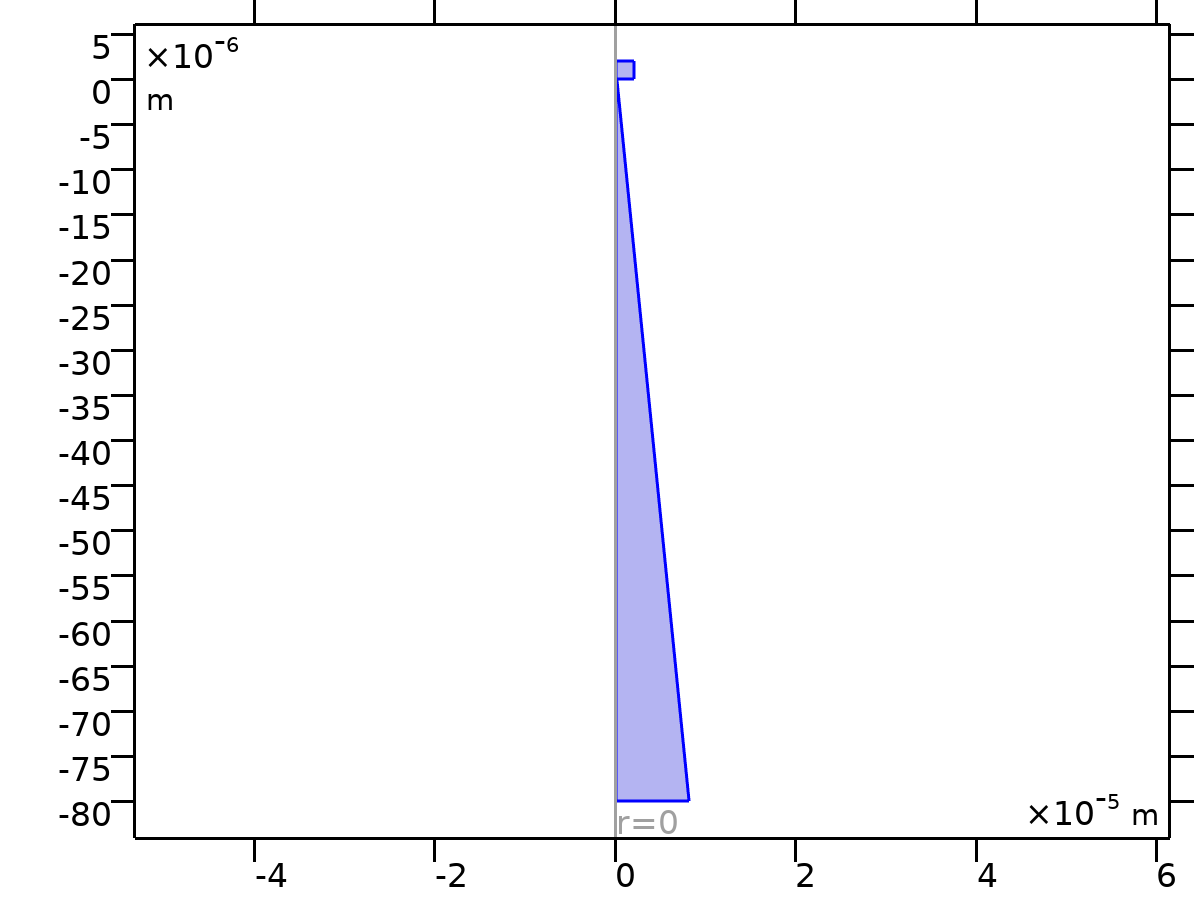


Transport of Diluted Species

Selection

| Geometric entity level | Domain |
| --- | --- |
| Selection | Geometry geom1: Dimension 2: Domain 1 |

Equations


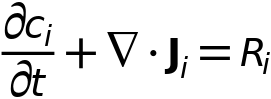


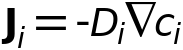


- - 1. Interface Settings

#### Discretization

Settings

| **Description** | **Value** |
| --- | --- |
| Concentration | Linear |

#### Transport Mechanisms

Settings

| **Description** | **Value** |
| --- | --- |
| Convection | Off |
| Migration in electric field | Off |
| Mass transfer in porous media | Off |

- - 1. Variables

| **Name** | **Expression** | **Unit** | **Description** | **Selection** | **Details** |
| --- | --- | --- | --- | --- | --- |
| tds.R_cO | 0 | mol/(m³·s) | Total rate expression | Domain 1 | + operation |
| tds.cP_cO | 0 | mol/kg | Concentration species absorbed to the solid | Domain 1 | + operation |
| tds.cP_cO | 0 | mol/kg | Concentration species absorbed to the solid | Boundaries 1–7 | + operation |
| tds.KP_cO | 0 | m³/kg | Adsorption isotherm, first concentration derivative | Domain 1 | + operation |
| tds.KP_cO | 0 | m³/kg | Adsorption isotherm, first concentration derivative | Boundaries 1–7 | + operation |
| tds.R_cR | 0 | mol/(m³·s) | Total rate expression | Domain 1 | + operation |
| tds.cP_cR | 0 | mol/kg | Concentration species absorbed to the solid | Domain 1 | + operation |
| tds.cP_cR | 0 | mol/kg | Concentration species absorbed to the solid | Boundaries 1–7 | + operation |
| tds.KP_cR | 0 | m³/kg | Adsorption isotherm, first concentration derivative | Domain 1 | + operation |
| tds.KP_cR | 0 | m³/kg | Adsorption isotherm, first concentration derivative | Boundaries 1–7 | + operation |
| tds.R_cA | 0 | mol/(m³·s) | Total rate expression | Domain 1 | + operation |
| tds.cP_cA | 0 | mol/kg | Concentration species absorbed to the solid | Domain 1 | + operation |
| tds.cP_cA | 0 | mol/kg | Concentration species absorbed to the solid | Boundaries 1–7 | + operation |
| tds.KP_cA | 0 | m³/kg | Adsorption isotherm, first concentration derivative | Domain 1 | + operation |
| tds.KP_cA | 0 | m³/kg | Adsorption isotherm, first concentration derivative | Boundaries 1–7 | + operation |
| tds.R_cB | 0 | mol/(m³·s) | Total rate expression | Domain 1 | + operation |
| tds.cP_cB | 0 | mol/kg | Concentration species absorbed to the solid | Domain 1 | + operation |
| tds.cP_cB | 0 | mol/kg | Concentration species absorbed to the solid | Boundaries 1–7 | + operation |
| tds.KP_cB | 0 | m³/kg | Adsorption isotherm, first concentration derivative | Domain 1 | + operation |
| tds.KP_cB | 0 | m³/kg | Adsorption isotherm, first concentration derivative | Boundaries 1–7 | + operation |
| tds.poro | 1 | 1 | Porosity | Domain 1 |  |
| tds.theta_g | 0 | 1 | Gas volume fraction | Domain 1 |  |
| tds.theta | tds.poro | 1 | Mobile fluid volume fraction | Domain 1 |  |
| tds.nr | dnr | 1 | Normal vector, r component | Boundaries 1–7 |  |
| tds.nphi | 0 | 1 | Normal vector, phi component | Boundaries 1–7 |  |
| tds.nz | dnz | 1 | Normal vector, z component | Boundaries 1–7 |  |
| tds.nrmesh | dnrmesh | 1 | Normal vector (mesh), r component | Boundaries 1–7 |  |
| tds.nphimesh | 0 | 1 | Normal vector (mesh), phi component | Boundaries 1–7 |  |
| tds.nzmesh | dnzmesh | 1 | Normal vector (mesh), z component | Boundaries 1–7 |  |
| tds.nrc | root.nrc/tds.ncLen | 1 | Normal vector, r component | Boundaries 1–7 |  |
| tds.nphic | 0 | 1 | Normal vector, phi component | Boundaries 1–7 |  |
| tds.nzc | root.nzc/tds.ncLen | 1 | Normal vector, z component | Boundaries 1–7 |  |
| tds.ncLen | sqrt(root.nrc^2+root.nzc^2+eps) | 1 | Help variable | Boundaries 1–7 |  |

- - 1. Transport Properties 1


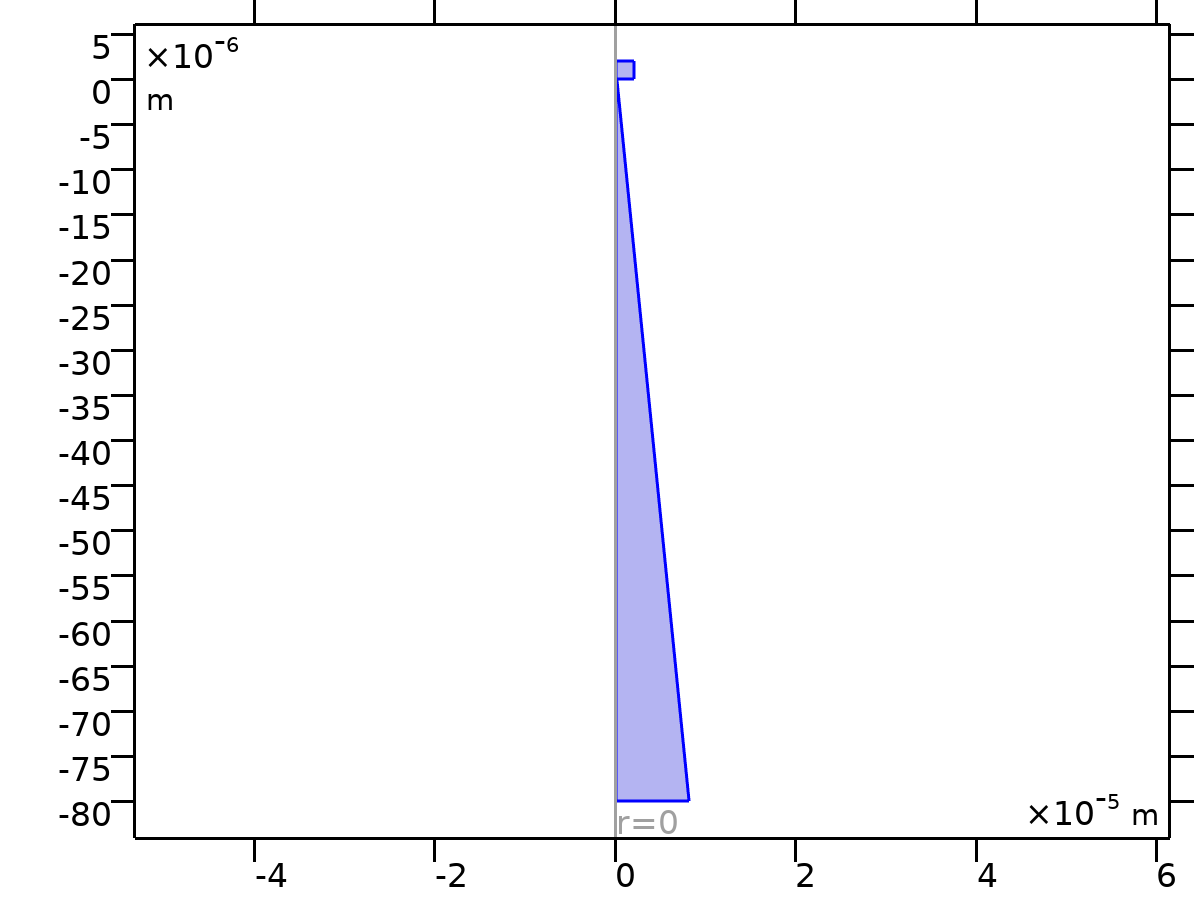


Transport Properties 1

Selection

| Geometric entity level | Domain |
| --- | --- |
| Selection | Geometry geom1: Dimension 2: All domains |

Equations


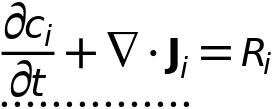


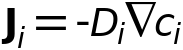


#### Diffusion

Settings

| **Description** | **Value** |
| --- | --- |
| Source | Material |
| Material | None |
| Diffusion coefficient | User defined |
| Diffusion coefficient | {{7.8e-10[m^2/s], 0, 0}, {0, 7.8e-10[m^2/s], 0}, {0, 0, 7.8e-10[m^2/s]}} |
| Diffusion coefficient | User defined |
| Diffusion coefficient | {{7.8e-10[m^2/s], 0, 0}, {0, 7.8e-10[m^2/s], 0}, {0, 0, 7.8e-10[m^2/s]}} |
| Diffusion coefficient | User defined |
| Diffusion coefficient | {{7.8e-10[m^2/s], 0, 0}, {0, 7.8e-10[m^2/s], 0}, {0, 0, 7.8e-10[m^2/s]}} |
| Diffusion coefficient | User defined |
| Diffusion coefficient | {{7.8e-10[m^2/s], 0, 0}, {0, 7.8e-10[m^2/s], 0}, {0, 0, 7.8e-10[m^2/s]}} |

#### Coordinate System Selection

Settings

| **Description** | **Value** |
| --- | --- |
| Coordinate system | Global coordinate system |

#### Model Input

Settings

| **Description** | **Value** |
| --- | --- |
| Temperature | Common model input |

#### Variables

| **Name** | **Expression** | **Unit** | **Description** | **Selection** | **Details** |
| --- | --- | --- | --- | --- | --- |
| domflux.cOr | 2*tds.dflux_cOr*pi*r | mol/(m·s) | Domain flux, r component | Domain 1 |  |
| domflux.cOz | 2*tds.dflux_cOz*pi*r | mol/(m·s) | Domain flux, z component | Domain 1 |  |
| domflux.cRr | 2*tds.dflux_cRr*pi*r | mol/(m·s) | Domain flux, r component | Domain 1 |  |
| domflux.cRz | 2*tds.dflux_cRz*pi*r | mol/(m·s) | Domain flux, z component | Domain 1 |  |
| domflux.cAr | 2*tds.dflux_cAr*pi*r | mol/(m·s) | Domain flux, r component | Domain 1 |  |
| domflux.cAz | 2*tds.dflux_cAz*pi*r | mol/(m·s) | Domain flux, z component | Domain 1 |  |
| domflux.cBr | 2*tds.dflux_cBr*pi*r | mol/(m·s) | Domain flux, r component | Domain 1 |  |
| domflux.cBz | 2*tds.dflux_cBz*pi*r | mol/(m·s) | Domain flux, z component | Domain 1 |  |
| tds.ndflux_cO | tds.bndFlux_cO | mol/(m²·s) | Normal diffusive flux | Boundaries 2–7 |  |
| tds.ntflux_cO | tds.bndFlux_cO | mol/(m²·s) | Normal total flux | Boundaries 2–7 |  |
| tds.ndflux_cR | tds.bndFlux_cR | mol/(m²·s) | Normal diffusive flux | Boundaries 2–7 |  |
| tds.ntflux_cR | tds.bndFlux_cR | mol/(m²·s) | Normal total flux | Boundaries 2–7 |  |
| tds.ndflux_cA | tds.bndFlux_cA | mol/(m²·s) | Normal diffusive flux | Boundaries 2–7 |  |
| tds.ntflux_cA | tds.bndFlux_cA | mol/(m²·s) | Normal total flux | Boundaries 2–7 |  |
| tds.ndflux_cB | tds.bndFlux_cB | mol/(m²·s) | Normal diffusive flux | Boundaries 2–7 |  |
| tds.ntflux_cB | tds.bndFlux_cB | mol/(m²·s) | Normal total flux | Boundaries 2–7 |  |
| tds.bndFlux_cO | if(r>0.001/sqrt(sqrt(mean(emetric2))),-0.5*dflux_spatial(cO)/(pi*r),NaN) | mol/(m²·s) | Boundary flux | Boundaries 1–7 |  |
| tds.bndFlux_cR | if(r>0.001/sqrt(sqrt(mean(emetric2))),-0.5*dflux_spatial(cR)/(pi*r),NaN) | mol/(m²·s) | Boundary flux | Boundaries 1–7 |  |
| tds.bndFlux_cA | if(r>0.001/sqrt(sqrt(mean(emetric2))),-0.5*dflux_spatial(cA)/(pi*r),NaN) | mol/(m²·s) | Boundary flux | Boundaries 1–7 |  |
| tds.bndFlux_cB | if(r>0.001/sqrt(sqrt(mean(emetric2))),-0.5*dflux_spatial(cB)/(pi*r),NaN) | mol/(m²·s) | Boundary flux | Boundaries 1–7 |  |
| tds.D_cOrr | 7.8E-10[m^2/s] | m²/s | Diffusion coefficient, rr component | Domain 1 |  |
| tds.D_cOphir | 0 | m²/s | Diffusion coefficient, phir component | Domain 1 |  |
| tds.D_cOzr | 0 | m²/s | Diffusion coefficient, zr component | Domain 1 |  |
| tds.D_cOrphi | 0 | m²/s | Diffusion coefficient, rphi component | Domain 1 |  |
| tds.D_cOphiphi | 7.8E-10[m^2/s] | m²/s | Diffusion coefficient, phiphi component | Domain 1 |  |
| tds.D_cOzphi | 0 | m²/s | Diffusion coefficient, zphi component | Domain 1 |  |
| tds.D_cOrz | 0 | m²/s | Diffusion coefficient, rz component | Domain 1 |  |
| tds.D_cOphiz | 0 | m²/s | Diffusion coefficient, phiz component | Domain 1 |  |
| tds.D_cOzz | 7.8E-10[m^2/s] | m²/s | Diffusion coefficient, zz component | Domain 1 |  |
| tds.D_cRrr | 7.8E-10[m^2/s] | m²/s | Diffusion coefficient, rr component | Domain 1 |  |
| tds.D_cRphir | 0 | m²/s | Diffusion coefficient, phir component | Domain 1 |  |
| tds.D_cRzr | 0 | m²/s | Diffusion coefficient, zr component | Domain 1 |  |
| tds.D_cRrphi | 0 | m²/s | Diffusion coefficient, rphi component | Domain 1 |  |
| tds.D_cRphiphi | 7.8E-10[m^2/s] | m²/s | Diffusion coefficient, phiphi component | Domain 1 |  |
| tds.D_cRzphi | 0 | m²/s | Diffusion coefficient, zphi component | Domain 1 |  |
| tds.D_cRrz | 0 | m²/s | Diffusion coefficient, rz component | Domain 1 |  |
| tds.D_cRphiz | 0 | m²/s | Diffusion coefficient, phiz component | Domain 1 |  |
| tds.D_cRzz | 7.8E-10[m^2/s] | m²/s | Diffusion coefficient, zz component | Domain 1 |  |
| tds.D_cArr | 7.8E-10[m^2/s] | m²/s | Diffusion coefficient, rr component | Domain 1 |  |
| tds.D_cAphir | 0 | m²/s | Diffusion coefficient, phir component | Domain 1 |  |
| tds.D_cAzr | 0 | m²/s | Diffusion coefficient, zr component | Domain 1 |  |
| tds.D_cArphi | 0 | m²/s | Diffusion coefficient, rphi component | Domain 1 |  |
| tds.D_cAphiphi | 7.8E-10[m^2/s] | m²/s | Diffusion coefficient, phiphi component | Domain 1 |  |
| tds.D_cAzphi | 0 | m²/s | Diffusion coefficient, zphi component | Domain 1 |  |
| tds.D_cArz | 0 | m²/s | Diffusion coefficient, rz component | Domain 1 |  |
| tds.D_cAphiz | 0 | m²/s | Diffusion coefficient, phiz component | Domain 1 |  |
| tds.D_cAzz | 7.8E-10[m^2/s] | m²/s | Diffusion coefficient, zz component | Domain 1 |  |
| tds.D_cBrr | 7.8E-10[m^2/s] | m²/s | Diffusion coefficient, rr component | Domain 1 |  |
| tds.D_cBphir | 0 | m²/s | Diffusion coefficient, phir component | Domain 1 |  |
| tds.D_cBzr | 0 | m²/s | Diffusion coefficient, zr component | Domain 1 |  |
| tds.D_cBrphi | 0 | m²/s | Diffusion coefficient, rphi component | Domain 1 |  |
| tds.D_cBphiphi | 7.8E-10[m^2/s] | m²/s | Diffusion coefficient, phiphi component | Domain 1 |  |
| tds.D_cBzphi | 0 | m²/s | Diffusion coefficient, zphi component | Domain 1 |  |
| tds.D_cBrz | 0 | m²/s | Diffusion coefficient, rz component | Domain 1 |  |
| tds.D_cBphiz | 0 | m²/s | Diffusion coefficient, phiz component | Domain 1 |  |
| tds.D_cBzz | 7.8E-10[m^2/s] | m²/s | Diffusion coefficient, zz component | Domain 1 |  |
| tds.Dav_cO | 0.5*(tds.D_cOrr+tds.D_cOzz) | m²/s | Average diffusion coefficient | Domain 1 |  |
| tds.Dav_cR | 0.5*(tds.D_cRrr+tds.D_cRzz) | m²/s | Average diffusion coefficient | Domain 1 |  |
| tds.Dav_cA | 0.5*(tds.D_cArr+tds.D_cAzz) | m²/s | Average diffusion coefficient | Domain 1 |  |
| tds.Dav_cB | 0.5*(tds.D_cBrr+tds.D_cBzz) | m²/s | Average diffusion coefficient | Domain 1 |  |
| tds.tflux_cOr | tds.dflux_cOr | mol/(m²·s) | Total flux, r component | Domain 1 | + operation |
| tds.tflux_cOphi | tds.dflux_cOphi | mol/(m²·s) | Total flux, phi component | Domain 1 | + operation |
| tds.tflux_cOz | tds.dflux_cOz | mol/(m²·s) | Total flux, z component | Domain 1 | + operation |
| tds.dfluxMag_cO | sqrt(tds.dflux_cOr^2+tds.dflux_cOphi^2+tds.dflux_cOz^2) | mol/(m²·s) | Diffusive flux magnitude | Domain 1 |  |
| tds.tfluxMag_cO | sqrt(tds.tflux_cOr^2+tds.tflux_cOphi^2+tds.tflux_cOz^2) | mol/(m²·s) | Total flux magnitude | Domain 1 |  |
| tds.dpflux_cOr | 0 | mol/(m²·s) | Dispersive flux, r component | Domain 1 |  |
| tds.dpflux_cOphi | 0 | mol/(m²·s) | Dispersive flux, phi component | Domain 1 |  |
| tds.dpflux_cOz | 0 | mol/(m²·s) | Dispersive flux, z component | Domain 1 |  |
| tds.tflux_cRr | tds.dflux_cRr | mol/(m²·s) | Total flux, r component | Domain 1 | + operation |
| tds.tflux_cRphi | tds.dflux_cRphi | mol/(m²·s) | Total flux, phi component | Domain 1 | + operation |
| tds.tflux_cRz | tds.dflux_cRz | mol/(m²·s) | Total flux, z component | Domain 1 | + operation |
| tds.dfluxMag_cR | sqrt(tds.dflux_cRr^2+tds.dflux_cRphi^2+tds.dflux_cRz^2) | mol/(m²·s) | Diffusive flux magnitude | Domain 1 |  |
| tds.tfluxMag_cR | sqrt(tds.tflux_cRr^2+tds.tflux_cRphi^2+tds.tflux_cRz^2) | mol/(m²·s) | Total flux magnitude | Domain 1 |  |
| tds.dpflux_cRr | 0 | mol/(m²·s) | Dispersive flux, r component | Domain 1 |  |
| tds.dpflux_cRphi | 0 | mol/(m²·s) | Dispersive flux, phi component | Domain 1 |  |
| tds.dpflux_cRz | 0 | mol/(m²·s) | Dispersive flux, z component | Domain 1 |  |
| tds.tflux_cAr | tds.dflux_cAr | mol/(m²·s) | Total flux, r component | Domain 1 | + operation |
| tds.tflux_cAphi | tds.dflux_cAphi | mol/(m²·s) | Total flux, phi component | Domain 1 | + operation |
| tds.tflux_cAz | tds.dflux_cAz | mol/(m²·s) | Total flux, z component | Domain 1 | + operation |
| tds.dfluxMag_cA | sqrt(tds.dflux_cAr^2+tds.dflux_cAphi^2+tds.dflux_cAz^2) | mol/(m²·s) | Diffusive flux magnitude | Domain 1 |  |
| tds.tfluxMag_cA | sqrt(tds.tflux_cAr^2+tds.tflux_cAphi^2+tds.tflux_cAz^2) | mol/(m²·s) | Total flux magnitude | Domain 1 |  |
| tds.dpflux_cAr | 0 | mol/(m²·s) | Dispersive flux, r component | Domain 1 |  |
| tds.dpflux_cAphi | 0 | mol/(m²·s) | Dispersive flux, phi component | Domain 1 |  |
| tds.dpflux_cAz | 0 | mol/(m²·s) | Dispersive flux, z component | Domain 1 |  |
| tds.tflux_cBr | tds.dflux_cBr | mol/(m²·s) | Total flux, r component | Domain 1 | + operation |
| tds.tflux_cBphi | tds.dflux_cBphi | mol/(m²·s) | Total flux, phi component | Domain 1 | + operation |
| tds.tflux_cBz | tds.dflux_cBz | mol/(m²·s) | Total flux, z component | Domain 1 | + operation |
| tds.dfluxMag_cB | sqrt(tds.dflux_cBr^2+tds.dflux_cBphi^2+tds.dflux_cBz^2) | mol/(m²·s) | Diffusive flux magnitude | Domain 1 |  |
| tds.tfluxMag_cB | sqrt(tds.tflux_cBr^2+tds.tflux_cBphi^2+tds.tflux_cBz^2) | mol/(m²·s) | Total flux magnitude | Domain 1 |  |
| tds.dpflux_cBr | 0 | mol/(m²·s) | Dispersive flux, r component | Domain 1 |  |
| tds.dpflux_cBphi | 0 | mol/(m²·s) | Dispersive flux, phi component | Domain 1 |  |
| tds.dpflux_cBz | 0 | mol/(m²·s) | Dispersive flux, z component | Domain 1 |  |
| tds.dflux_cOr | -tds.D_cOrr*cOr-tds.D_cOrz*cOz | mol/(m²·s) | Diffusive flux, r component | Domain 1 |  |
| tds.dflux_cOphi | -tds.D_cOphir*cOr-tds.D_cOphiz*cOz | mol/(m²·s) | Diffusive flux, phi component | Domain 1 |  |
| tds.dflux_cOz | -tds.D_cOzr*cOr-tds.D_cOzz*cOz | mol/(m²·s) | Diffusive flux, z component | Domain 1 |  |
| tds.grad_cOr | cOr | mol/m⁴ | Concentration gradient, r component | Domain 1 |  |
| tds.grad_cOphi | 0 | mol/m⁴ | Concentration gradient, phi component | Domain 1 |  |
| tds.grad_cOz | cOz | mol/m⁴ | Concentration gradient, z component | Domain 1 |  |
| tds.dflux_cRr | -tds.D_cRrr*cRr-tds.D_cRrz*cRz | mol/(m²·s) | Diffusive flux, r component | Domain 1 |  |
| tds.dflux_cRphi | -tds.D_cRphir*cRr-tds.D_cRphiz*cRz | mol/(m²·s) | Diffusive flux, phi component | Domain 1 |  |
| tds.dflux_cRz | -tds.D_cRzr*cRr-tds.D_cRzz*cRz | mol/(m²·s) | Diffusive flux, z component | Domain 1 |  |
| tds.grad_cRr | cRr | mol/m⁴ | Concentration gradient, r component | Domain 1 |  |
| tds.grad_cRphi | 0 | mol/m⁴ | Concentration gradient, phi component | Domain 1 |  |
| tds.grad_cRz | cRz | mol/m⁴ | Concentration gradient, z component | Domain 1 |  |
| tds.dflux_cAr | -tds.D_cArr*cAr-tds.D_cArz*cAz | mol/(m²·s) | Diffusive flux, r component | Domain 1 |  |
| tds.dflux_cAphi | -tds.D_cAphir*cAr-tds.D_cAphiz*cAz | mol/(m²·s) | Diffusive flux, phi component | Domain 1 |  |
| tds.dflux_cAz | -tds.D_cAzr*cAr-tds.D_cAzz*cAz | mol/(m²·s) | Diffusive flux, z component | Domain 1 |  |
| tds.grad_cAr | cAr | mol/m⁴ | Concentration gradient, r component | Domain 1 |  |
| tds.grad_cAphi | 0 | mol/m⁴ | Concentration gradient, phi component | Domain 1 |  |
| tds.grad_cAz | cAz | mol/m⁴ | Concentration gradient, z component | Domain 1 |  |
| tds.dflux_cBr | -tds.D_cBrr*cBr-tds.D_cBrz*cBz | mol/(m²·s) | Diffusive flux, r component | Domain 1 |  |
| tds.dflux_cBphi | -tds.D_cBphir*cBr-tds.D_cBphiz*cBz | mol/(m²·s) | Diffusive flux, phi component | Domain 1 |  |
| tds.dflux_cBz | -tds.D_cBzr*cBr-tds.D_cBzz*cBz | mol/(m²·s) | Diffusive flux, z component | Domain 1 |  |
| tds.grad_cBr | cBr | mol/m⁴ | Concentration gradient, r component | Domain 1 |  |
| tds.grad_cBphi | 0 | mol/m⁴ | Concentration gradient, phi component | Domain 1 |  |
| tds.grad_cBz | cBz | mol/m⁴ | Concentration gradient, z component | Domain 1 |  |
| tds.Res_cO | d(cO,t)-tds.R_cO | mol/(m³·s) | Equation residual | Domain 1 |  |
| tds.Res_cR | d(cR,t)-tds.R_cR | mol/(m³·s) | Equation residual | Domain 1 |  |
| tds.Res_cA | d(cA,t)-tds.R_cA | mol/(m³·s) | Equation residual | Domain 1 |  |
| tds.Res_cB | d(cB,t)-tds.R_cB | mol/(m³·s) | Equation residual | Domain 1 |  |

#### Shape functions

| **Name** | **Shape function** | **Unit** | **Description** | **Shape frame** | **Selection** |
| --- | --- | --- | --- | --- | --- |
| cO | Lagrange (Linear) | mol/m³ | Concentration | Spatial | Domain 1 |
| cR | Lagrange (Linear) | mol/m³ | Concentration | Spatial | Domain 1 |
| cA | Lagrange (Linear) | mol/m³ | Concentration | Spatial | Domain 1 |
| cB | Lagrange (Linear) | mol/m³ | Concentration | Spatial | Domain 1 |

#### Weak Expressions

| **Weak expression** | **Integration order** | **Integration frame** | **Selection** |
| --- | --- | --- | --- |
| 2*(-cOt*test(cO)+tds.dflux_cOr*test(cOr)+tds.dflux_cOz*test(cOz))*pi*r | 2 | Spatial | Domain 1 |
| 2*(-cRt*test(cR)+tds.dflux_cRr*test(cRr)+tds.dflux_cRz*test(cRz))*pi*r | 2 | Spatial | Domain 1 |
| 2*(-cAt*test(cA)+tds.dflux_cAr*test(cAr)+tds.dflux_cAz*test(cAz))*pi*r | 2 | Spatial | Domain 1 |
| 2*(-cBt*test(cB)+tds.dflux_cBr*test(cBr)+tds.dflux_cBz*test(cBz))*pi*r | 2 | Spatial | Domain 1 |
| 2*tds.streamline*(isScalingSystemDomain==0)*pi*r | 2 | Spatial | Domain 1 |
| 2*tds.crosswind*(isScalingSystemDomain==0)*pi*r | 4 | Spatial | Domain 1 |

- - 1. Axial Symmetry 1


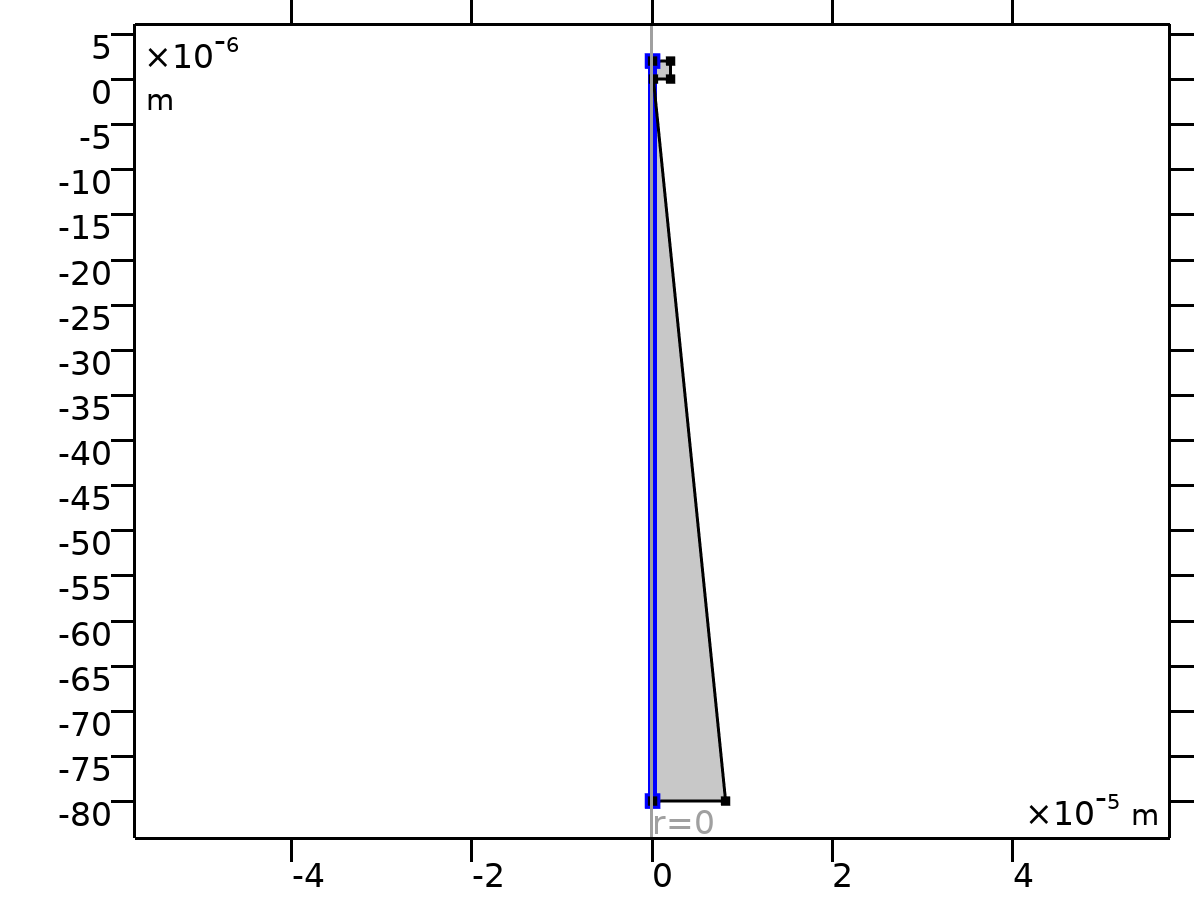


Axial Symmetry 1

Selection

| Geometric entity level | Boundary |
| --- | --- |
| Selection | Geometry geom1: Dimension 1: All boundaries |

- - 1. No Flux 1


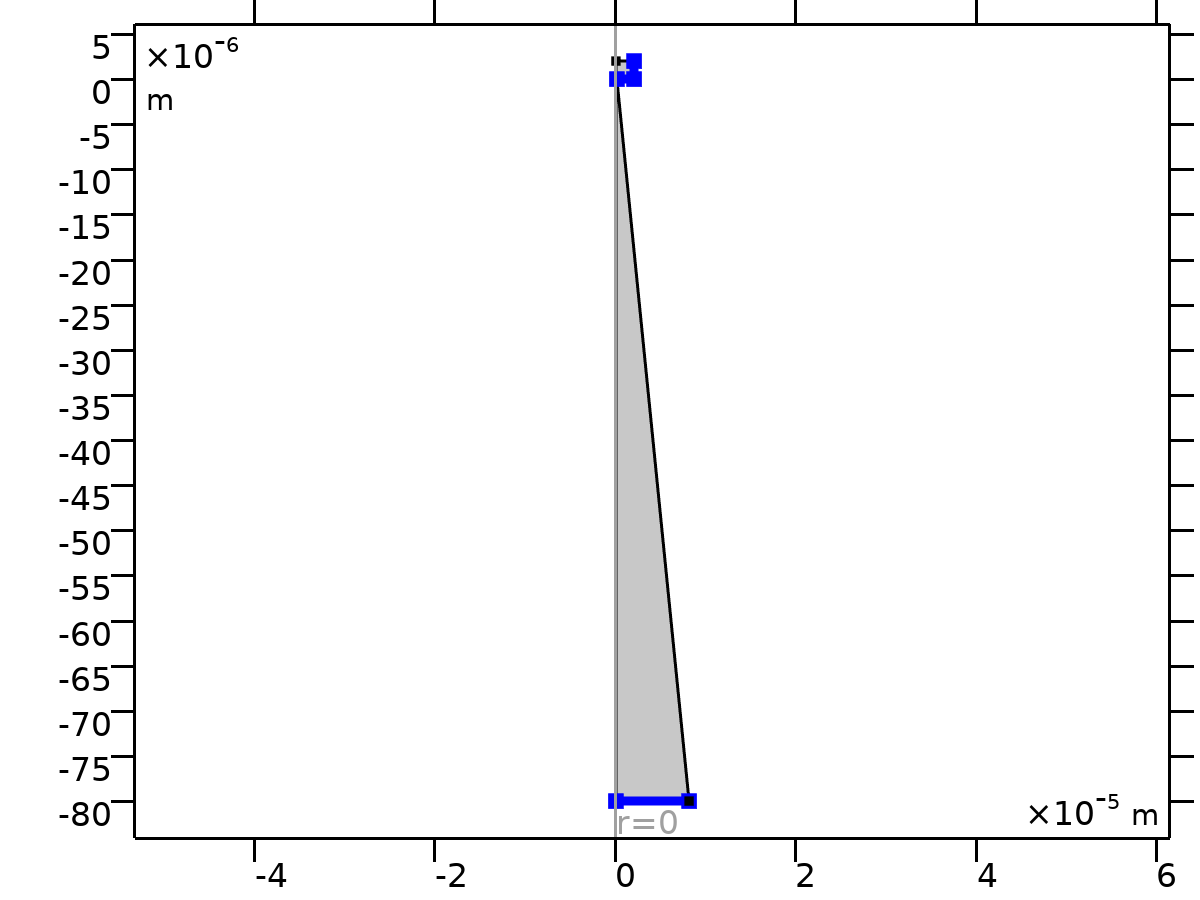


No Flux 1

Selection

| Geometric entity level | Boundary |
| --- | --- |
| Selection | Geometry geom1: Dimension 1: All boundaries |

Equations


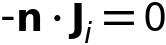


- - 1. Initial Values 1


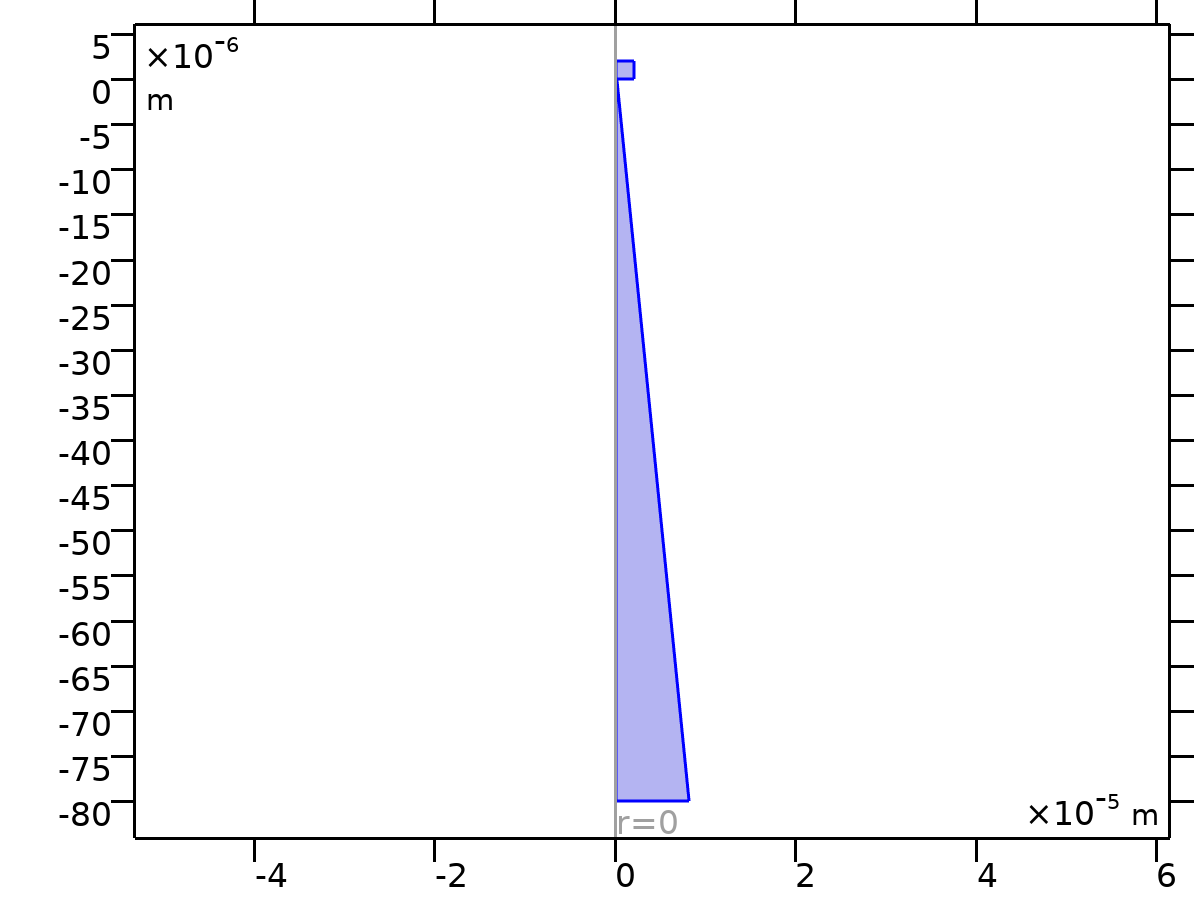


Initial Values 1

Selection

| Geometric entity level | Domain |
| --- | --- |
| Selection | Geometry geom1: Dimension 2: All domains |

#### Initial Values

Settings

| **Description** | **Value** |
| --- | --- |
| Concentration | {0, c0, 1, 0} |

- - 1. Flux 1


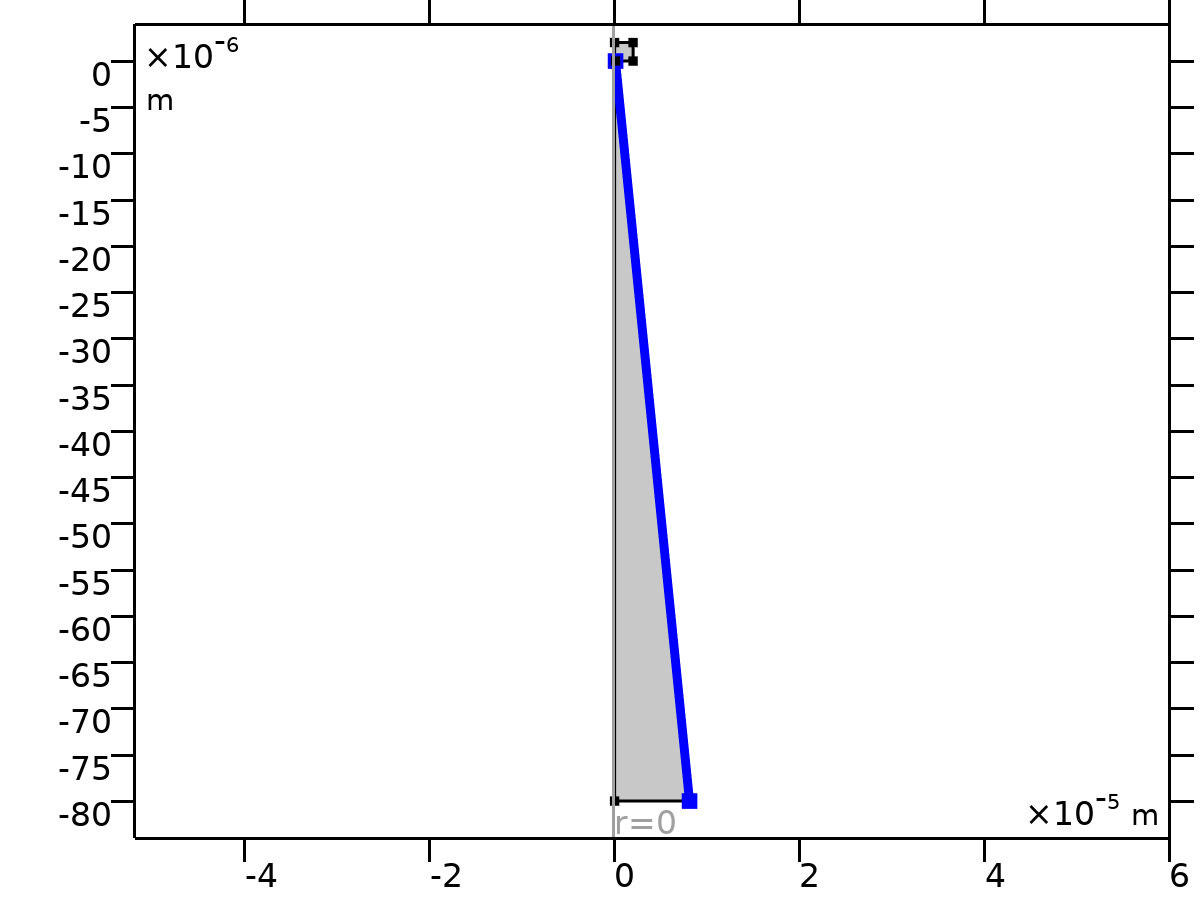


Flux 1

Selection

| Geometric entity level | Boundary |
| --- | --- |
| Selection | Geometry geom1: Dimension 1: Boundaries 4–5 |

Equations


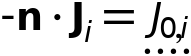


#### Inward Flux

Settings

| **Description** | **Value** |
| --- | --- |
| Flux type | General inward flux |
| Species cO | On |
| Species cR | On |
| Species cA | Off |
| Species cB | Off |
|  | {k1*cR*exp(0.5*38.9[1/V]*(E-E0)) - k2*cO*exp(-0.5*38.9[1/V]*(E-E0)), k2*cO*exp(-0.5*38.9[1/V]*(E-E0)) - k1*cR*exp(0.5*38.9[1/V]*(E-E0)), |

#### Weak Expressions

| **Weak expression** | **Integration order** | **Integration frame** | **Selection** |
| --- | --- | --- | --- |
| 2*(k1*cR*exp(0.5*38.9[1/V]*(E-E0))-k2*cO*exp(-0.5*38.9[1/V]*(E-E0)))*test(cO)*pi*r | 2 | Spatial | Boundaries 4–5 |
| 2*(k2*cO*exp(-0.5*38.9[1/V]*(E-E0))-k1*cR*exp(0.5*38.9[1/V]*(E-E0)))*test(cR)*pi*r | 2 | Spatial | Boundaries 4–5 |
| 0 | 2 | Spatial | Boundaries 4–5 |
| 0 | 2 | Spatial | Boundaries 4–5 |

- - 1. Concentration 1


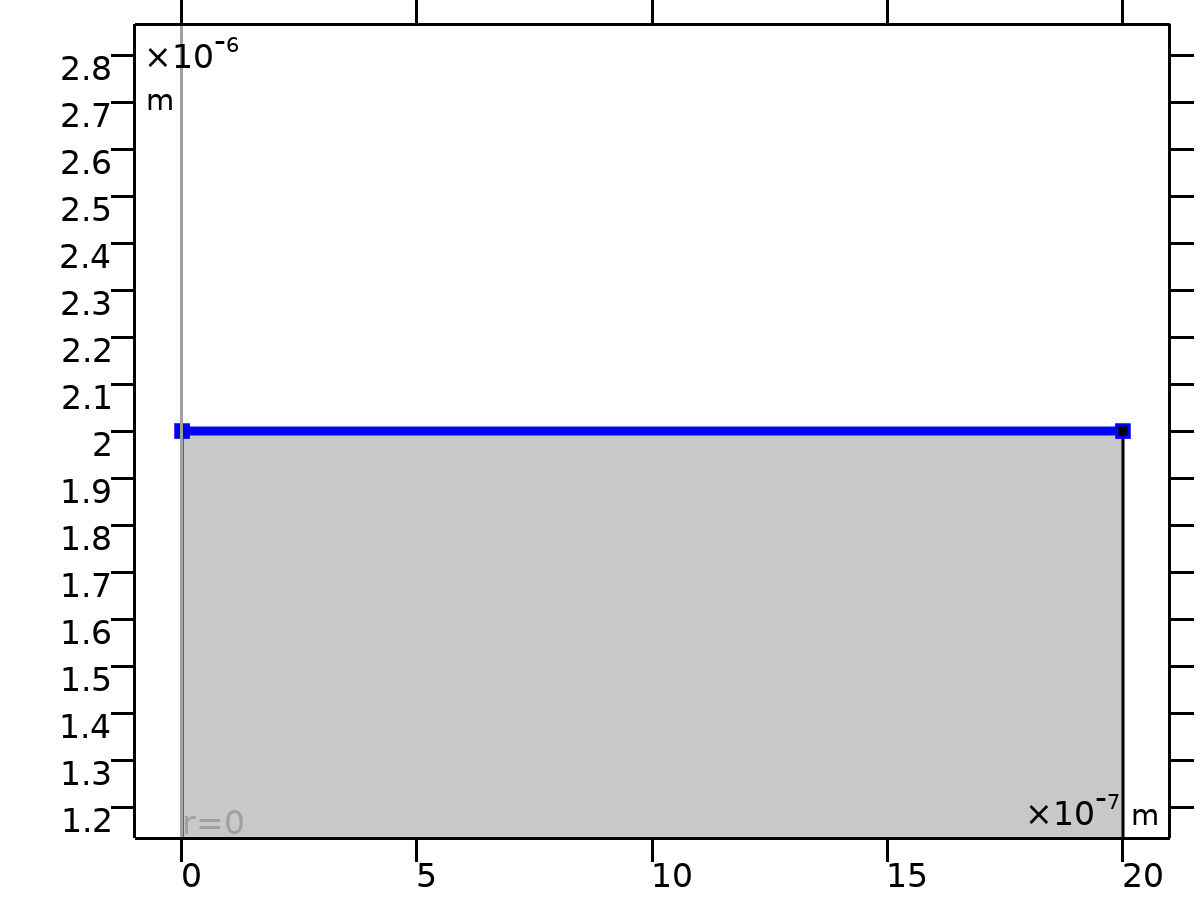


Concentration 1

Selection

| Geometric entity level | Boundary |
| --- | --- |
| Selection | Geometry geom1: Dimension 1: Boundary 3 |

Equations


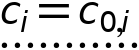


#### Concentration

Settings

| **Description** | **Value** |
| --- | --- |
| Species cO | On |
| Species cR | On |
| Species cA | On |
| Species cB | On |
| Concentration | {0, c0, 1, 0} |

#### Variables

| **Name** | **Expression** | **Unit** | **Description** | **Selection** |
| --- | --- | --- | --- | --- |
| tds.c0_cO | 0 | mol/m³ | Concentration | Boundary 3 |
| tds.c0_cR | c0 | mol/m³ | Concentration | Boundary 3 |
| tds.c0_cA | 1 | mol/m³ | Concentration | Boundary 3 |
| tds.c0_cB | 0 | mol/m³ | Concentration | Boundary 3 |

#### Constraints

| **Constraint** | **Constraint force** | **Shape function** | **Selection** | **Details** |
| --- | --- | --- | --- | --- |
| -cO+tds.c0_cO | test(-cO+tds.c0_cO) | Lagrange (Linear) | Boundary 3 | Elemental |
| -cR+tds.c0_cR | test(-cR+tds.c0_cR) | Lagrange (Linear) | Boundary 3 | Elemental |
| -cA+tds.c0_cA | test(-cA+tds.c0_cA) | Lagrange (Linear) | Boundary 3 | Elemental |
| -cB+tds.c0_cB | test(-cB+tds.c0_cB) | Lagrange (Linear) | Boundary 3 | Elemental |

- - 1. Reactions 1


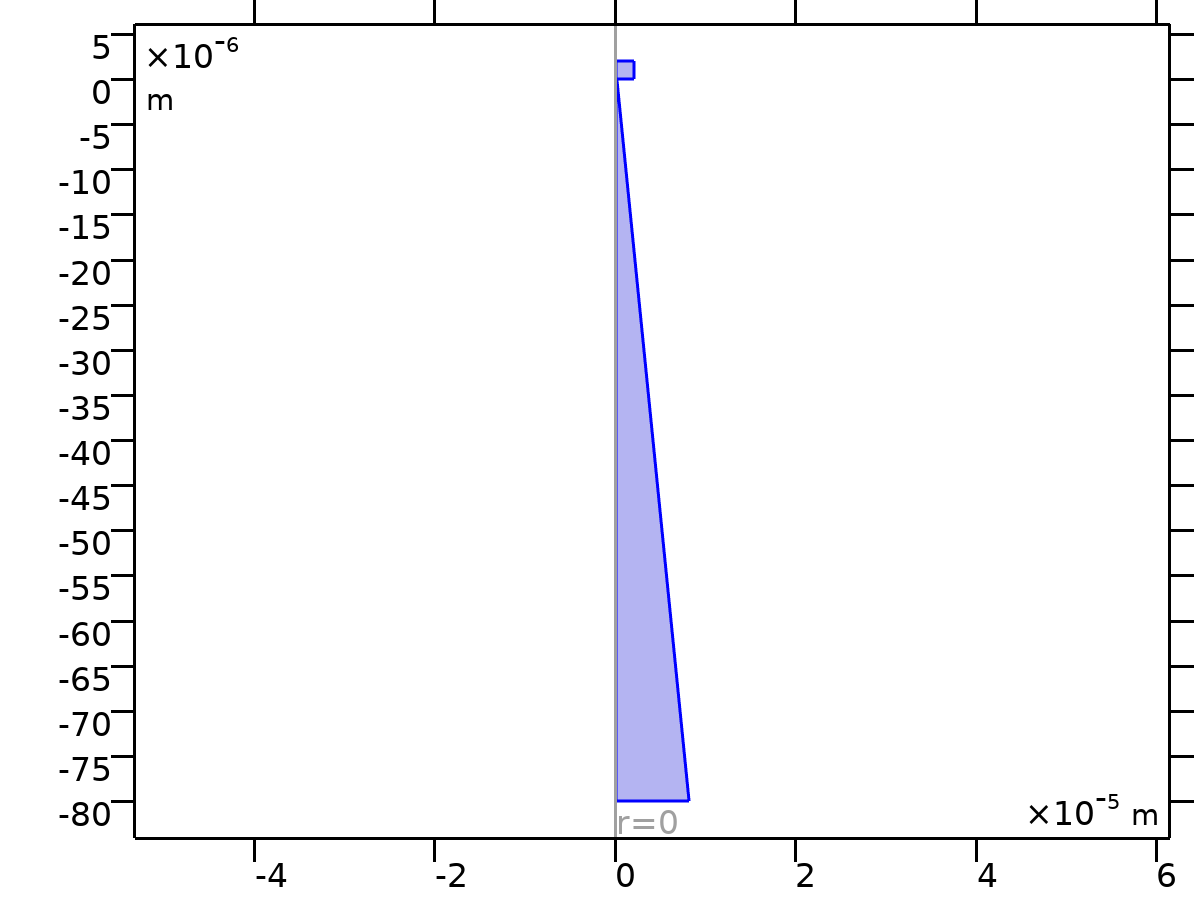


Reactions 1

Selection

| Geometric entity level | Domain |
| --- | --- |
| Selection | Geometry geom1: Dimension 2: Domain 1 |

Equations


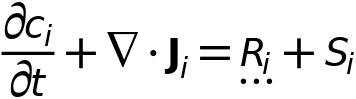


#### Reaction Rates

Settings

| **Description** | **Value** |
| --- | --- |
| Total rate expression | User defined |
| Total rate expression | -k5*cO + k6*cB |
| Total rate expression | User defined |
| Total rate expression | k3*cA - k4*cR + k6*cB |
| Total rate expression | User defined |
| Total rate expression | -k3*cA + k4*cR |
| Total rate expression | User defined |
| Total rate expression | k5*cO - k6*cB |

#### Variables

| **Name** | **Expression** | **Unit** | **Description** | **Selection** | **Details** |
| --- | --- | --- | --- | --- | --- |
| tds.R_cO | tds.reac1.R_cO | mol/(m³·s) | Total rate expression | Domain 1 | + operation |
| tds.R_cR | tds.reac1.R_cR | mol/(m³·s) | Total rate expression | Domain 1 | + operation |
| tds.R_cA | tds.reac1.R_cA | mol/(m³·s) | Total rate expression | Domain 1 | + operation |
| tds.R_cB | tds.reac1.R_cB | mol/(m³·s) | Total rate expression | Domain 1 | + operation |
| tds.reac1.R_cO | model.input.R_cO | mol/(m³·s) | Total rate expression | Domain 1 | Meta |
| tds.reac1.R_cR | model.input.R_cR | mol/(m³·s) | Total rate expression | Domain 1 | Meta |
| tds.reac1.R_cA | model.input.R_cA | mol/(m³·s) | Total rate expression | Domain 1 | Meta |
| tds.reac1.R_cB | model.input.R_cB | mol/(m³·s) | Total rate expression | Domain 1 | Meta |

#### Weak Expressions

| **Weak expression** | **Integration order** | **Integration frame** | **Selection** |
| --- | --- | --- | --- |
| 2*tds.reac1.R_cO*test(cO)*pi*r | 2 | Spatial | Domain 1 |
| 2*tds.reac1.R_cR*test(cR)*pi*r | 2 | Spatial | Domain 1 |
| 2*tds.reac1.R_cA*test(cA)*pi*r | 2 | Spatial | Domain 1 |
| 2*tds.reac1.R_cB*test(cB)*pi*r | 2 | Spatial | Domain 1 |

- 1. Mesh 1


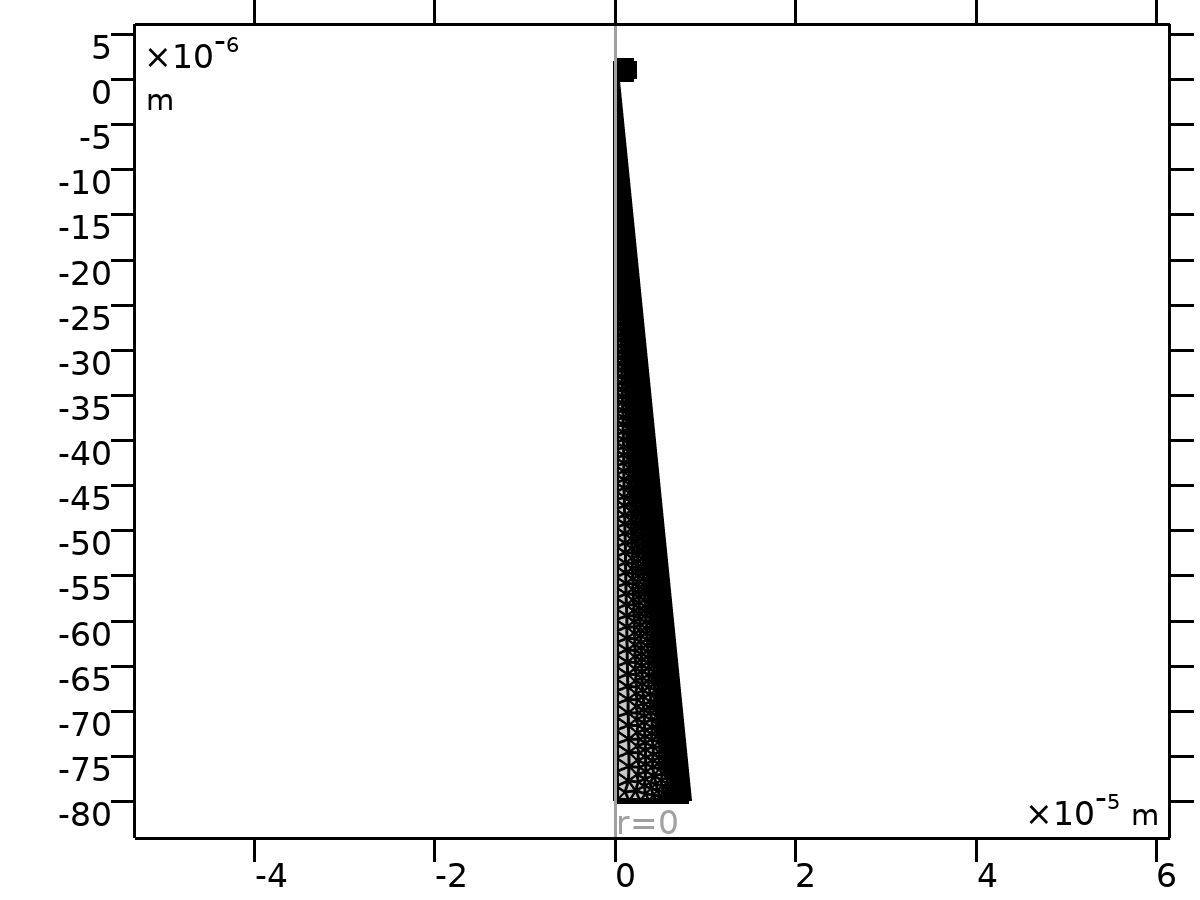


Mesh 1

- - 1. Size (size)

Settings

| **Description** | **Value** |
| --- | --- |
| Maximum element size | 1.64E-6 |
| Minimum element size | 6.15E-9 |
| Curvature factor | 0.25 |
| Maximum element growth rate | 1.2 |
| Predefined size | Extra fine |

- - 1. Size 1 (size1)

Selection

| Geometric entity level | Boundary |
| --- | --- |
| Selection | Geometry geom1: Dimension 1: Boundaries 4–5 |


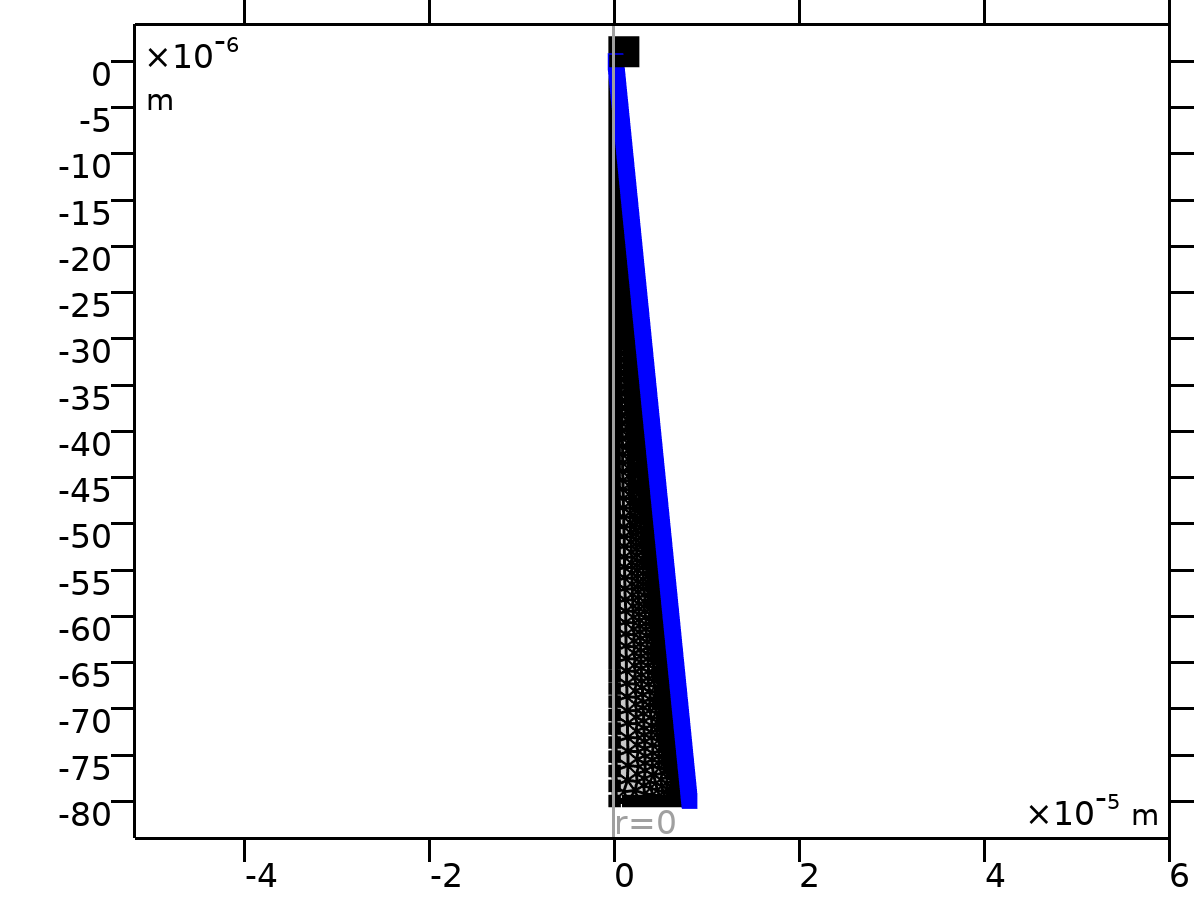


Size 1

Settings

| **Description** | **Value** |
| --- | --- |
| Maximum element size | 50[nm] |
| Minimum element size | 2.1E-9 |
| Minimum element size | Off |
| Curvature factor | 0.3 |
| Curvature factor | Off |
| Resolution of narrow regions | Off |
| Maximum element growth rate | 1.3 |
| Maximum element growth rate | Off |
| Custom element size | Custom |

- - 1. Size 2 (size2)

Selection

| Geometric entity level | Boundary |
| --- | --- |
| Selection | Geometry geom1: Dimension 1: Boundaries 3, 6–7 |


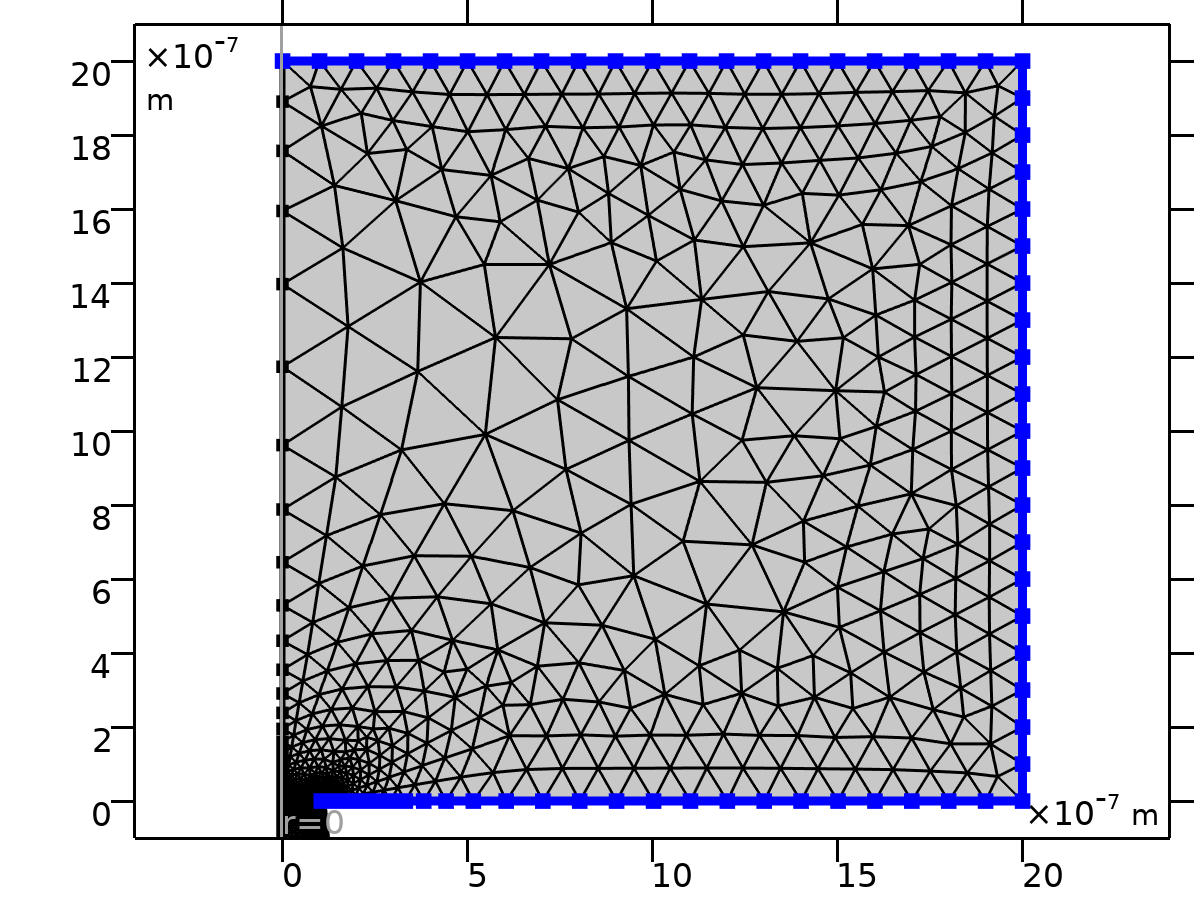


Size 2

Settings

| **Description** | **Value** |
| --- | --- |
| Maximum element size | 1e-7 |
| Minimum element size | 2.1E-9 |
| Minimum element size | Off |
| Curvature factor | 0.3 |
| Curvature factor | Off |
| Resolution of narrow regions | Off |
| Maximum element growth rate | 1.3 |
| Maximum element growth rate | Off |
| Custom element size | Custom |

- - 1. Size 3 (size3)

Selection

| Geometric entity level | Point |
| --- | --- |
| Selection | Geometry geom1: Dimension 0: Points 3–4 |


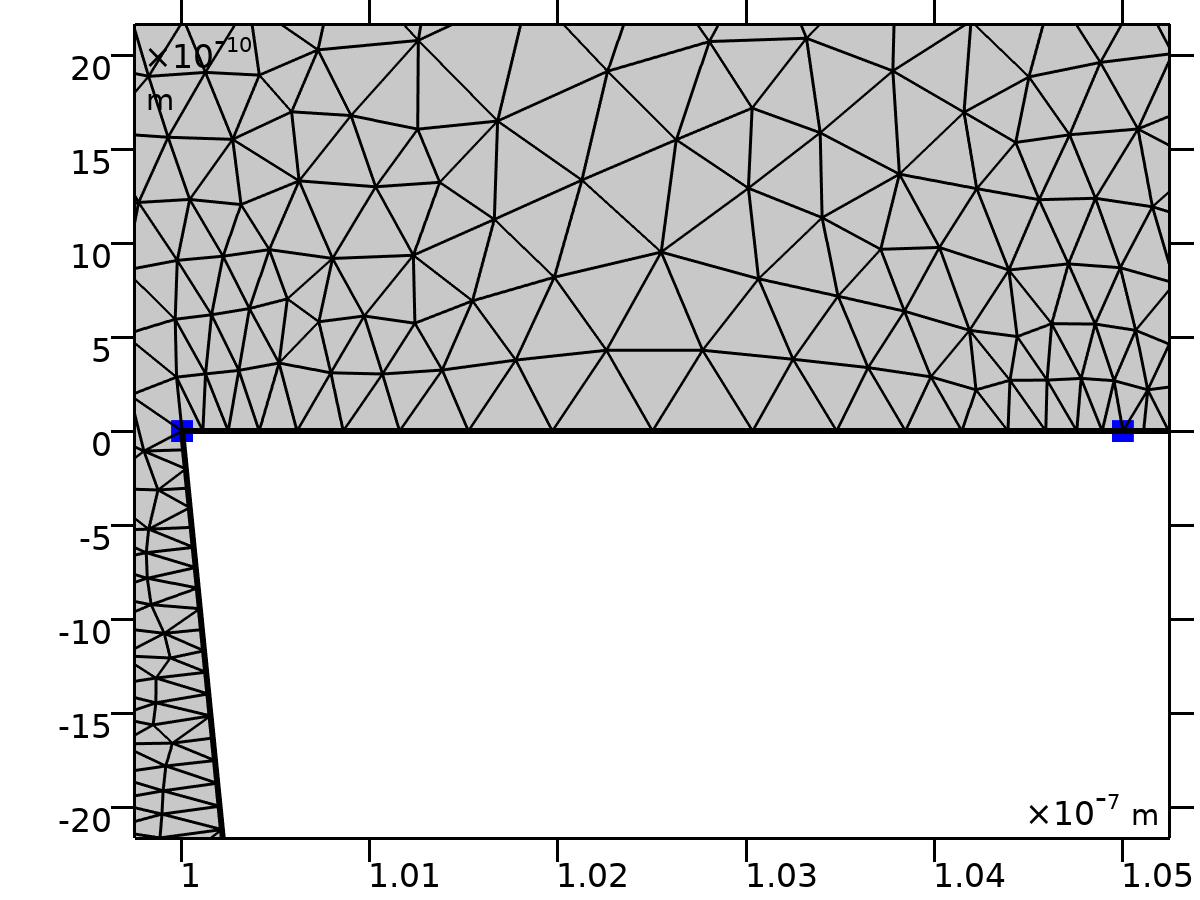


Size 3

Settings

| **Description** | **Value** |
| --- | --- |
| Maximum element size | 1e-10 |
| Minimum element size | 3.6E-9 |
| Minimum element size | Off |
| Curvature factor | 0.3 |
| Curvature factor | Off |
| Resolution of narrow regions | Off |
| Maximum element growth rate | 1.3 |
| Maximum element growth rate | Off |
| Custom element size | Custom |

- - 1. Free Triangular 1 (ftri1)

Selection

| Geometric entity level | Domain |
| --- | --- |
| Selection | Remaining |


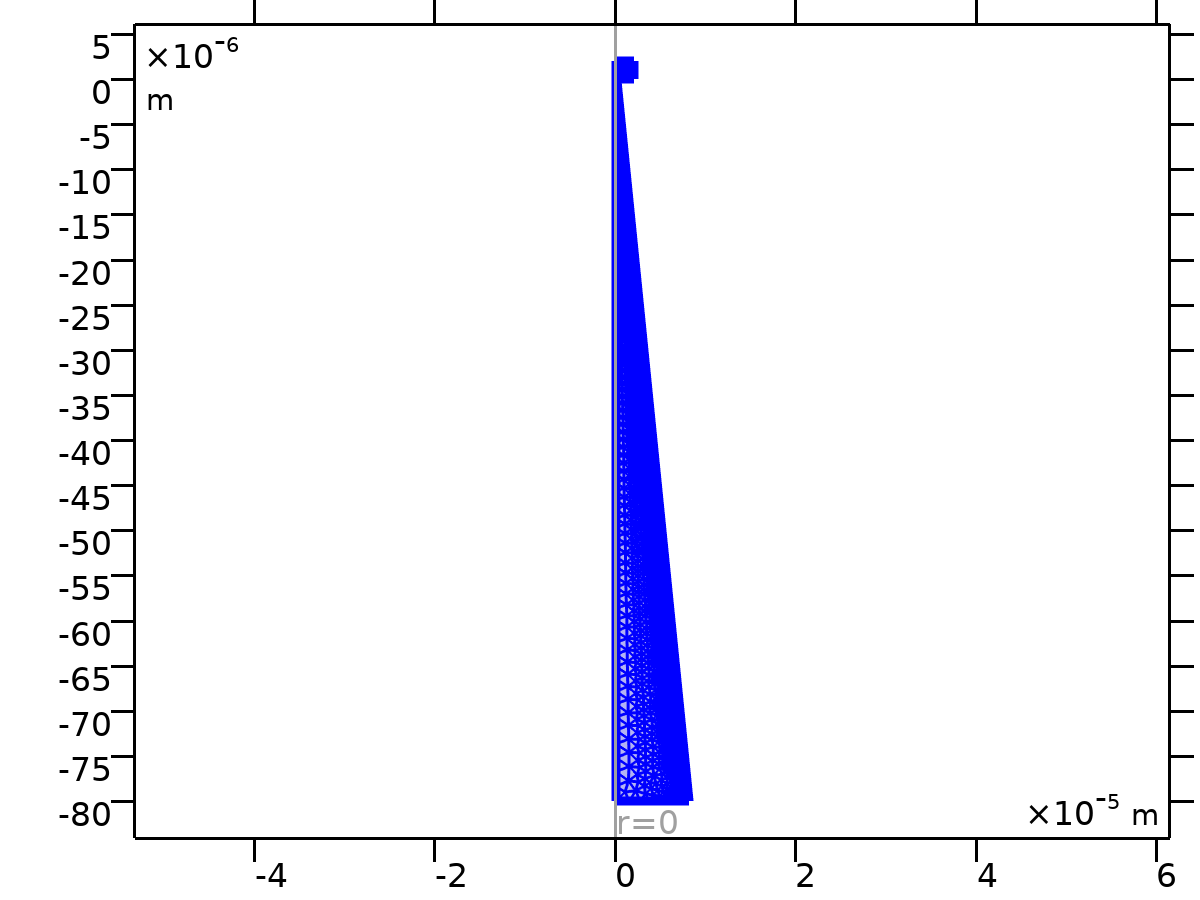


Free Triangular 1

1. Study 1

Computation information

| Computation time | 1 min 55 s |
| --- | --- |

- 1. Time Dependent

| **Times** | **Unit** |
| --- | --- |
| range(0,(3.6/v-0)/99,3.6/v) | s |

Study settings

| **Description** | **Value** |
| --- | --- |
| Include geometric nonlinearity | Off |

Study settings

| **Description** | **Value** |
| --- | --- |
| Output times | {0, 1.2121212121212124, 2.4242424242424248, 3.636363636363637, 4.8484848484848495, 6.060606060606062, 7.272727272727274, 8.484848484848486, 9.696969696969699, 10.909090909090912, 12.121212121212125, 13.333333333333336, 14.545454545454549, 15.757575757575761, 16.969696969696972, 18.181818181818187, 19.393939393939398, 20.60606060606061, 21.818181818181824, 23.030303030303035, 24.24242424242425, 25.45454545454546, 26.66666666666667, 27.878787878787886, 29.090909090909097, 30.303030303030308, 31.515151515151523, 32.727272727272734, 33.939393939393945, 35.151515151515156, 36.363636363636374, 37.575757575757585, 38.787878787878796, 40.00000000000001, 41.21212121212122, 42.424242424242436, 43.63636363636365, 44.84848484848486, 46.06060606060607, 47.27272727272728, 48.4848484848485, 49.69696969696971, 50.90909090909092, 52.12121212121213, 53.33333333333334, 54.545454545454554, 55.75757575757577, 56.96969696969698, 58.181818181818194, 59.393939393939405, 60.606060606060616, 61.818181818181834, 63.030303030303045, 64.24242424242425, 65.45454545454547, 66.66666666666669, 67.87878787878789, 69.09090909090911, 70.30303030303031, 71.51515151515153, 72.72727272727275, 73.93939393939395, 75.15151515151517, 76.36363636363637, 77.57575757575759, 78.78787878787881, 80.00000000000001, 81.21212121212123, 82.42424242424244, 83.63636363636365, 84.84848484848487, 86.06060606060608, 87.2727272727273, 88.4848484848485, 89.69696969696972, 90.90909090909093, 92.12121212121214, 93.33333333333336, 94.54545454545456, 95.75757575757578, 96.969696969697, 98.1818181818182, 99.39393939393942, 100.60606060606062, 101.81818181818184, 103.03030303030305, 104.24242424242426, 105.45454545454548, 106.66666666666669, 107.8787878787879, 109.09090909090911, 110.30303030303033, 111.51515151515154, 112.72727272727275, 113.93939393939397, 115.15151515151517, 116.36363636363639, 117.5757575757576, 118.78787878787881, 120.00000000000003} |

Physics and variables selection

| **Physics interface** | **Discretization** |
| --- | --- |
| Transport of Diluted Species (tds) | physics |
| Electric Currents (ec) | physics |

Mesh selection

| **Geometry** | **Mesh** |
| --- | --- |
| Geometry 1 (geom1) | mesh1 |

- 1. Solver Configurations
     1. Solution 1

#### Compile Equations: Time Dependent (st1)

Study and step

| **Description** | **Value** |
| --- | --- |
| Use study | [Study 1](#cs5322987) |
| Use study step | [Time Dependent](#cs2636590) |

#### Dependent Variables 1 (v1)

General

| **Description** | **Value** |
| --- | --- |
| Defined by study step | [Time Dependent](#cs2636590) |

Residual scaling

| **Description** | **Value** |
| --- | --- |
| Method | Manual |

Initial value calculation constants

| **Constant name** | **Initial value source** |
| --- | --- |
| t | range(0,(3.6/v-0)/99,3.6/v) |
| timestep | 0.012[s] |

##### Concentration (comp1.cA) (comp1_cA)

General

| **Description** | **Value** |
| --- | --- |
| Field components | comp1.cA |
| Internal variables | {comp1.uflux.cA, comp1.dflux.cA, comp1.tds.dt2Inv_cA} |

##### Concentration (comp1.cB) (comp1_cB)

General

| **Description** | **Value** |
| --- | --- |
| Field components | comp1.cB |
| Internal variables | {comp1.uflux.cB, comp1.dflux.cB, comp1.tds.dt2Inv_cB} |

##### Concentration (comp1.cO) (comp1_cO)

General

| **Description** | **Value** |
| --- | --- |
| Field components | comp1.cO |
| Internal variables | {comp1.uflux.cO, comp1.dflux.cO, comp1.tds.dt2Inv_cO} |

##### Concentration (comp1.cR) (comp1_cR)

General

| **Description** | **Value** |
| --- | --- |
| Field components | comp1.cR |
| Internal variables | {comp1.uflux.cR, comp1.dflux.cR, comp1.tds.dt2Inv_cR} |

#### Time-Dependent Solver 1 (t1)

General

| **Description** | **Value** |
| --- | --- |
| Defined by study step | [Time Dependent](#cs2636590) |
| Output times | {0, 0.12121212121212122, 0.24242424242424243, 0.36363636363636365, 0.48484848484848486, 0.6060606060606061, 0.7272727272727273, 0.8484848484848485, 0.9696969696969697, 1.0909090909090908, 1.2121212121212122, 1.3333333333333335, 1.4545454545454546, 1.5757575757575757, 1.696969696969697, 1.8181818181818183, 1.9393939393939394, 2.0606060606060606, 2.1818181818181817, 2.303030303030303, 2.4242424242424243, 2.5454545454545454, 2.666666666666667, 2.787878787878788, 2.909090909090909, 3.0303030303030303, 3.1515151515151514, 3.272727272727273, 3.393939393939394, 3.515151515151515, 3.6363636363636367, 3.757575757575758, 3.878787878787879, 4, 4.121212121212121, 4.242424242424242, 4.363636363636363, 4.484848484848485, 4.606060606060606, 4.7272727272727275, 4.848484848484849, 4.96969696969697, 5.090909090909091, 5.212121212121212, 5.333333333333334, 5.454545454545455, 5.575757575757576, 5.696969696969697, 5.818181818181818, 5.9393939393939394, 6.0606060606060606, 6.181818181818182, 6.303030303030303, 6.424242424242425, 6.545454545454546, 6.666666666666667, 6.787878787878788, 6.909090909090909, 7.03030303030303, 7.151515151515151, 7.272727272727273, 7.3939393939393945, 7.515151515151516, 7.636363636363637, 7.757575757575758, 7.878787878787879, 8, 8.121212121212121, 8.242424242424242, 8.363636363636363, 8.484848484848484, 8.606060606060606, 8.727272727272727, 8.84848484848485, 8.96969696969697, 9.090909090909092, 9.212121212121213, 9.333333333333334, 9.454545454545455, 9.575757575757576, 9.696969696969697, 9.818181818181818, 9.93939393939394, 10.06060606060606, 10.181818181818182, 10.303030303030303, 10.424242424242424, 10.545454545454545, 10.666666666666668, 10.787878787878789, 10.90909090909091, 11.030303030303031, 11.151515151515152, 11.272727272727273, 11.393939393939394, 11.515151515151516, 11.636363636363637, 11.757575757575758, 11.878787878787879, 12} |
| Relative tolerance | 0.005 |

Time stepping

| **Description** | **Value** |
| --- | --- |
| Maximum BDF order | 2 |
| Nonlinear controller | On |

##### Advanced (aDef)

Assembly settings

| **Description** | **Value** |
| --- | --- |
| Reuse sparsity pattern | On |

##### Fully Coupled 1 (fc1)

General

| **Description** | **Value** |
| --- | --- |
| Linear solver | [Direct, concentrations (tds)](#cs5401109) |

Method and termination

| **Description** | **Value** |
| --- | --- |
| Damping factor | 0.9 |
| Jacobian update | Once per time step |
| Maximum number of iterations | 8 |
| Stabilization and acceleration | Anderson acceleration |
| Dimension of iteration space | 5 |
| Mixing parameter | 0.9 |
| Iteration delay | 1 |

##### Direct, concentrations (tds) (d1)

General

| **Description** | **Value** |
| --- | --- |
| Solver | PARDISO |
| Pivoting perturbation | 1.0E-13 |

1. Results
   1. Data Sets
      1. Study 1/Solution 1

Solution

| **Description** | **Value** |
| --- | --- |
| Solution | [Solution 1](#cs3286207) |
| Component | Component 1 (comp1) |


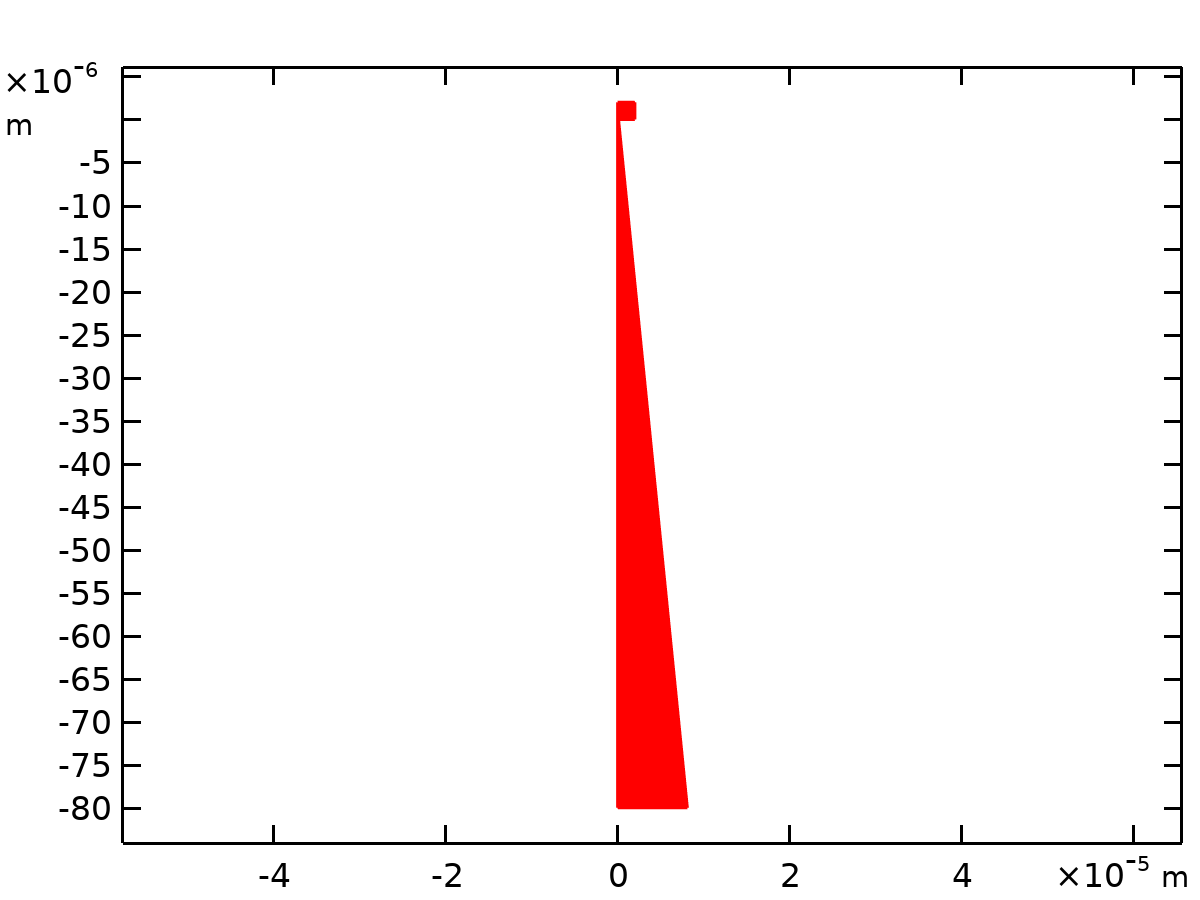


Dataset: Study 1/Solution 1

- - 1. Revolution 2D 1

Data

| **Description** | **Value** |
| --- | --- |
| Dataset | [Study 1/Solution 1](#cs9538770) |

Axis data

| **Description** | **Value** |
| --- | --- |
| Axis entry method | Two points |
| Points | {{0, 0}, {0, 1}} |

Revolution layers

| **Description** | **Value** |
| --- | --- |
| Start angle | -90 |
| Revolution angle | 225 |


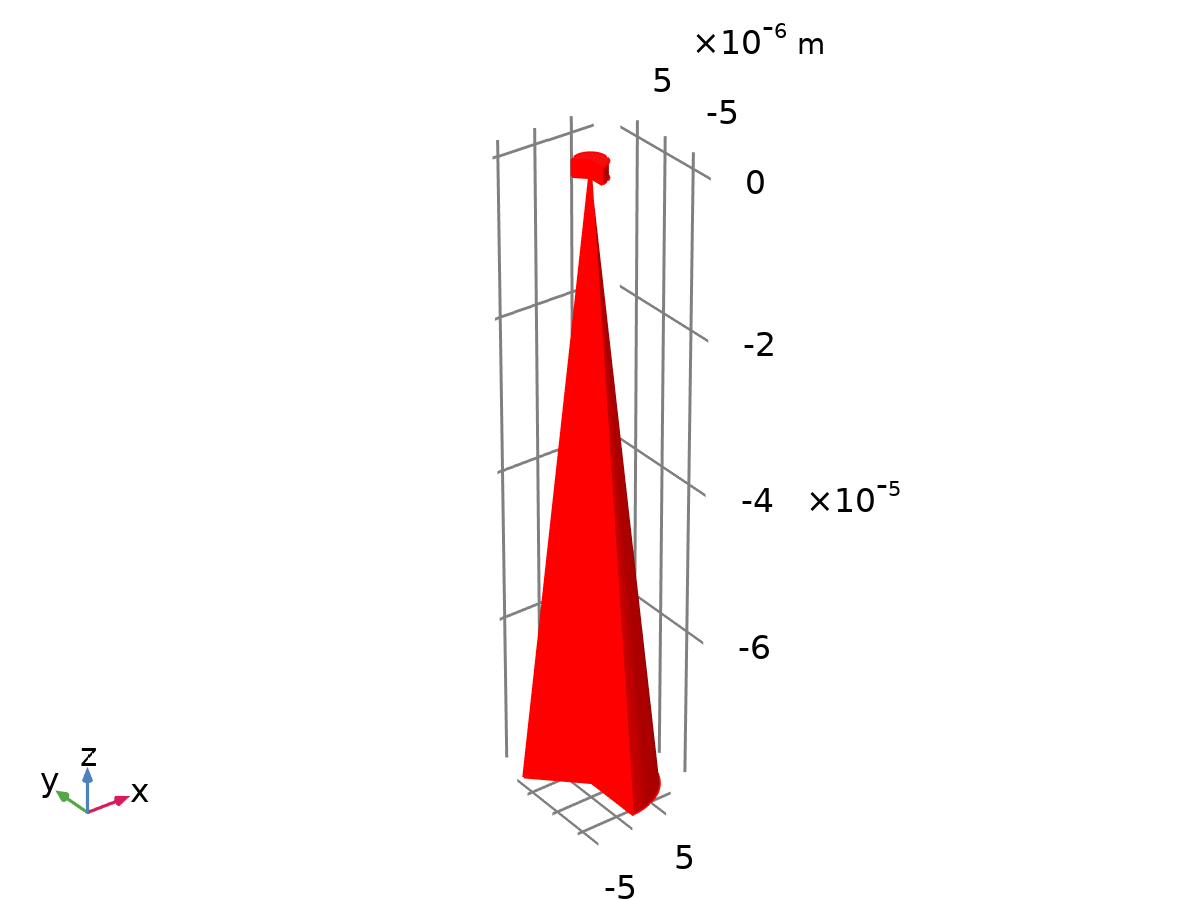


Dataset: Revolution 2D 1

- 1. Derived Values
     1. Line Integration 1

Data

| **Description** | **Value** |
| --- | --- |
| Dataset | [Study 1/Solution 1](#cs9538770) |

Expressions

| **Expression** | **Unit** | **Description** |
| --- | --- | --- |
| tds.ntflux_cR*F | A |  |

Integration settings

| **Description** | **Value** |
| --- | --- |
| Integration order | 4 |
| Compute surface integral | On |

- - 1. Line Average 1

Data

| **Description** | **Value** |
| --- | --- |
| Dataset | [Study 1/Solution 1](#cs9538770) |

Expressions

| **Expression** | **Unit** | **Description** |
| --- | --- | --- |
| E | V |  |

Integration settings

| **Description** | **Value** |
| --- | --- |
| Integration order | 4 |
| Compute surface integral | On |

- 1. Plot Groups
     1. Concentration, O (tds)


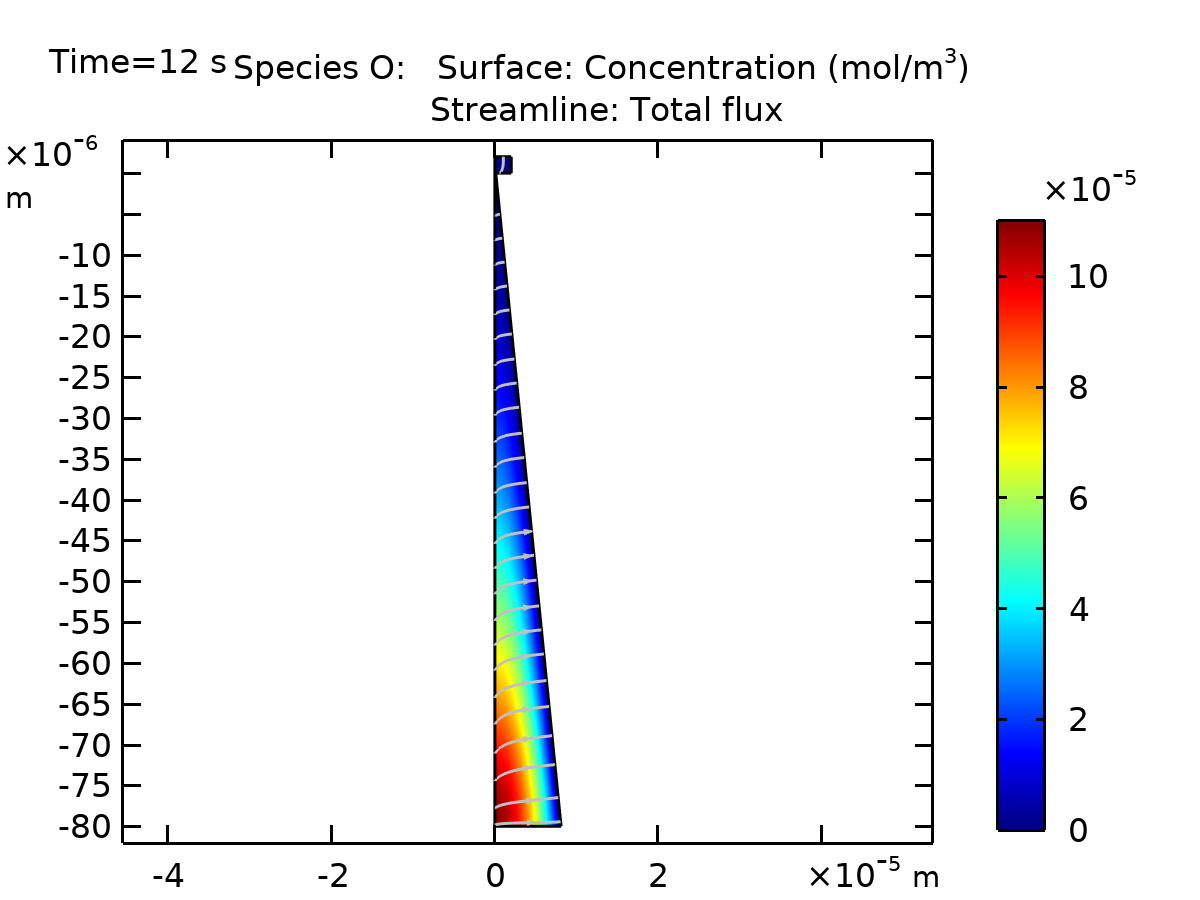


Species O: Surface: Concentration (mol/m^3^) Streamline: Total flux

- - 1. Concentration, O, 3D (tds)


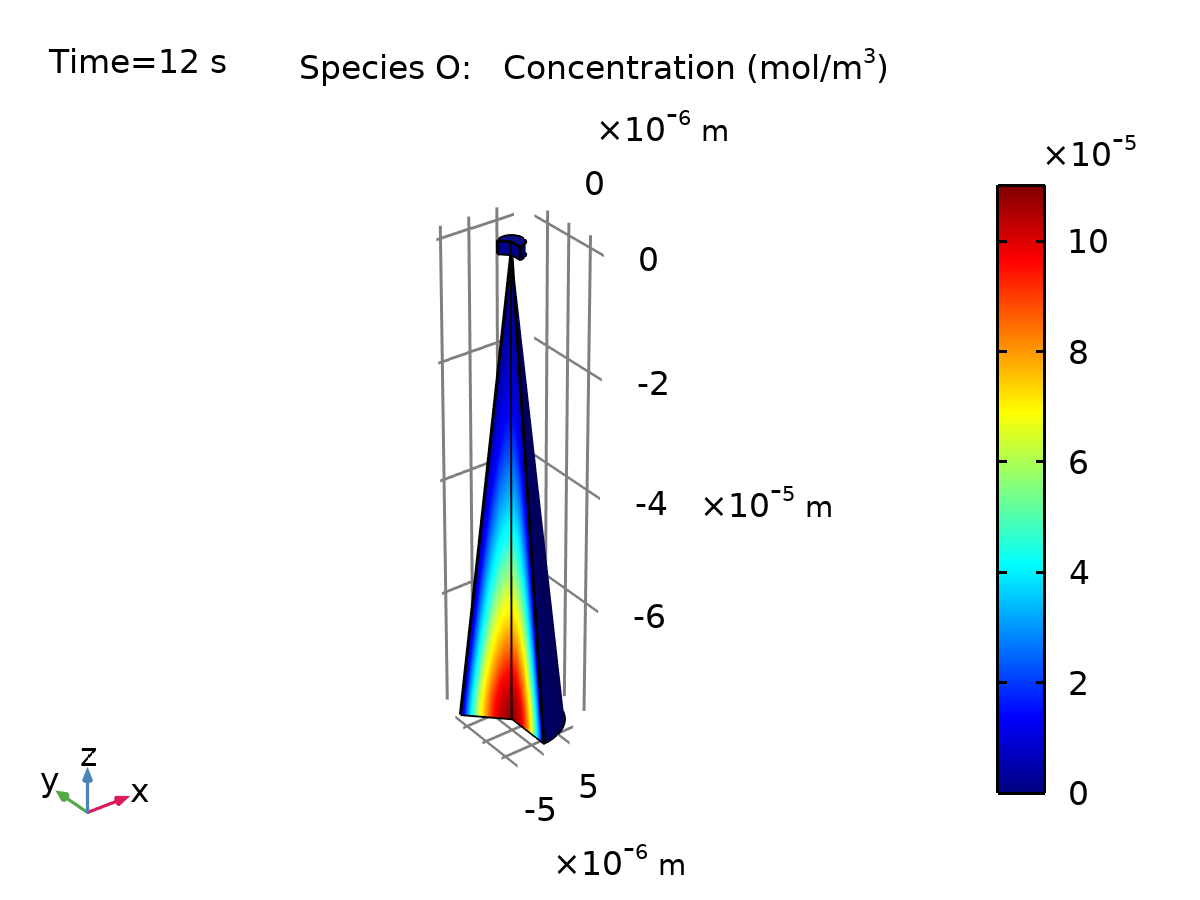


Species O: Concentration (mol/m^3^)

- - 1. Concentration, R (tds)


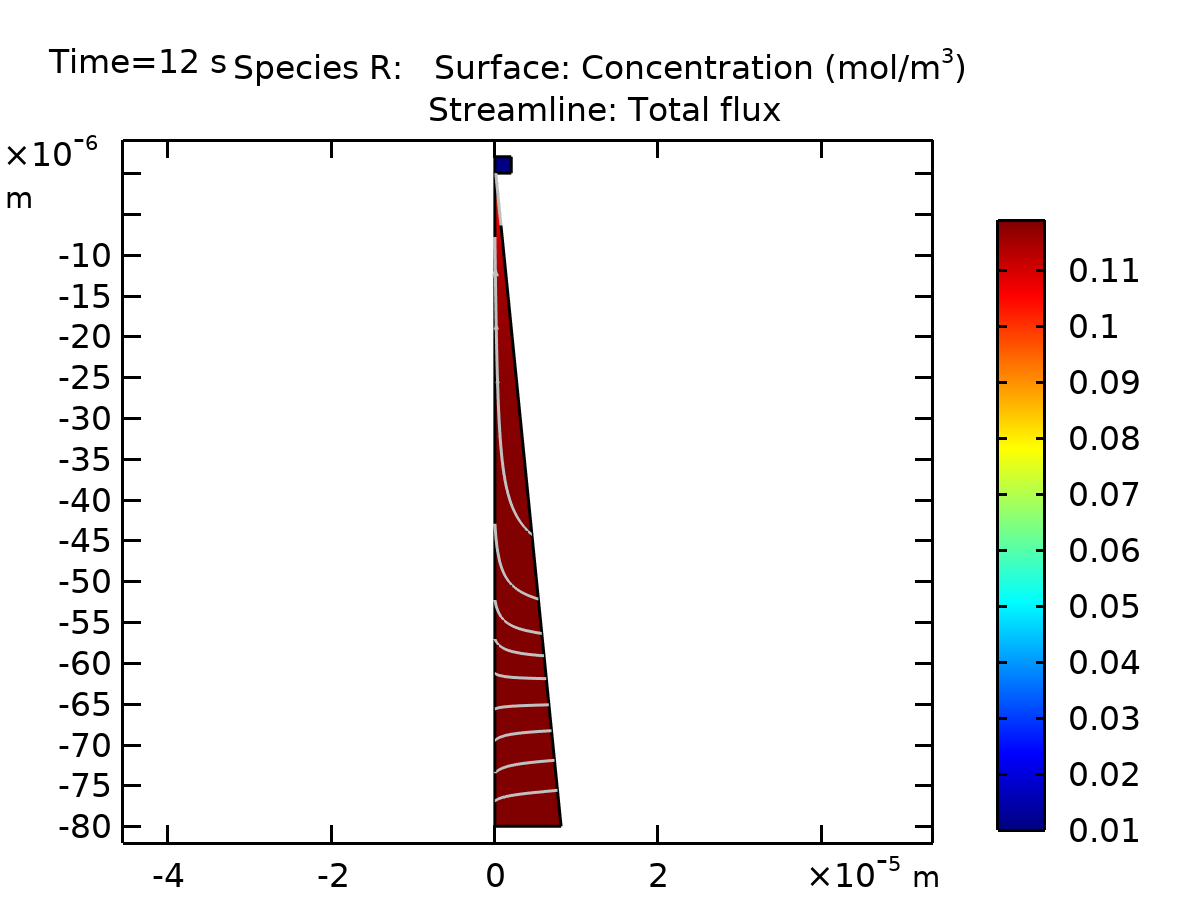


Species R: Surface: Concentration (mol/m^3^) Streamline: Total flux

- - 1. Concentration, R, 3D (tds)


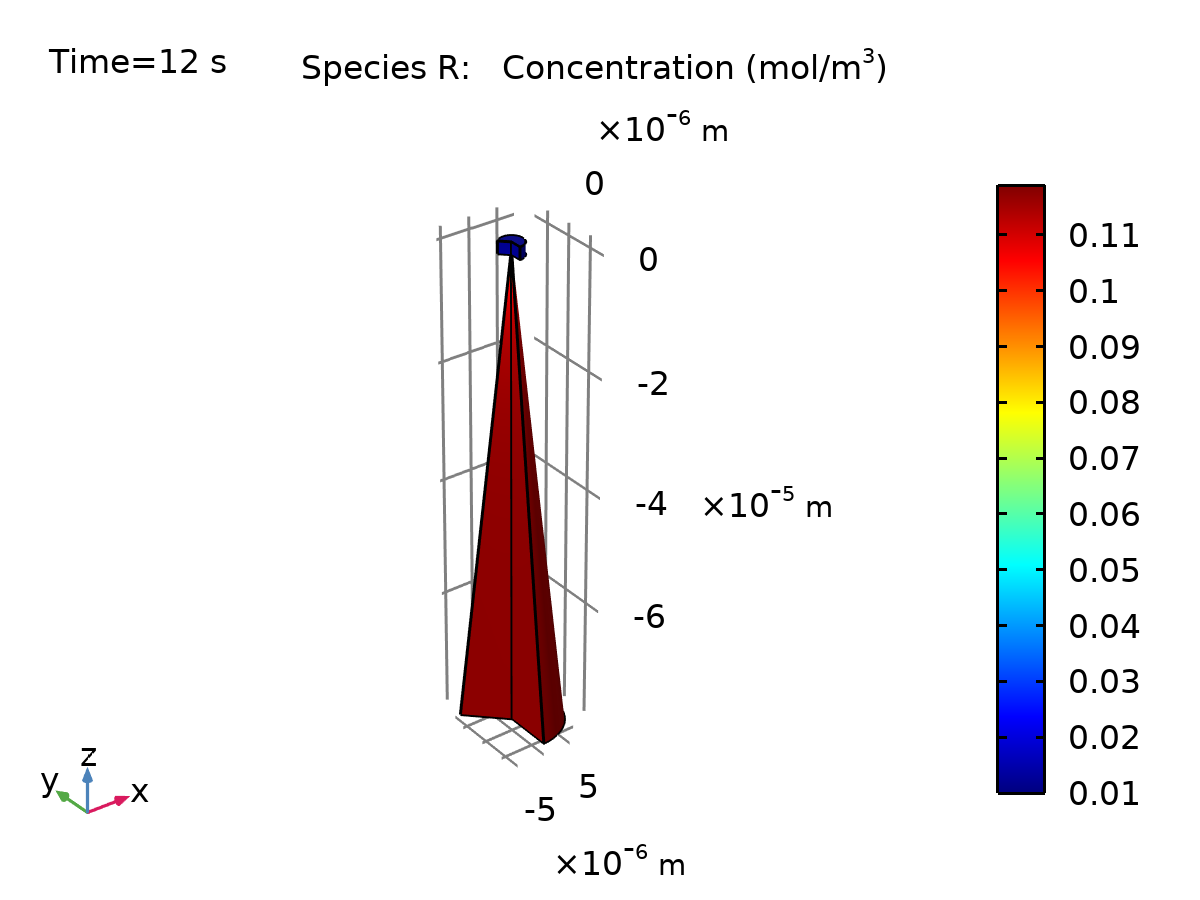


Species R: Concentration (mol/m^3^)

- - 1. Concentration, A (tds)


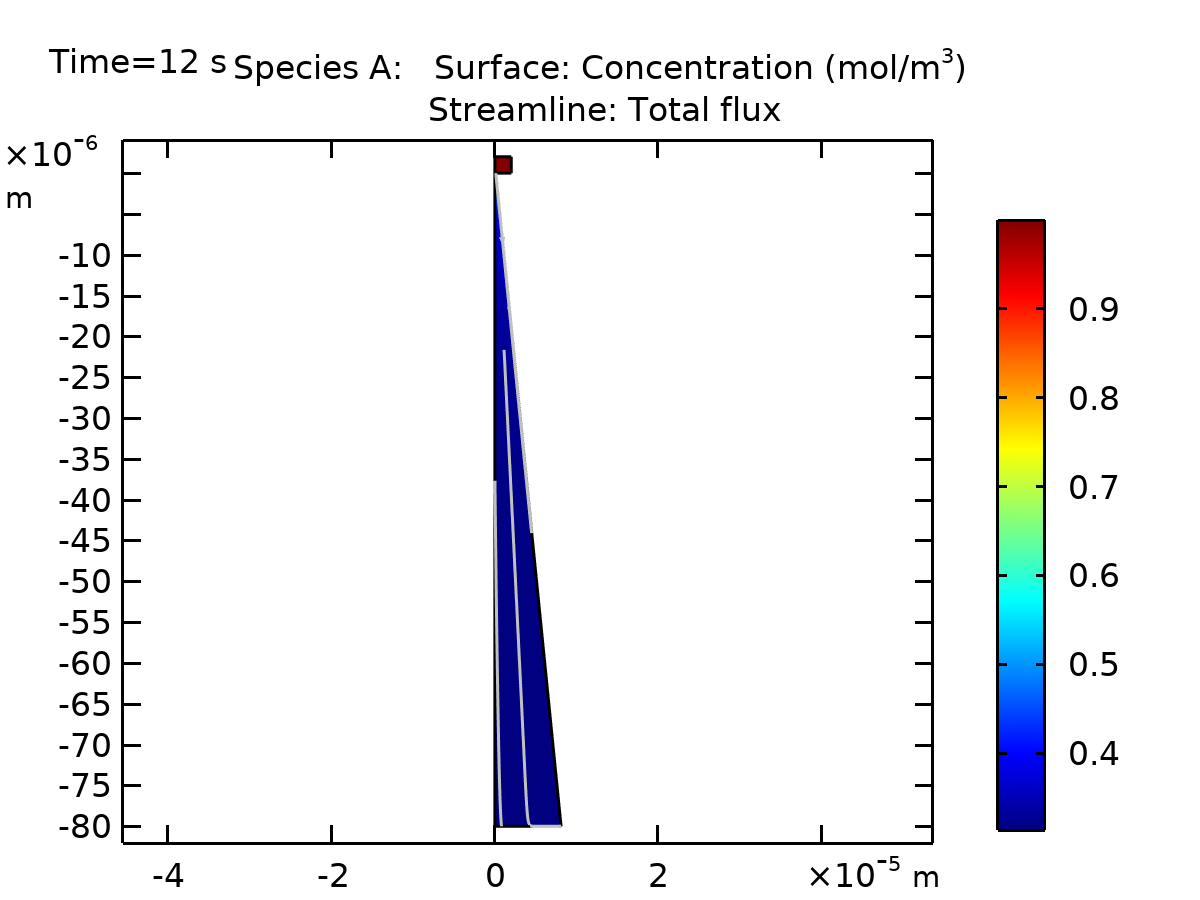


Species A: Surface: Concentration (mol/m^3^) Streamline: Total flux

- - 1. Concentration, A, 3D (tds)


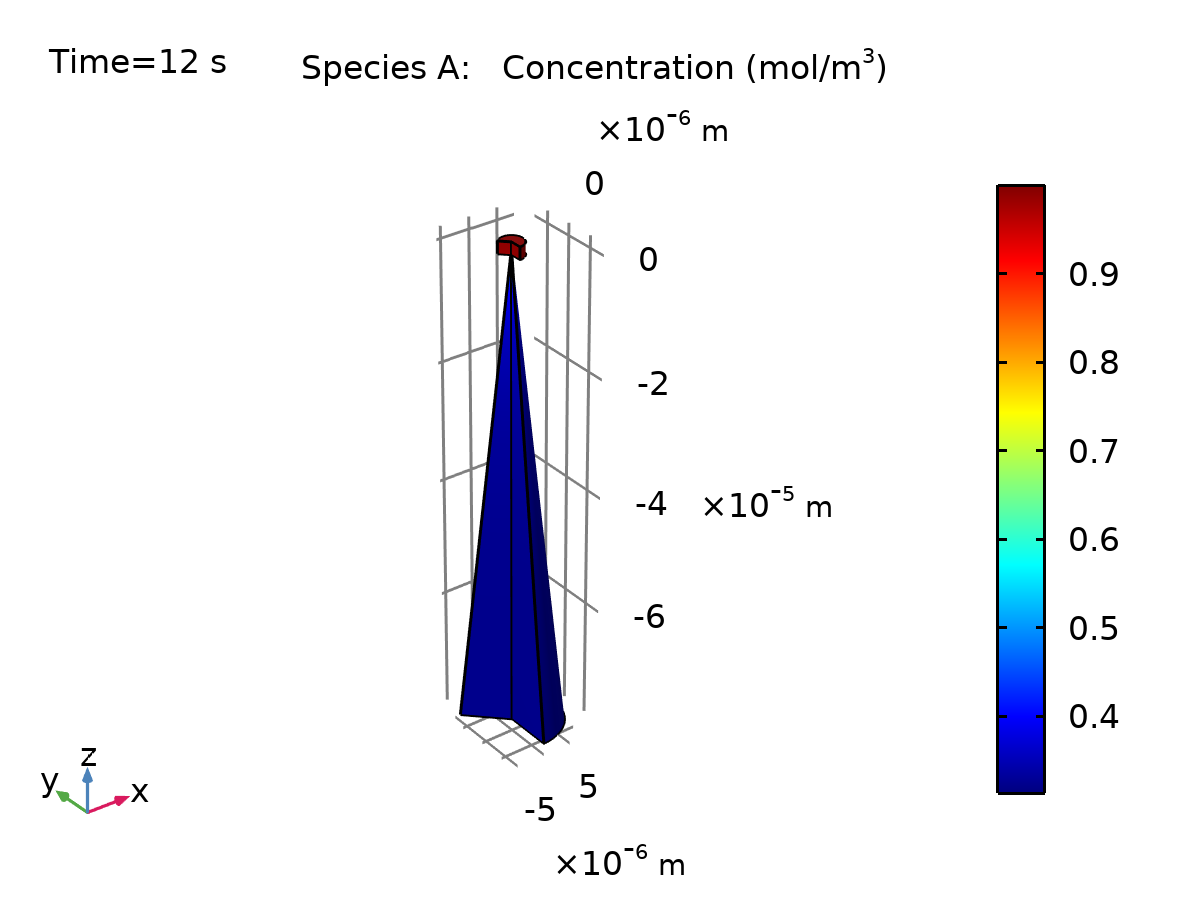


Species A: Concentration (mol/m^3^)

[
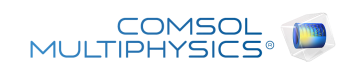
](https://www.comsol.com/)

Carbon nanopipettes-depth

| Report date | Sep 9, 2025 6:54:39 PM |
| --- | --- |

Contents

[5 Global Definitions 46](#_Toc210479038)

[5.1 Parameters 46](#_Toc210479039)

[5.2 Variables 46](#_Toc210479040)

[5.3 Functions 47](#_Toc210479041)

[6 Component 1 48](#_Toc210479042)

[6.1 Definitions 48](#_Toc210479043)

[6.2 Geometry 1 49](#_Toc210479044)

[6.3 Transport of Diluted Species 50](#_Toc210479045)

[6.4 Mesh 1 63](#_Toc210479046)

[7 Study 2 68](#_Toc210479047)

[7.1 Parametric Sweep 68](#_Toc210479048)

[7.2 Time Dependent 68](#_Toc210479049)

[8 Results 69](#_Toc210479050)

[8.1 Data Sets 69](#_Toc210479051)

[8.2 Plot Groups 71](#_Toc210479052)

1. Global Definitions

| Date | Jan 14, 2025 8:55:59 AM |
| --- | --- |

Global settings

| Name | Carbon nanopipettes-depth.mph |
| --- | --- |
| Path | D:\Carbon nanopipettes-depth.mph |
| Version | COMSOL Multiphysics 5.6 (Build: 280) |
| Unit system | SI |

Used products

| COMSOL Multiphysics |
| --- |
| Chemical Reaction Engineering Module |

Computer information

| CPU | Intel64 Family 6 Model 170 Stepping 4, 22 cores |
| --- | --- |
| Operating system | Windows 10 |

- 1. Parameters

Parameters 1

| **Name** | **Expression** | **Value** | **Description** |
| --- | --- | --- | --- |
| c0 | 1 [mol/m^3] | 1 mol/m³ |  |
| ad | 80 | 80 |  |
| c1 | 1[mol/m^3] | 1 mol/m³ |  |
| k0 | 10[cm/s] | 0.1 m/s |  |
| E0 | 0[V] | 0 V |  |
| Vapp_0 | 0.1[V] | 0.1 V |  |
| F | 96500[C/mol] | 96500 C/mol |  |
| depth | 1[um] | 1E−6 m |  |

- 1. Variables
     1. Variables 1a

Selection

| Geometric entity level | Entire model |
| --- | --- |

| **Name** | **Expression** | **Unit** | **Description** |
| --- | --- | --- | --- |
| Vapp | 1[V]*wv1(t/1[s]) | V |  |

- 1. Functions
     1. Waveform 1

| Function name | wv1 |
| --- | --- |
| Function type | Waveform |


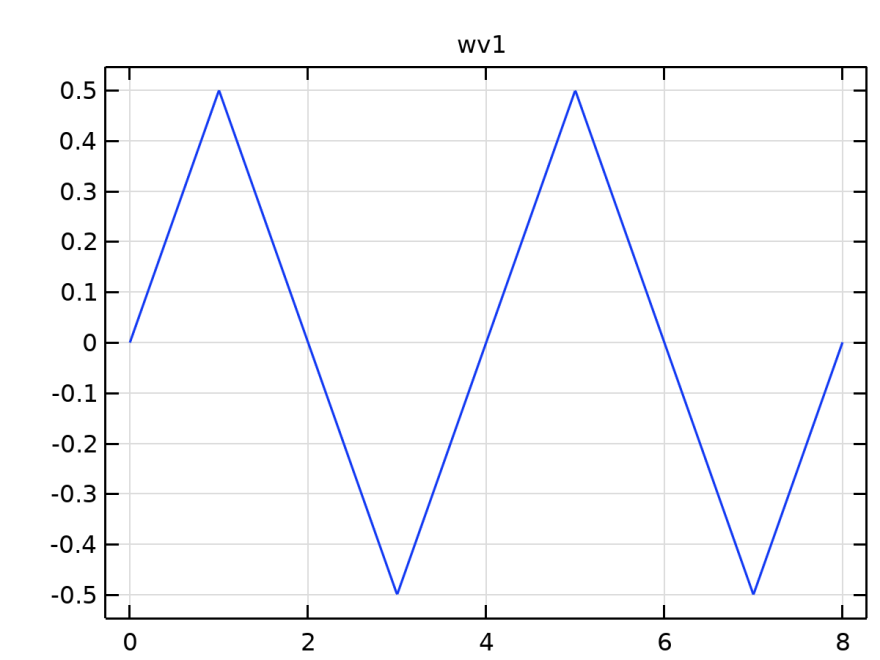


Waveform 1

Parameters

| **Description** | **Value** |
| --- | --- |
| Type | Triangle |
| Size of transition zone | 0.0001 |
| Angular frequency | pi*0.5 |
| Amplitude | 0.5 |

1. Component 1

| Date | Dec 11, 2015 12:21:46 AM |
| --- | --- |

Settings

| **Description** | **Value** |
| --- | --- |
| Unit system | Same as global system (SI) |
| Geometry shape function | Automatic |
| Avoid inverted elements by curving interior domain elements | Off |

Spatial frame coordinates

| **First** | **Second** | **Third** |
| --- | --- | --- |
| r | phi | z |

Material frame coordinates

| **First** | **Second** | **Third** |
| --- | --- | --- |
| R | PHI | Z |

Geometry frame coordinates

| **First** | **Second** | **Third** |
| --- | --- | --- |
| Rg | PHIg | Zg |

Mesh frame coordinates

| **First** | **Second** | **Third** |
| --- | --- | --- |
| Rm | PHIm | Zm |

- 1. Definitions
     1. Coordinate Systems

#### Boundary System 1

| Coordinate system type | Boundary system |
| --- | --- |
| Tag | sys1 |

Coordinate names

| **First** | **Second** | **Third** |
| --- | --- | --- |
| t1 | to | n |

- 1. Geometry 1


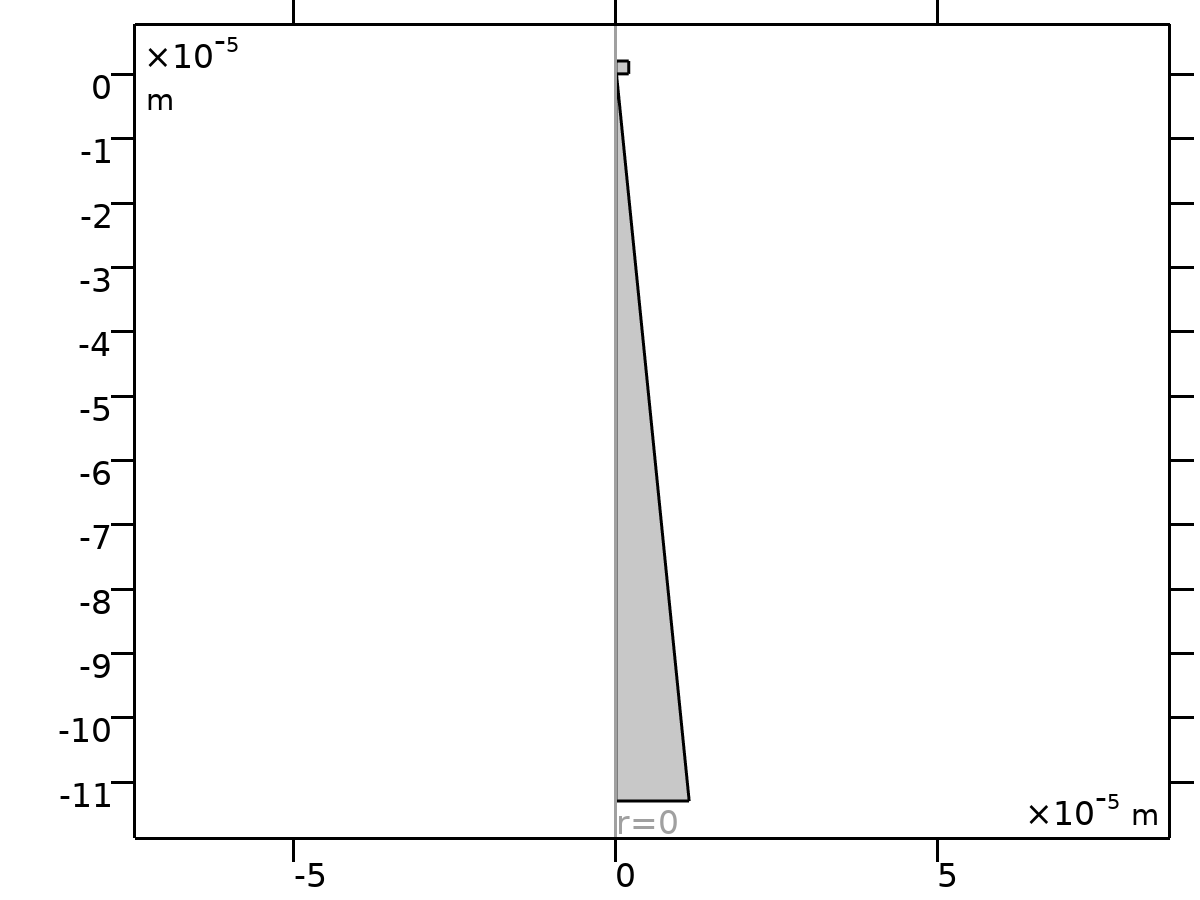


Geometry 1

Units

| Length unit | m |
| --- | --- |
| Angular unit | deg |

Geometry statistics

| **Description** | **Value** |
| --- | --- |
| Space dimension | 2 |
| Number of domains | 1 |
| Number of boundaries | 8 |
| Number of vertices | 8 |

- - 1. Point 1 (pt1)

Point

| **Description** | **Value** |
| --- | --- |
| Point coordinate | {1.1E-7, 0} |

- - 1. Bézier Polygon 1 (b1)

Polygon segments

| **Description** | **Value** |
| --- | --- |
| Control points | {{0, 2e-6, 2e-6, 100e-9, 11.4e-6, 0, 0}, {2e-6, 2e-6, 0, 0, -113e-6, -113e-6, 2e-6}} |
| Degree | {1, 1, 1, 1, 1, 1} |
| Weights | {1, 1, 1, 1, 1, 1, 1, 1, 1, 1, 1, 1} |
| Type | Solid |

- - 1. Point 2 (pt2)

Point

| **Description** | **Value** |
| --- | --- |
| Point coordinate | {2.0E-7, -1.0E-6} |

- 1. Transport of Diluted Species

Used products

| COMSOL Multiphysics |
| --- |
| Chemical Reaction Engineering Module |


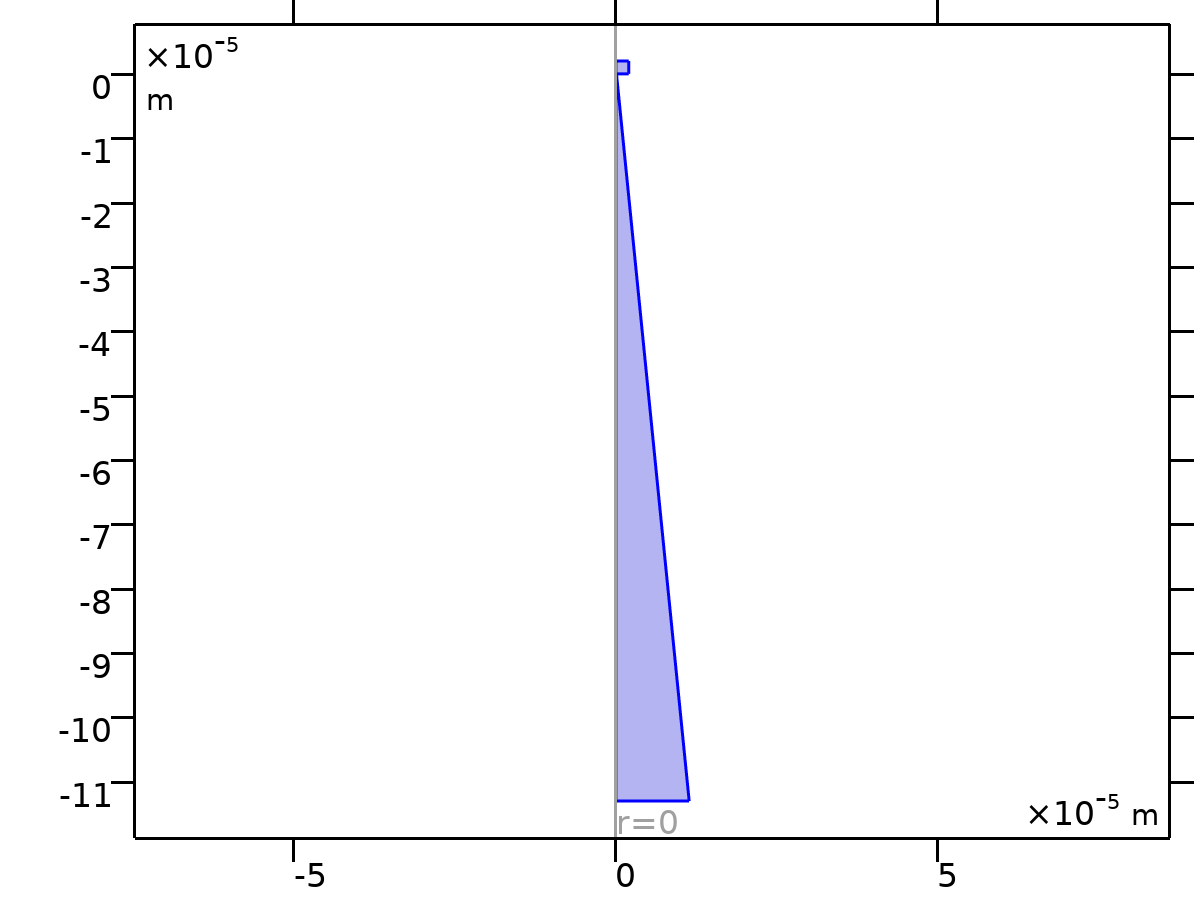


Transport of Diluted Species

Selection

| Geometric entity level | Domain |
| --- | --- |
| Selection | Geometry geom1: Dimension 2: Domain 1 |

Equations


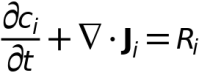


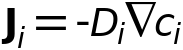


- - 1. Interface Settings

#### Discretization

Settings

| **Description** | **Value** |
| --- | --- |
| Concentration | Linear |

#### Transport Mechanisms

Settings

| **Description** | **Value** |
| --- | --- |
| Convection | Off |
| Migration in electric field | Off |
| Mass transfer in porous media | Off |

- - 1. Variables

| **Name** | **Expression** | **Unit** | **Description** | **Selection** | **Details** |
| --- | --- | --- | --- | --- | --- |
| chds.R_cO | 0 | mol/(m³·s) | Total rate expression | Domain 1 | + operation |
| chds.cP_cO | 0 | mol/kg | Concentration species absorbed to the solid | Domain 1 | + operation |
| chds.cP_cO | 0 | mol/kg | Concentration species absorbed to the solid | Boundaries 1–8 | + operation |
| chds.KP_cO | 0 | m³/kg | Adsorption isotherm, first concentration derivative | Domain 1 | + operation |
| chds.KP_cO | 0 | m³/kg | Adsorption isotherm, first concentration derivative | Boundaries 1–8 | + operation |
| chds.R_cR | 0 | mol/(m³·s) | Total rate expression | Domain 1 | + operation |
| chds.cP_cR | 0 | mol/kg | Concentration species absorbed to the solid | Domain 1 | + operation |
| chds.cP_cR | 0 | mol/kg | Concentration species absorbed to the solid | Boundaries 1–8 | + operation |
| chds.KP_cR | 0 | m³/kg | Adsorption isotherm, first concentration derivative | Domain 1 | + operation |
| chds.KP_cR | 0 | m³/kg | Adsorption isotherm, first concentration derivative | Boundaries 1–8 | + operation |
| chds.poro | 1 | 1 | Porosity | Domain 1 |  |
| chds.theta_g | 0 | 1 | Gas volume fraction | Domain 1 |  |
| chds.theta | chds.poro | 1 | Mobile fluid volume fraction | Domain 1 |  |
| chds.nr | dnr | 1 | Normal vector, r component | Boundaries 1–8 |  |
| chds.nphi | 0 | 1 | Normal vector, phi component | Boundaries 1–8 |  |
| chds.nz | dnz | 1 | Normal vector, z component | Boundaries 1–8 |  |
| chds.nrmesh | dnrmesh | 1 | Normal vector (mesh), r component | Boundaries 1–8 |  |
| chds.nphimesh | 0 | 1 | Normal vector (mesh), phi component | Boundaries 1–8 |  |
| chds.nzmesh | dnzmesh | 1 | Normal vector (mesh), z component | Boundaries 1–8 |  |
| chds.nrc | root.nrc/chds.ncLen | 1 | Normal vector, r component | Boundaries 1–8 |  |
| chds.nphic | 0 | 1 | Normal vector, phi component | Boundaries 1–8 |  |
| chds.nzc | root.nzc/chds.ncLen | 1 | Normal vector, z component | Boundaries 1–8 |  |
| chds.ncLen | sqrt(root.nrc^2+root.nzc^2+eps) | 1 | Help variable | Boundaries 1–8 |  |

- - 1. Diffusion and Migration


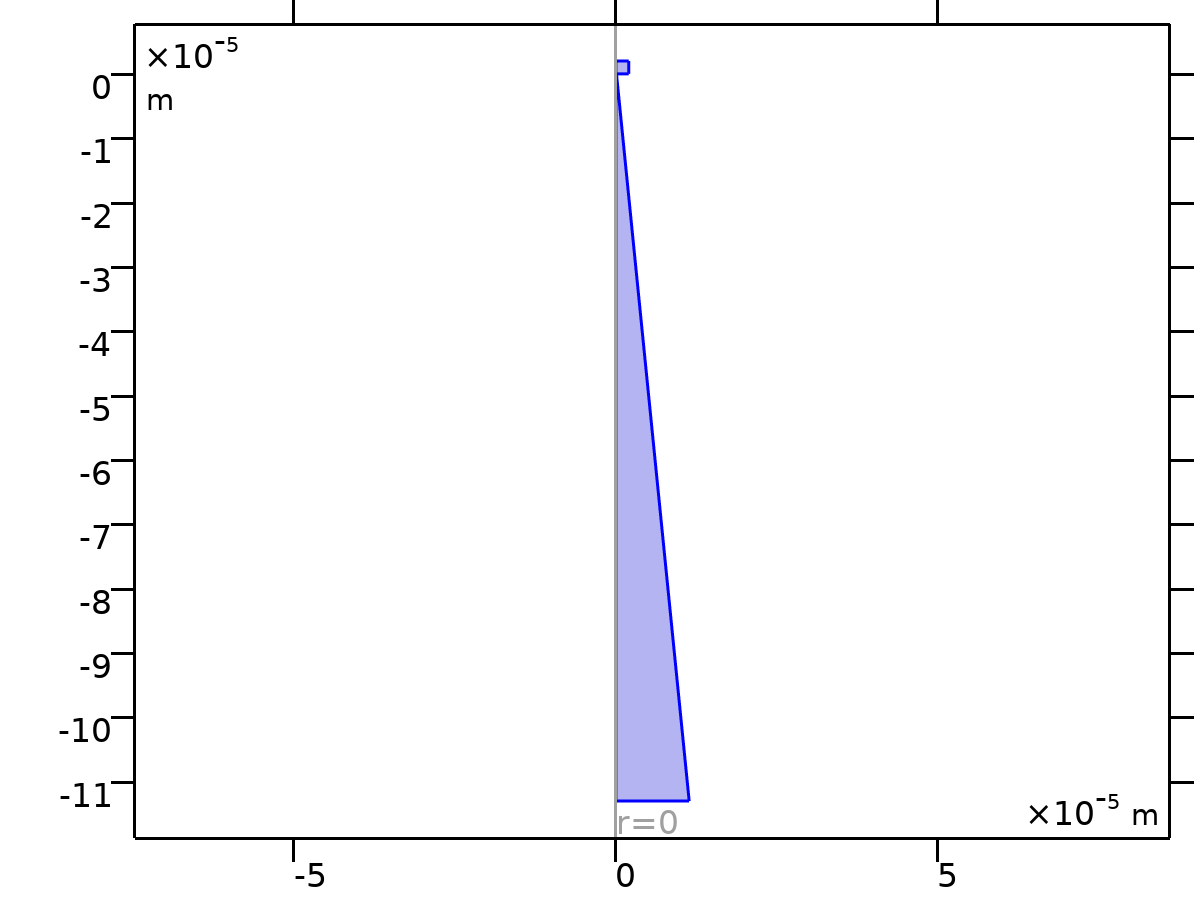


Diffusion and Migration

Selection

| Geometric entity level | Domain |
| --- | --- |
| Selection | Geometry geom1: Dimension 2: All domains |

Equations


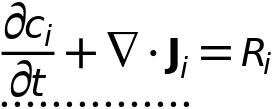


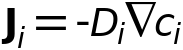


#### Diffusion

Settings

| **Description** | **Value** |
| --- | --- |
| Source | Material |
| Material | None |
| Diffusion coefficient | User defined |
| Diffusion coefficient | {{1e-9[m^2/s], 0, 0}, {0, 1e-9[m^2/s], 0}, {0, 0, 1e-9[m^2/s]}} |
| Diffusion coefficient | User defined |
| Diffusion coefficient | {{1e-9[m^2/s], 0, 0}, {0, 1e-9[m^2/s], 0}, {0, 0, 1e-9[m^2/s]}} |

#### Coordinate System Selection

Settings

| **Description** | **Value** |
| --- | --- |
| Coordinate system | Global coordinate system |

#### Model Input

Settings

| **Description** | **Value** |
| --- | --- |
| Temperature | User defined |
| Temperature | 293.15[K] |

#### Variables

| **Name** | **Expression** | **Unit** | **Description** | **Selection** | **Details** |
| --- | --- | --- | --- | --- | --- |
| domflux.cOr | 2*chds.dflux_cOr*pi*r | mol/(m·s) | Domain flux, r component | Domain 1 |  |
| domflux.cOz | 2*chds.dflux_cOz*pi*r | mol/(m·s) | Domain flux, z component | Domain 1 |  |
| domflux.cRr | 2*chds.dflux_cRr*pi*r | mol/(m·s) | Domain flux, r component | Domain 1 |  |
| domflux.cRz | 2*chds.dflux_cRz*pi*r | mol/(m·s) | Domain flux, z component | Domain 1 |  |
| chds.ndflux_cO | chds.bndFlux_cO | mol/(m²·s) | Normal diffusive flux | Boundaries 2–8 |  |
| chds.ntflux_cO | chds.bndFlux_cO | mol/(m²·s) | Normal total flux | Boundaries 2–8 |  |
| chds.ndflux_cR | chds.bndFlux_cR | mol/(m²·s) | Normal diffusive flux | Boundaries 2–8 |  |
| chds.ntflux_cR | chds.bndFlux_cR | mol/(m²·s) | Normal total flux | Boundaries 2–8 |  |
| chds.bndFlux_cO | if(r>0.001/sqrt(sqrt(mean(emetric2))),-0.5*dflux_spatial(cO)/(pi*r),NaN) | mol/(m²·s) | Boundary flux | Boundaries 1–8 |  |
| chds.bndFlux_cR | if(r>0.001/sqrt(sqrt(mean(emetric2))),-0.5*dflux_spatial(cR)/(pi*r),NaN) | mol/(m²·s) | Boundary flux | Boundaries 1–8 |  |
| chds.D_cOrr | 1.0E-9[m^2/s] | m²/s | Diffusion coefficient, rr component | Domain 1 |  |
| chds.D_cOphir | 0 | m²/s | Diffusion coefficient, phir component | Domain 1 |  |
| chds.D_cOzr | 0 | m²/s | Diffusion coefficient, zr component | Domain 1 |  |
| chds.D_cOrphi | 0 | m²/s | Diffusion coefficient, rphi component | Domain 1 |  |
| chds.D_cOphiphi | 1.0E-9[m^2/s] | m²/s | Diffusion coefficient, phiphi component | Domain 1 |  |
| chds.D_cOzphi | 0 | m²/s | Diffusion coefficient, zphi component | Domain 1 |  |
| chds.D_cOrz | 0 | m²/s | Diffusion coefficient, rz component | Domain 1 |  |
| chds.D_cOphiz | 0 | m²/s | Diffusion coefficient, phiz component | Domain 1 |  |
| chds.D_cOzz | 1.0E-9[m^2/s] | m²/s | Diffusion coefficient, zz component | Domain 1 |  |
| chds.D_cRrr | 1.0E-9[m^2/s] | m²/s | Diffusion coefficient, rr component | Domain 1 |  |
| chds.D_cRphir | 0 | m²/s | Diffusion coefficient, phir component | Domain 1 |  |
| chds.D_cRzr | 0 | m²/s | Diffusion coefficient, zr component | Domain 1 |  |
| chds.D_cRrphi | 0 | m²/s | Diffusion coefficient, rphi component | Domain 1 |  |
| chds.D_cRphiphi | 1.0E-9[m^2/s] | m²/s | Diffusion coefficient, phiphi component | Domain 1 |  |
| chds.D_cRzphi | 0 | m²/s | Diffusion coefficient, zphi component | Domain 1 |  |
| chds.D_cRrz | 0 | m²/s | Diffusion coefficient, rz component | Domain 1 |  |
| chds.D_cRphiz | 0 | m²/s | Diffusion coefficient, phiz component | Domain 1 |  |
| chds.D_cRzz | 1.0E-9[m^2/s] | m²/s | Diffusion coefficient, zz component | Domain 1 |  |
| chds.Dav_cO | 0.5*(chds.D_cOrr+chds.D_cOzz) | m²/s | Average diffusion coefficient | Domain 1 |  |
| chds.Dav_cR | 0.5*(chds.D_cRrr+chds.D_cRzz) | m²/s | Average diffusion coefficient | Domain 1 |  |
| chds.tflux_cOr | chds.dflux_cOr | mol/(m²·s) | Total flux, r component | Domain 1 | + operation |
| chds.tflux_cOphi | chds.dflux_cOphi | mol/(m²·s) | Total flux, phi component | Domain 1 | + operation |
| chds.tflux_cOz | chds.dflux_cOz | mol/(m²·s) | Total flux, z component | Domain 1 | + operation |
| chds.dfluxMag_cO | sqrt(chds.dflux_cOr^2+chds.dflux_cOphi^2+chds.dflux_cOz^2) | mol/(m²·s) | Diffusive flux magnitude | Domain 1 |  |
| chds.tfluxMag_cO | sqrt(chds.tflux_cOr^2+chds.tflux_cOphi^2+chds.tflux_cOz^2) | mol/(m²·s) | Total flux magnitude | Domain 1 |  |
| chds.dpflux_cOr | 0 | mol/(m²·s) | Dispersive flux, r component | Domain 1 |  |
| chds.dpflux_cOphi | 0 | mol/(m²·s) | Dispersive flux, phi component | Domain 1 |  |
| chds.dpflux_cOz | 0 | mol/(m²·s) | Dispersive flux, z component | Domain 1 |  |
| chds.tflux_cRr | chds.dflux_cRr | mol/(m²·s) | Total flux, r component | Domain 1 | + operation |
| chds.tflux_cRphi | chds.dflux_cRphi | mol/(m²·s) | Total flux, phi component | Domain 1 | + operation |
| chds.tflux_cRz | chds.dflux_cRz | mol/(m²·s) | Total flux, z component | Domain 1 | + operation |
| chds.dfluxMag_cR | sqrt(chds.dflux_cRr^2+chds.dflux_cRphi^2+chds.dflux_cRz^2) | mol/(m²·s) | Diffusive flux magnitude | Domain 1 |  |
| chds.tfluxMag_cR | sqrt(chds.tflux_cRr^2+chds.tflux_cRphi^2+chds.tflux_cRz^2) | mol/(m²·s) | Total flux magnitude | Domain 1 |  |
| chds.dpflux_cRr | 0 | mol/(m²·s) | Dispersive flux, r component | Domain 1 |  |
| chds.dpflux_cRphi | 0 | mol/(m²·s) | Dispersive flux, phi component | Domain 1 |  |
| chds.dpflux_cRz | 0 | mol/(m²·s) | Dispersive flux, z component | Domain 1 |  |
| chds.dflux_cOr | -chds.D_cOrr*cOr-chds.D_cOrz*cOz | mol/(m²·s) | Diffusive flux, r component | Domain 1 |  |
| chds.dflux_cOphi | -chds.D_cOphir*cOr-chds.D_cOphiz*cOz | mol/(m²·s) | Diffusive flux, phi component | Domain 1 |  |
| chds.dflux_cOz | -chds.D_cOzr*cOr-chds.D_cOzz*cOz | mol/(m²·s) | Diffusive flux, z component | Domain 1 |  |
| chds.grad_cOr | cOr | mol/m⁴ | Concentration gradient, r component | Domain 1 |  |
| chds.grad_cOphi | 0 | mol/m⁴ | Concentration gradient, phi component | Domain 1 |  |
| chds.grad_cOz | cOz | mol/m⁴ | Concentration gradient, z component | Domain 1 |  |
| chds.dflux_cRr | -chds.D_cRrr*cRr-chds.D_cRrz*cRz | mol/(m²·s) | Diffusive flux, r component | Domain 1 |  |
| chds.dflux_cRphi | -chds.D_cRphir*cRr-chds.D_cRphiz*cRz | mol/(m²·s) | Diffusive flux, phi component | Domain 1 |  |
| chds.dflux_cRz | -chds.D_cRzr*cRr-chds.D_cRzz*cRz | mol/(m²·s) | Diffusive flux, z component | Domain 1 |  |
| chds.grad_cRr | cRr | mol/m⁴ | Concentration gradient, r component | Domain 1 |  |
| chds.grad_cRphi | 0 | mol/m⁴ | Concentration gradient, phi component | Domain 1 |  |
| chds.grad_cRz | cRz | mol/m⁴ | Concentration gradient, z component | Domain 1 |  |
| chds.Res_cO | d(cO,t)-chds.R_cO | mol/(m³·s) | Equation residual | Domain 1 |  |
| chds.Res_cR | d(cR,t)-chds.R_cR | mol/(m³·s) | Equation residual | Domain 1 |  |

#### Shape functions

| **Name** | **Shape function** | **Unit** | **Description** | **Shape frame** | **Selection** |
| --- | --- | --- | --- | --- | --- |
| cO | Lagrange (Linear) | mol/m³ | Concentration | Material | Domain 1 |
| cR | Lagrange (Linear) | mol/m³ | Concentration | Material | Domain 1 |

#### Weak Expressions

| **Weak expression** | **Integration order** | **Integration frame** | **Selection** |
| --- | --- | --- | --- |
| 2*(-cOt*test(cO)+chds.dflux_cOr*test(cOr)+chds.dflux_cOz*test(cOz))*pi*r | 2 | Material | Domain 1 |
| 2*(-cRt*test(cR)+chds.dflux_cRr*test(cRr)+chds.dflux_cRz*test(cRz))*pi*r | 2 | Material | Domain 1 |
| 2*chds.streamline*(isScalingSystemDomain==0)*pi*r | 2 | Material | Domain 1 |
| 2*chds.crosswind*(isScalingSystemDomain==0)*pi*r | 4 | Material | Domain 1 |

- - 1. Axial Symmetry 1


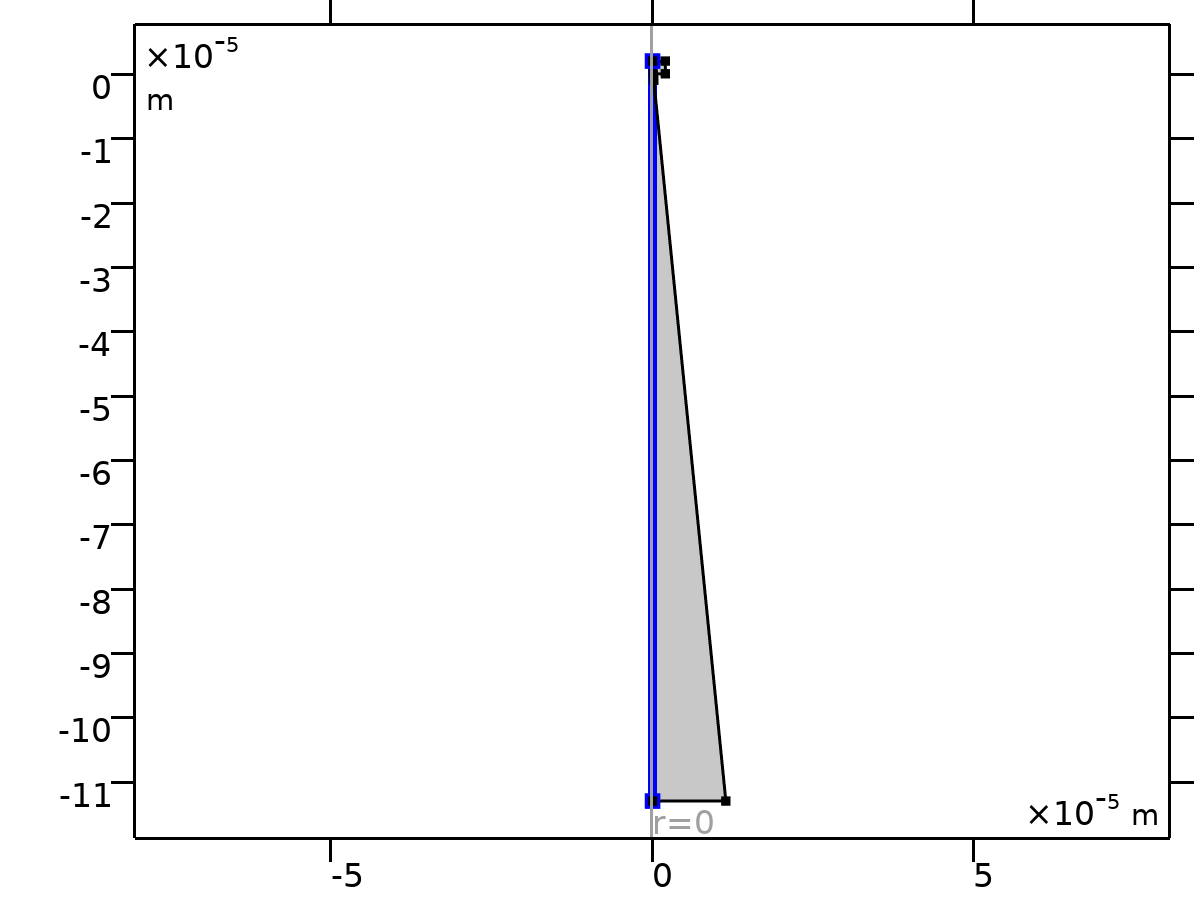


Axial Symmetry 1

Selection

| Geometric entity level | Boundary |
| --- | --- |
| Selection | Geometry geom1: Dimension 1: All boundaries |

- - 1. No Flux 1


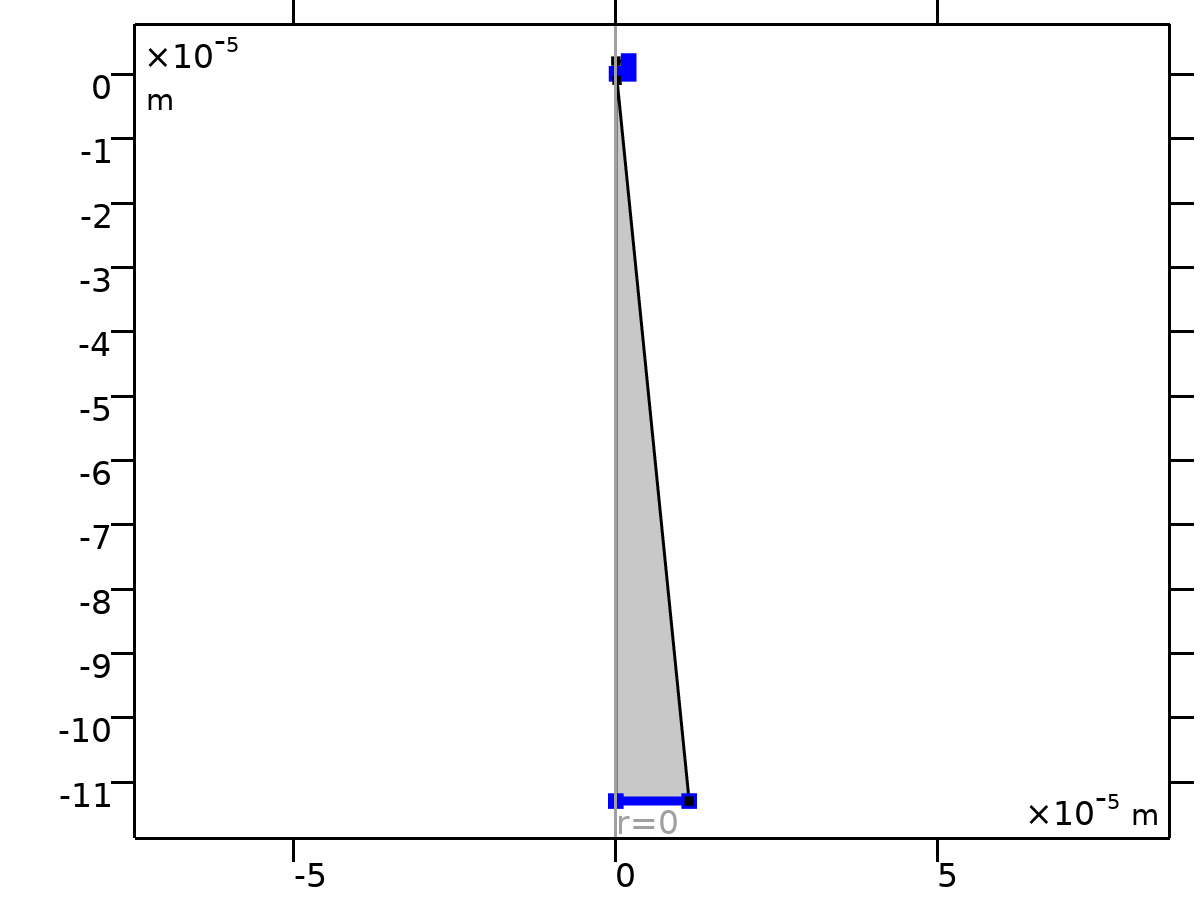


No Flux 1

Selection

| Geometric entity level | Boundary |
| --- | --- |
| Selection | Geometry geom1: Dimension 1: All boundaries |

Equations


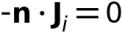


- - 1. Initial Values 1


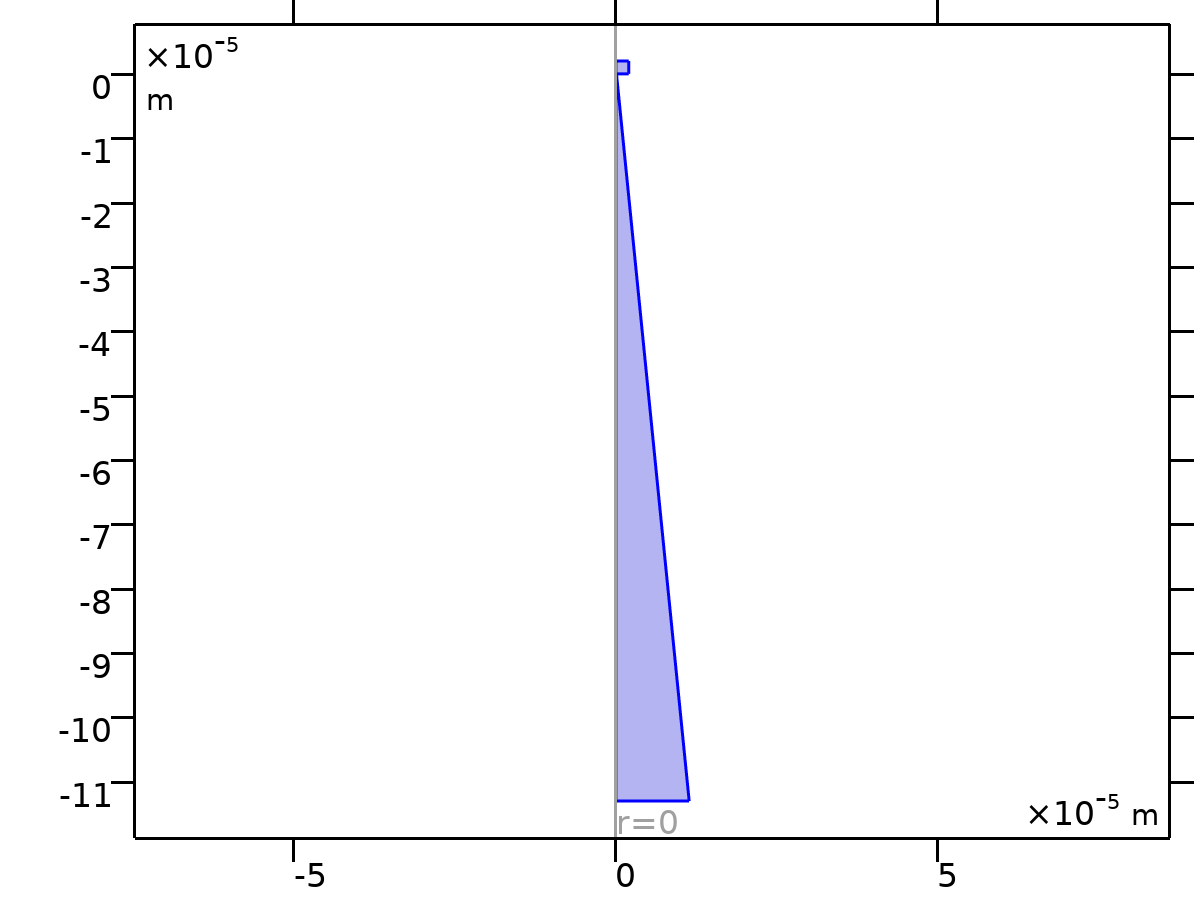


Initial Values 1

Selection

| Geometric entity level | Domain |
| --- | --- |
| Selection | Geometry geom1: Dimension 2: All domains |

#### Initial Values

Settings

| **Description** | **Value** |
| --- | --- |
| Concentration | {c1, c1} |

- - 1. Concentration 1


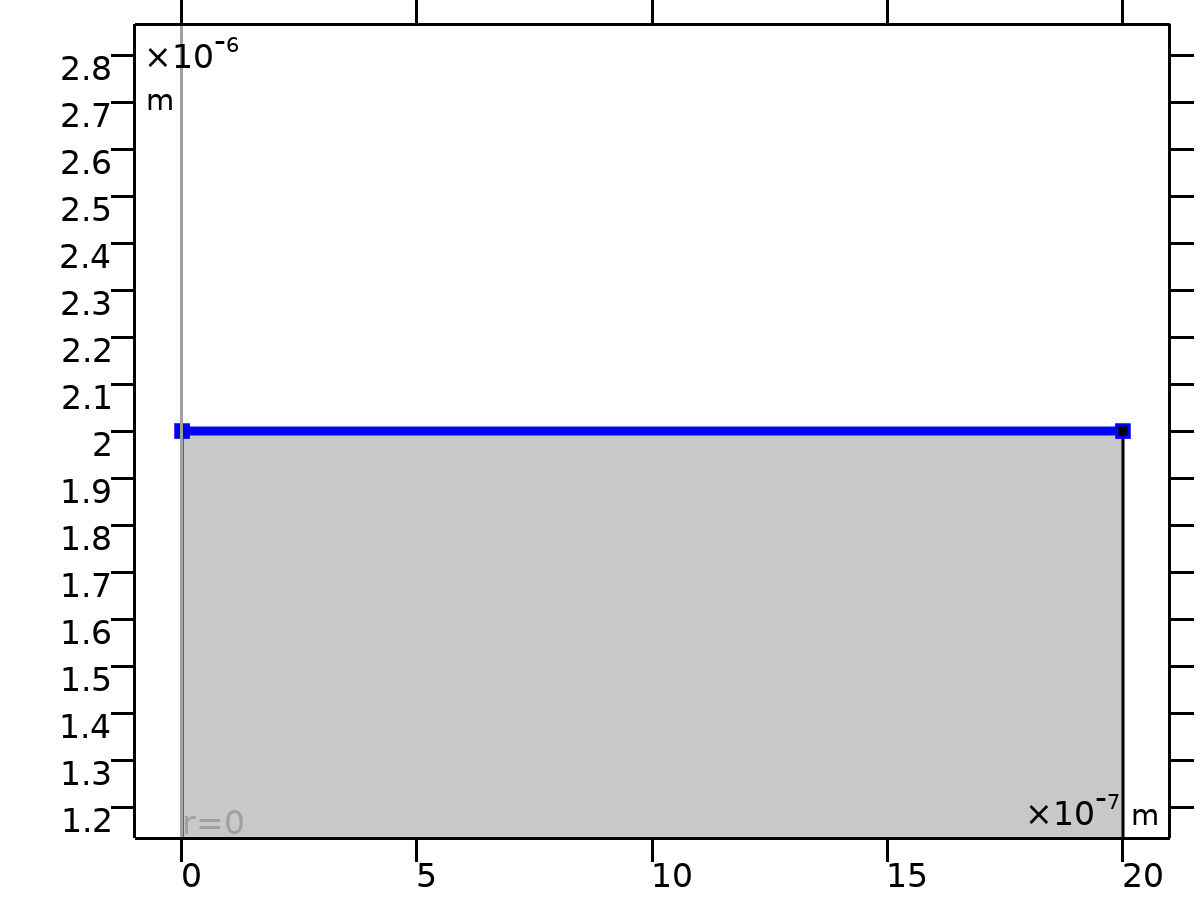


Concentration 1

Selection

| Geometric entity level | Boundary |
| --- | --- |
| Selection | Geometry geom1: Dimension 1: Boundary 3 |

Equations


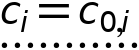


#### Concentration

Settings

| **Description** | **Value** |
| --- | --- |
| Species cO | On |
| Species cR | On |
| Concentration | {c1, c1} |

#### Variables

| **Name** | **Expression** | **Unit** | **Description** | **Selection** |
| --- | --- | --- | --- | --- |
| chds.c0_cO | c1 | mol/m³ | Concentration | Boundary 3 |
| chds.c0_cR | c1 | mol/m³ | Concentration | Boundary 3 |

#### Constraints

| **Constraint** | **Constraint force** | **Shape function** | **Selection** | **Details** |
| --- | --- | --- | --- | --- |
| -cO+chds.c0_cO | test(-cO+chds.c0_cO) | Lagrange (Linear) | Boundary 3 | Elemental |
| -cR+chds.c0_cR | test(-cR+chds.c0_cR) | Lagrange (Linear) | Boundary 3 | Elemental |

- - 1. Flux 1


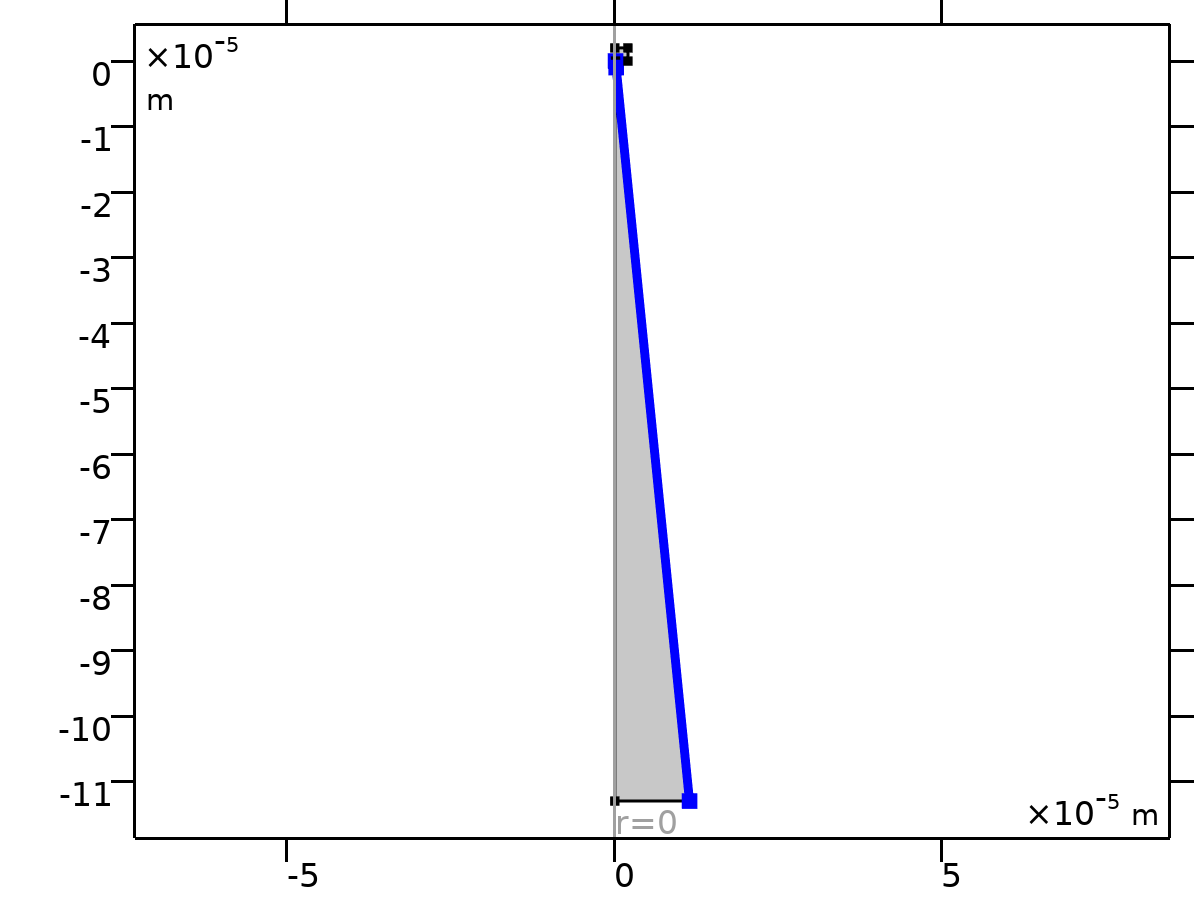


Flux 1

Selection

| Geometric entity level | Boundary |
| --- | --- |
| Selection | Geometry geom1: Dimension 1: Boundaries 4–5, 7 |

Equations


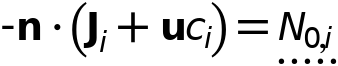


#### Inward Flux

Settings

| **Description** | **Value** |
| --- | --- |
| Flux type | General inward flux |
| Species cO | On |
| Species cR | On |
|  | {-k0*cO*exp(-0.5*38.9/1[V]*(Vapp - E0)) + k0*cR*exp(0.5*38.9/1[V]*(Vapp - E0)), k0*cO*exp(-0.5*38.9/1[V]*(Vapp - E0)) - k0*cR*exp(0.5*38.9/1[V]*(Vapp - E0))} |

#### Weak Expressions

| **Weak expression** | **Integration order** | **Integration frame** | **Selection** |
| --- | --- | --- | --- |
| 2*k0*(-cO*exp(-19.45*(Vapp-E0)/1[V])+cR*exp(19.45*(Vapp-E0)/1[V]))*test(cO)*pi*r | 2 | Material | Boundaries 4–5, 7 |
| 2*k0*(cO*exp(-19.45*(Vapp-E0)/1[V])-cR*exp(19.45*(Vapp-E0)/1[V]))*test(cR)*pi*r | 2 | Material | Boundaries 4–5, 7 |

- 1. Mesh 1


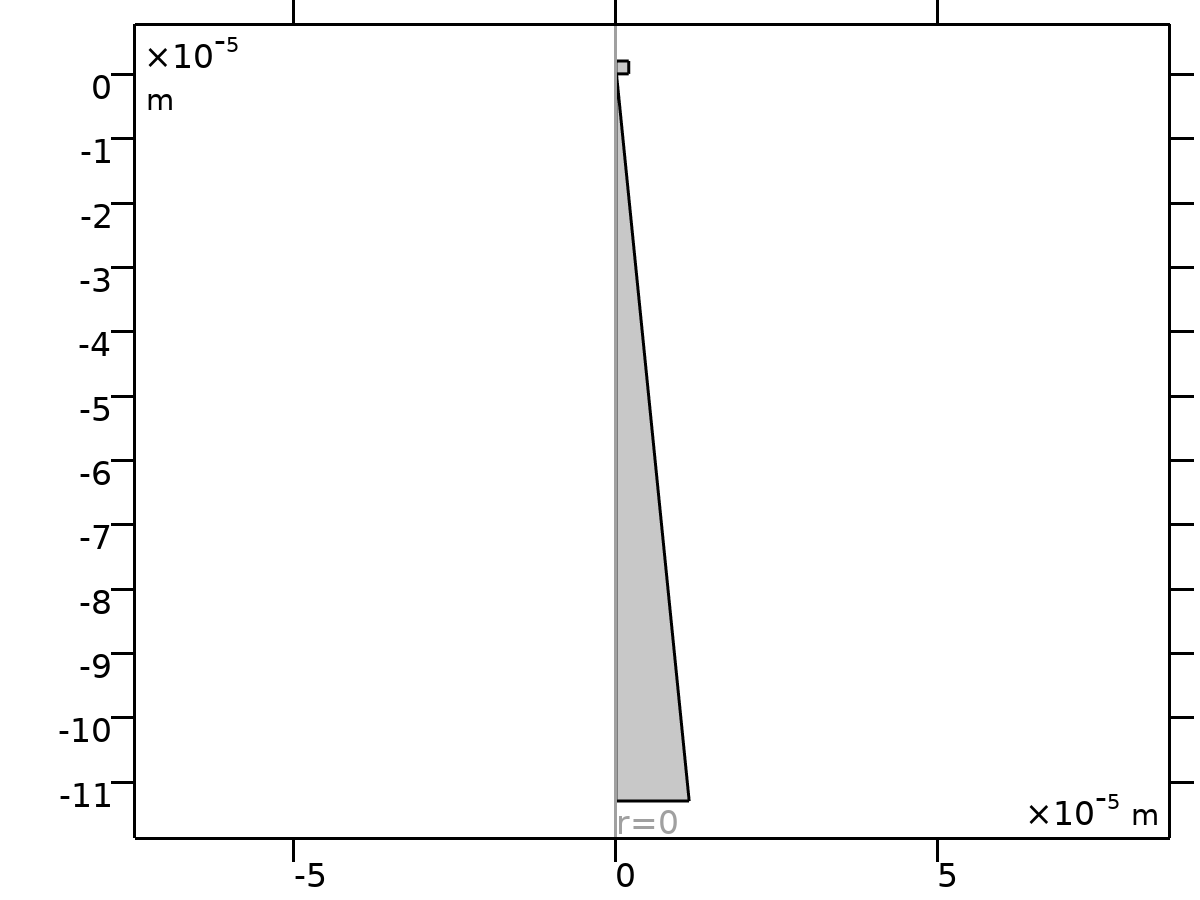


Mesh 1

Mesh statistics

| **Description** | **Value** |
| --- | --- |
| Minimum element quality | 0.0 |
| Average element quality | 0.0 |

- - 1. Size (size)

Settings

| **Description** | **Value** |
| --- | --- |
| Maximum element size | 4.26E-6 |
| Minimum element size | 1.44E-8 |
| Curvature factor | 0.25 |
| Maximum element growth rate | 1.25 |
| Predefined size | Finer |

- - 1. Size 1 (size1)

Selection

| Geometric entity level | Boundary |
| --- | --- |
| Selection | Geometry geom1: Dimension 1: Boundaries 4–5, 7 |


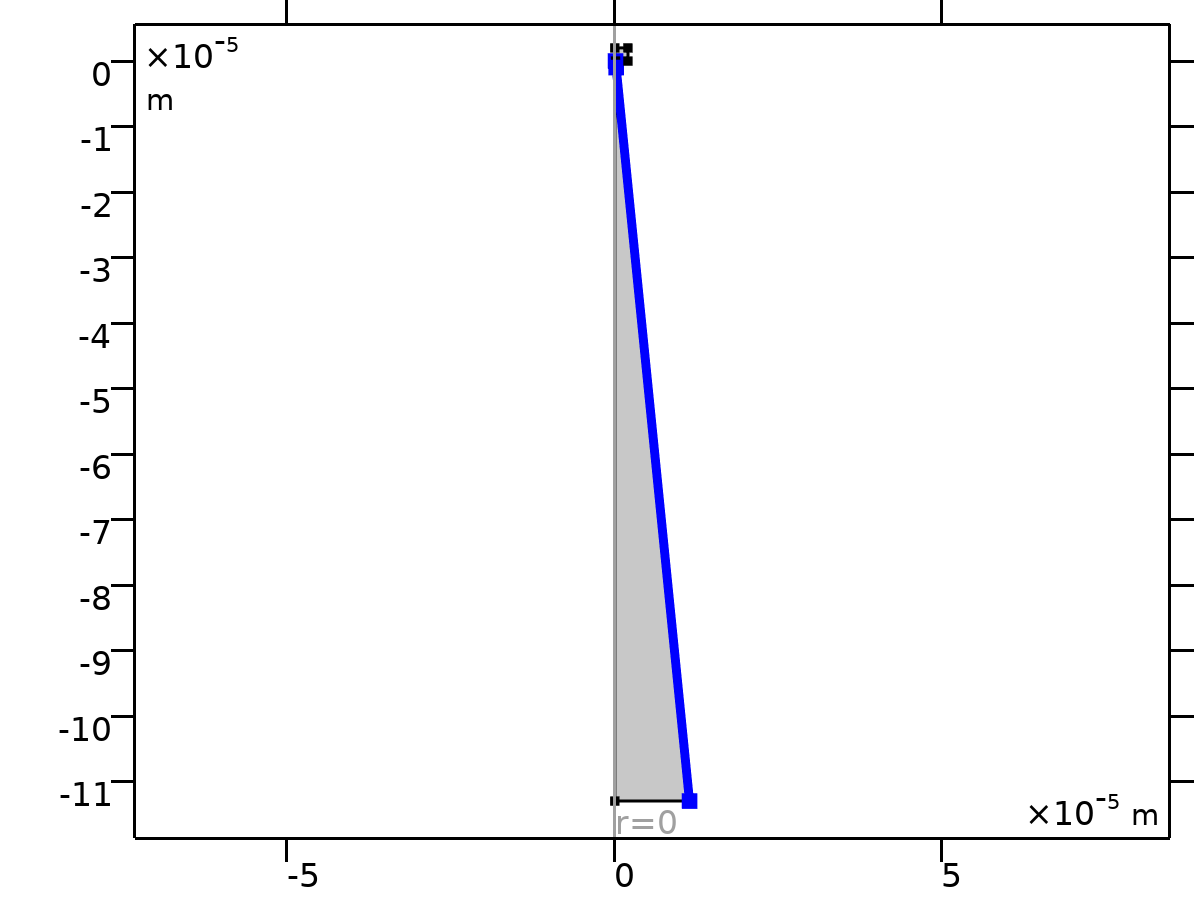


Size 1

Settings

| **Description** | **Value** |
| --- | --- |
| Maximum element size | 1e-8 |
| Minimum element size | 4.2E-9 |
| Minimum element size | Off |
| Curvature factor | 0.3 |
| Curvature factor | Off |
| Resolution of narrow regions | Off |
| Maximum element growth rate | 1.3 |
| Maximum element growth rate | Off |
| Custom element size | Custom |

- - 1. Size 2 (size2)

Selection

| Geometric entity level | Point |
| --- | --- |
| Selection | Geometry geom1: Dimension 0: Point 3 |


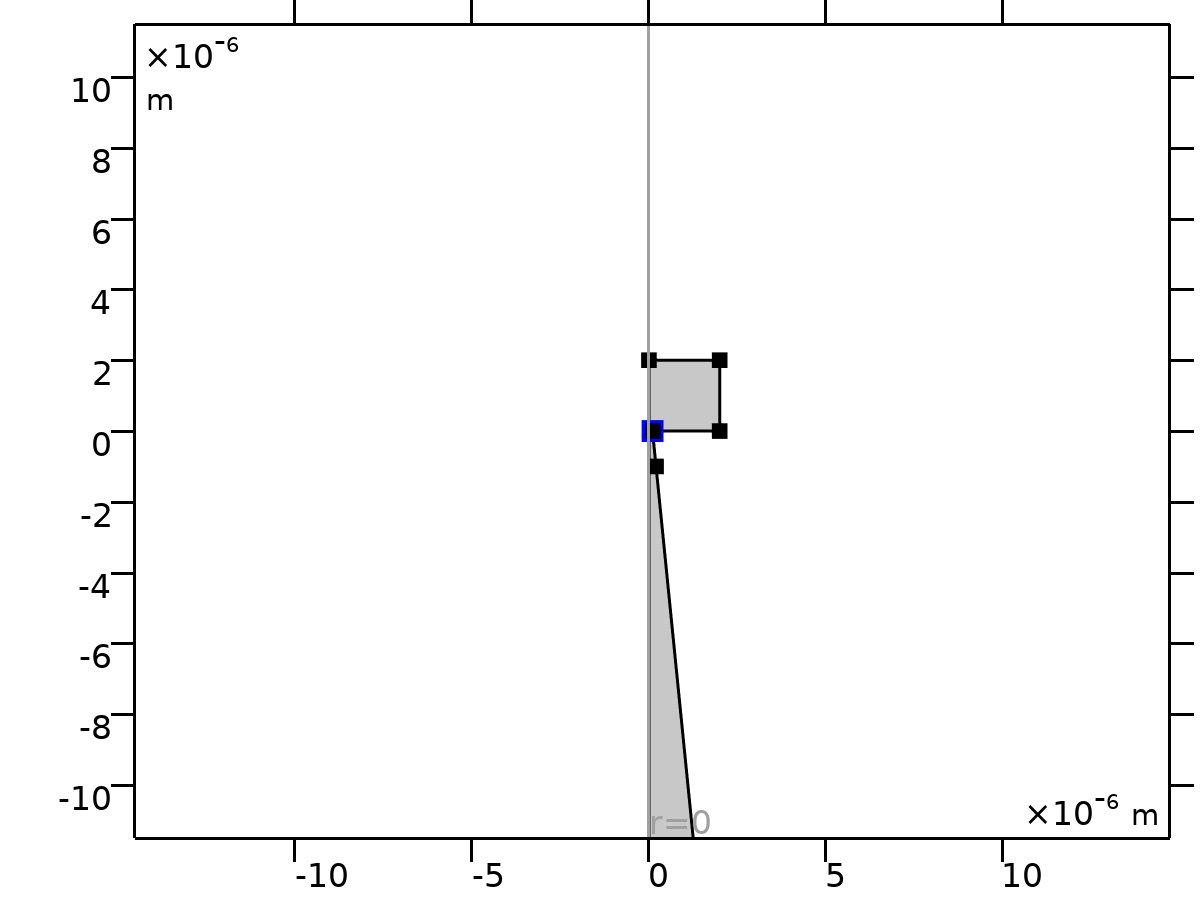


Size 2

Settings

| **Description** | **Value** |
| --- | --- |
| Maximum element size | 1e-10 |
| Minimum element size | 4.2E-9 |
| Minimum element size | Off |
| Curvature factor | 0.3 |
| Curvature factor | Off |
| Resolution of narrow regions | Off |
| Maximum element growth rate | 1.3 |
| Maximum element growth rate | Off |
| Custom element size | Custom |

- - 1. Size 3 (size3)

Selection

| Geometric entity level | Boundary |
| --- | --- |
| Selection | Geometry geom1: Dimension 1: Boundaries 2–3, 6 |


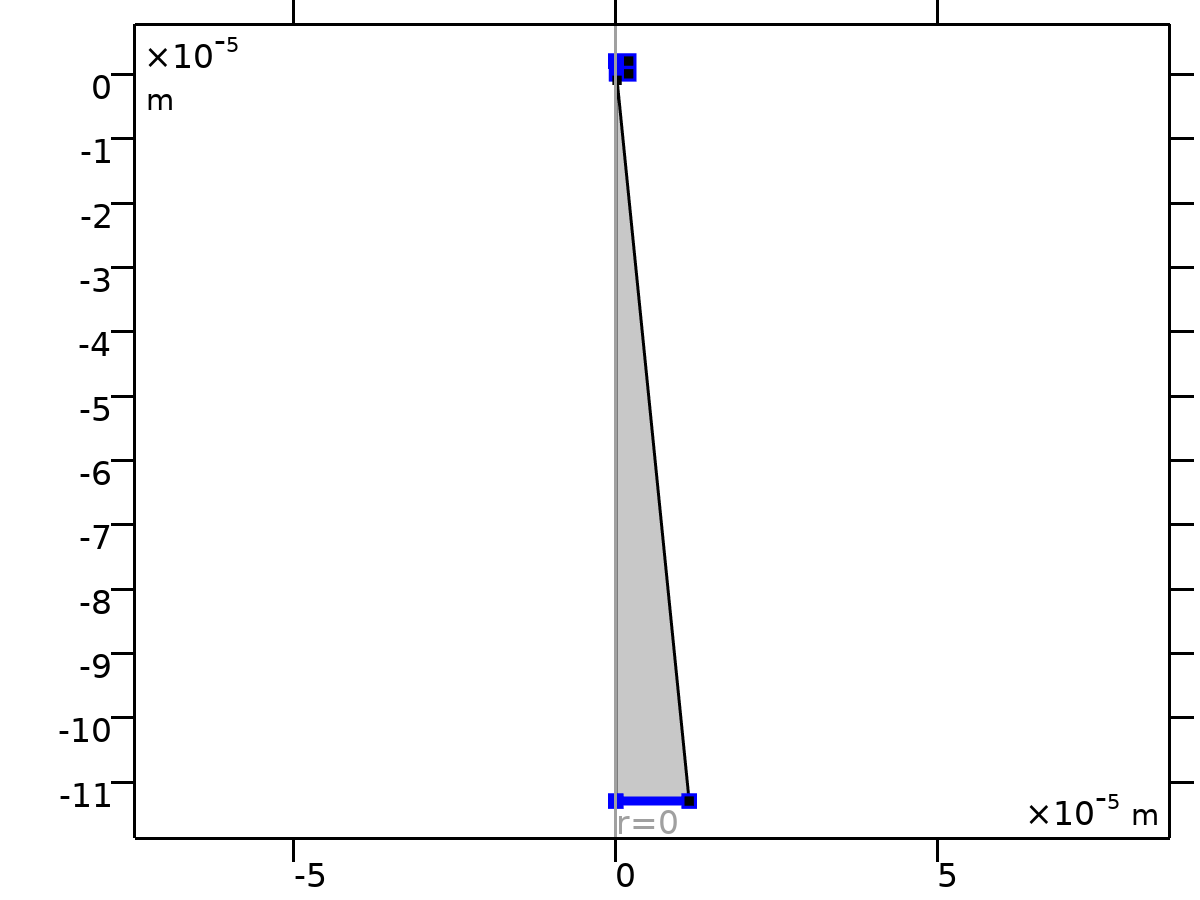


Size 3

Settings

| **Description** | **Value** |
| --- | --- |
| Maximum element size | 5E-8 |
| Minimum element size | 3.9E-9 |
| Minimum element size | Off |
| Curvature factor | 0.3 |
| Curvature factor | Off |
| Resolution of narrow regions | Off |
| Maximum element growth rate | 1.3 |
| Maximum element growth rate | Off |
| Custom element size | Custom |

- - 1. Free Triangular 3 (ftri3)

Selection

| Geometric entity level | Remaining |
| --- | --- |


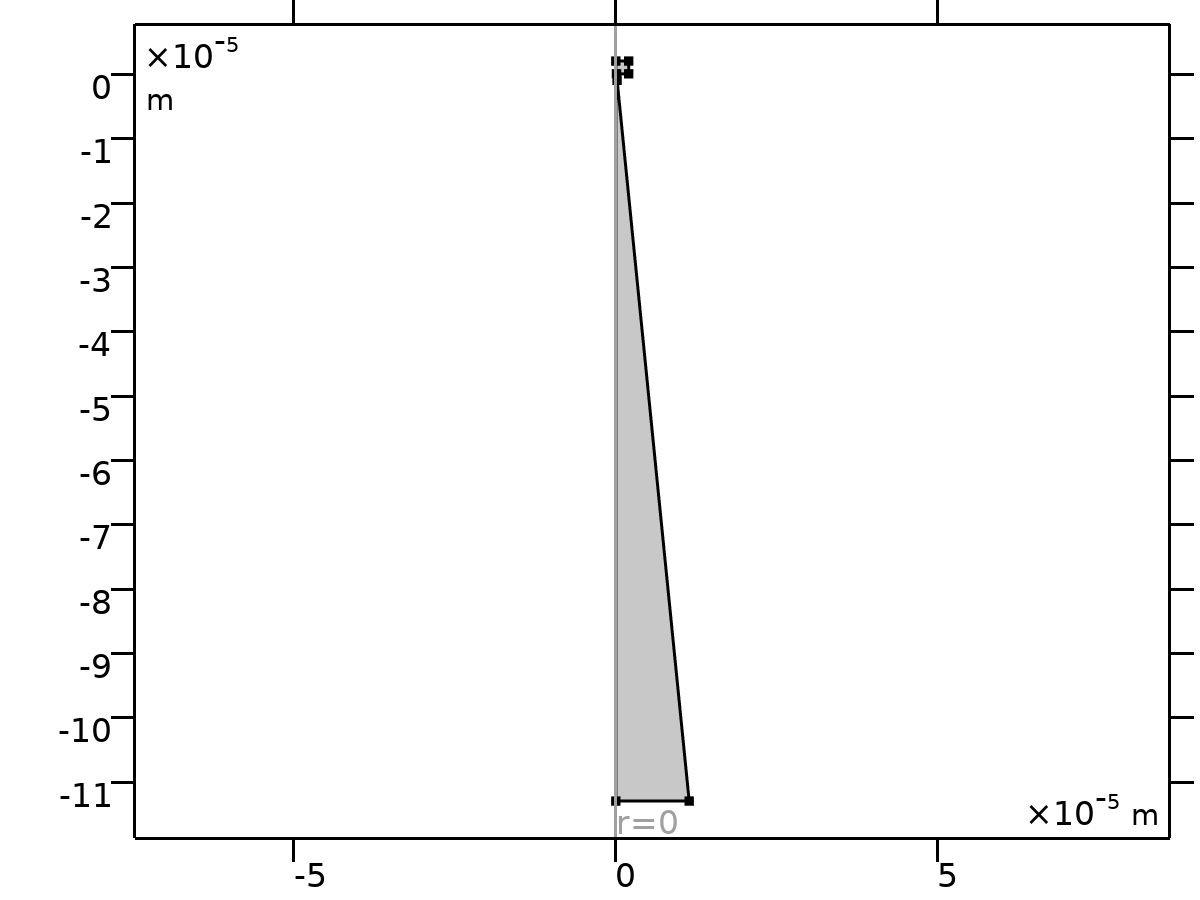


Free Triangular 3

1. Study 2

Computation information

| Computation time | 10 min 16 s |
| --- | --- |

- 1. Parametric Sweep

| **Parameter name** | **Parameter value list** | **Parameter unit** |
| --- | --- | --- |
| depth | 70,113 | um |

Study settings

| **Description** | **Value** |
| --- | --- |
| Sweep type | Specified combinations |
| Parameter name | depth |
| Unit | um |

Parameters

| **Parameter name** | **Parameter value list** | **Parameter unit** |
| --- | --- | --- |
| depth | 70,113 | um |

- 1. Time Dependent

| **Times** | **Unit** |
| --- | --- |
| range(0,8/399,8) | s |

Study settings

| **Description** | **Value** |
| --- | --- |
| Include geometric nonlinearity | Off |

1. Results
   1. Data Sets
      1. Study 2/Solution 1

Solution

| **Description** | **Value** |
| --- | --- |
| Solution |  |
| Component | Component 1 (comp1) |


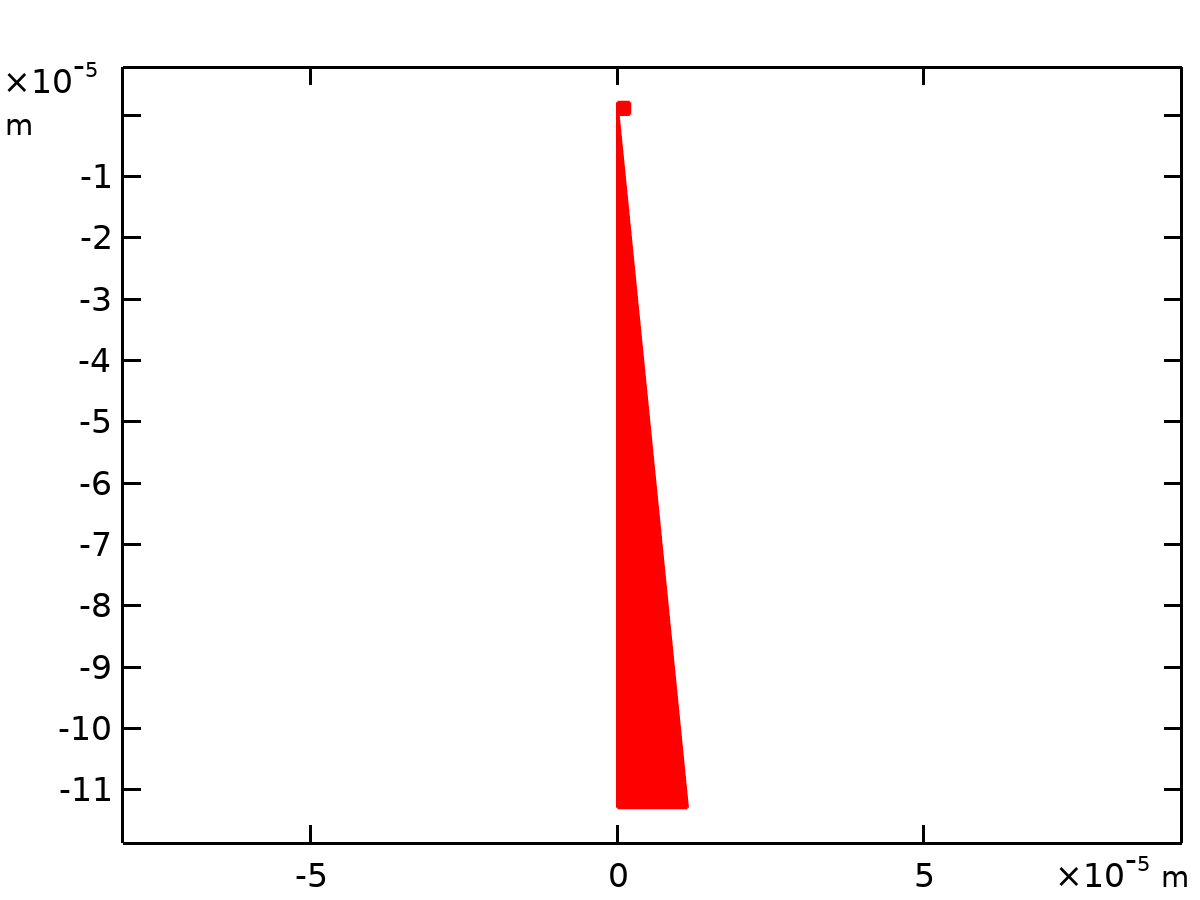


Dataset: Study 2/Solution 1

- - 1. Study 2/Parametric Solutions 1

Solution

| **Description** | **Value** |
| --- | --- |
| Solution |  |
| Component | Component 1 (comp1) |


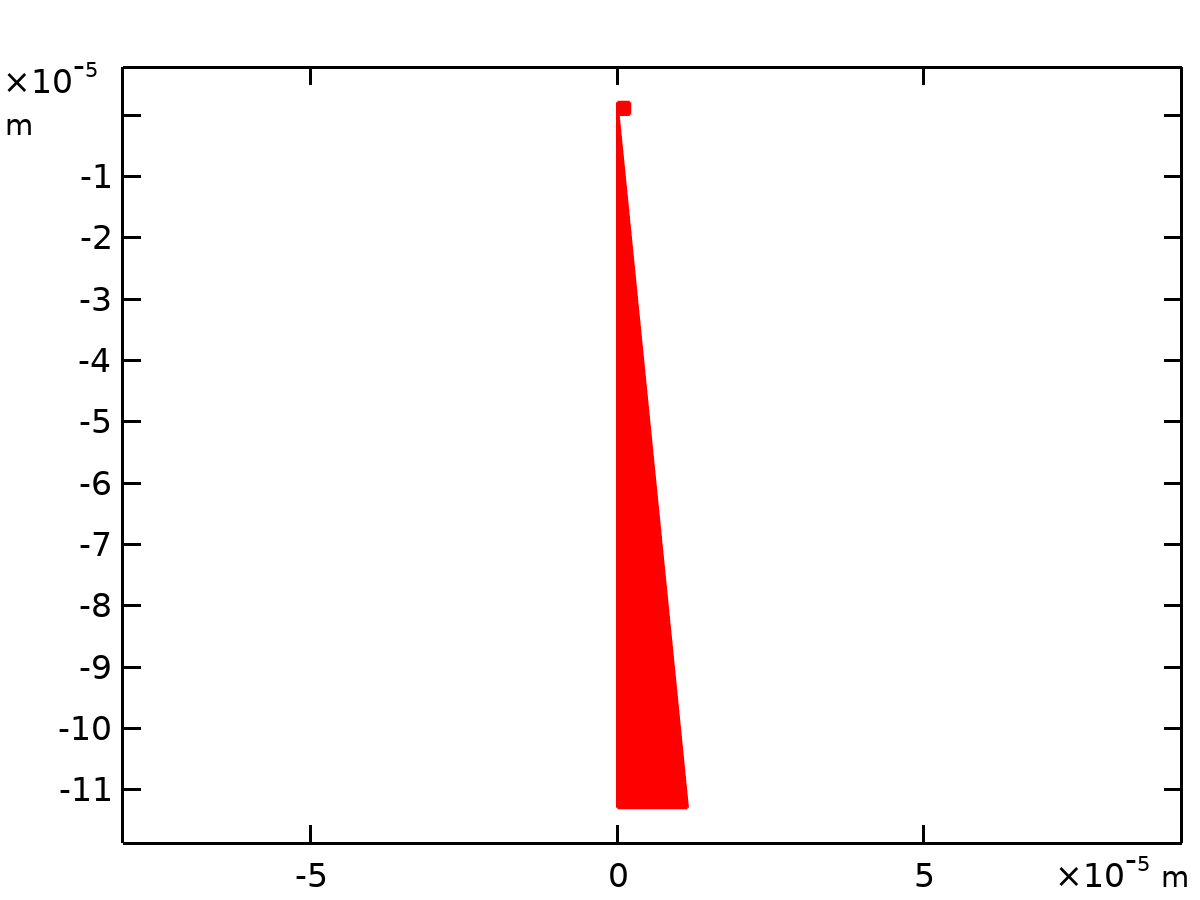


Dataset: Study 2/Parametric Solutions 1

- - 1. Revolution 2D 1

Data

| **Description** | **Value** |
| --- | --- |
| Dataset |  |

Axis data

| **Description** | **Value** |
| --- | --- |
| Axis entry method | Two points |
| Points | {{0, 0}, {0, 1}} |

Revolution layers

| **Description** | **Value** |
| --- | --- |
| Start angle | -90 |
| Revolution angle | 225 |


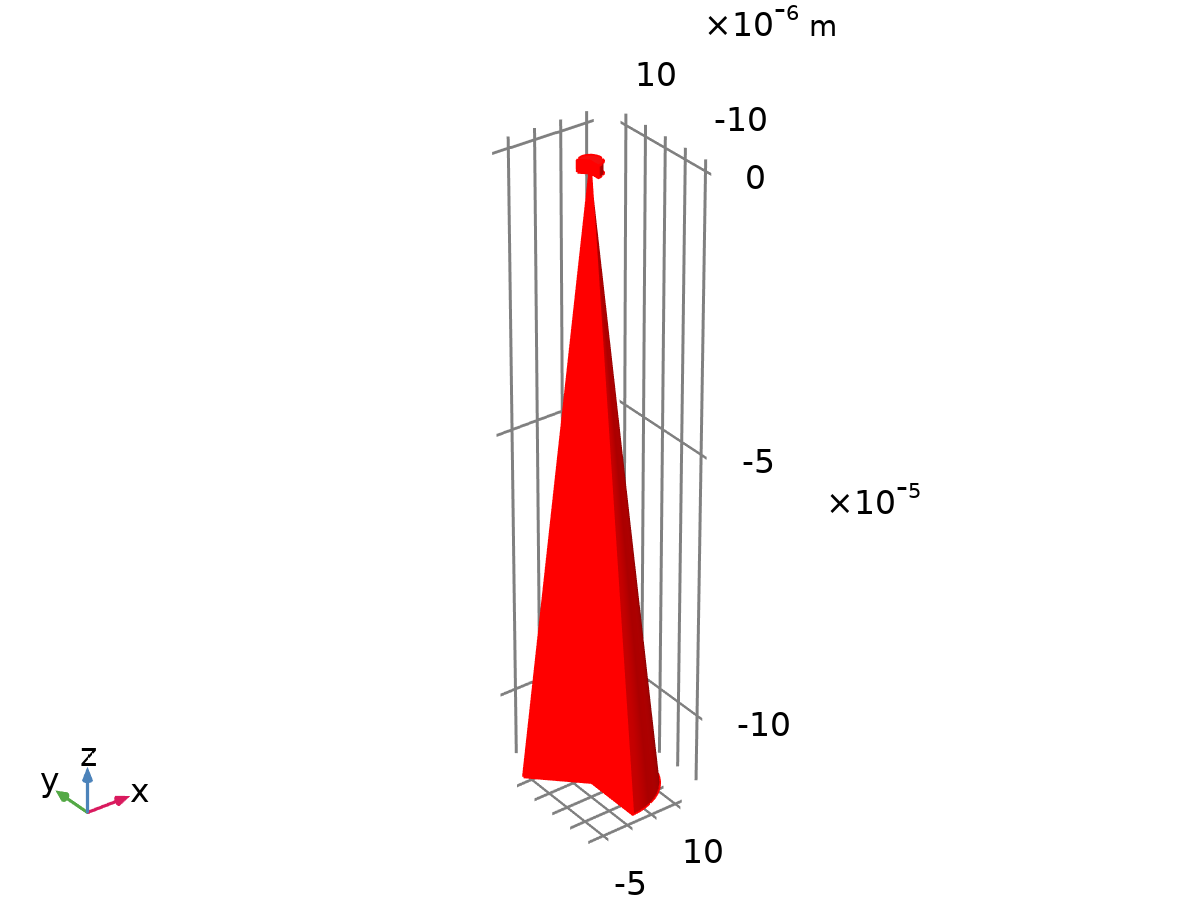


Dataset: Revolution 2D 1

- 1. Plot Groups
     1. Concentration, O (chds)


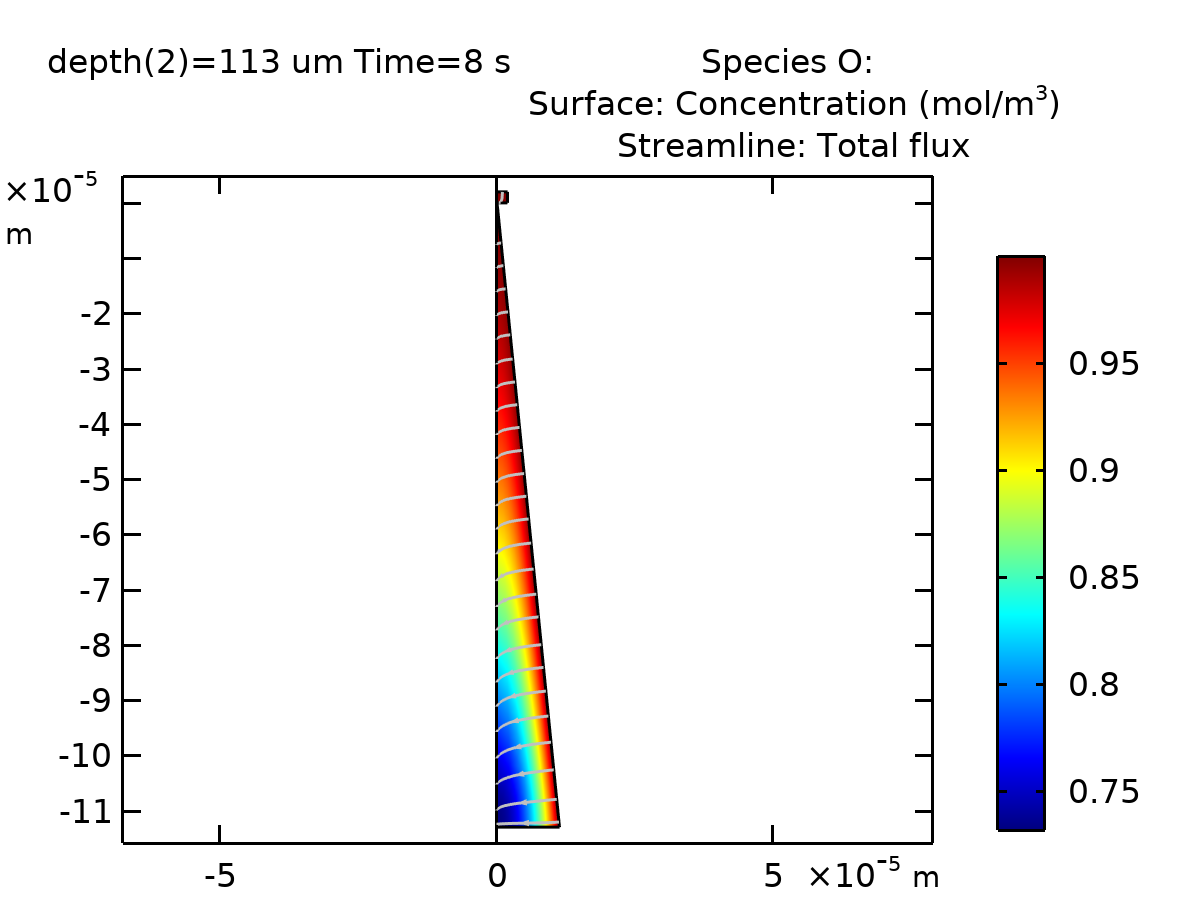


Species O: Surface: Concentration (mol/m^3^) Streamline: Total flux

- - 1. Concentration, O, 3D (chds)


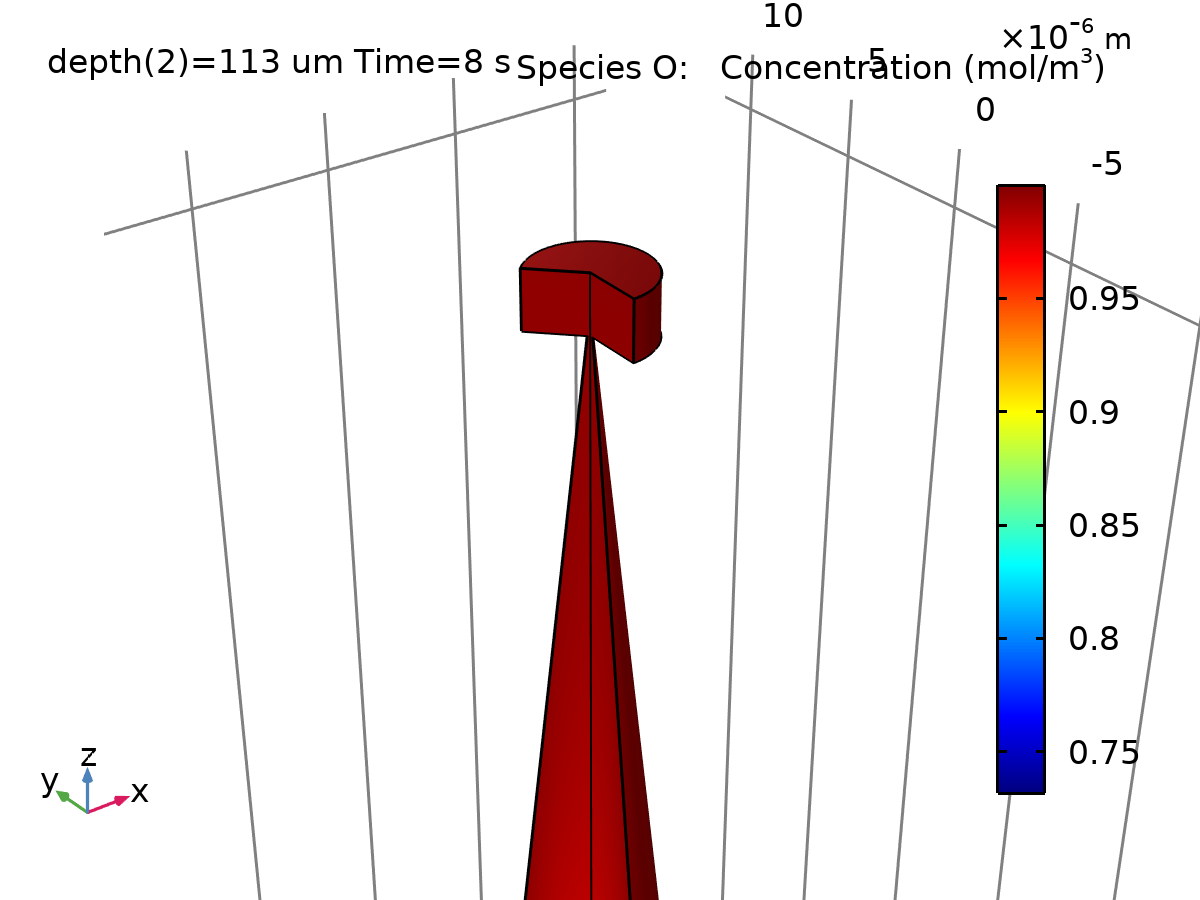


Species O: Concentration (mol/m^3^)

- - 1. Concentration, R (chds)


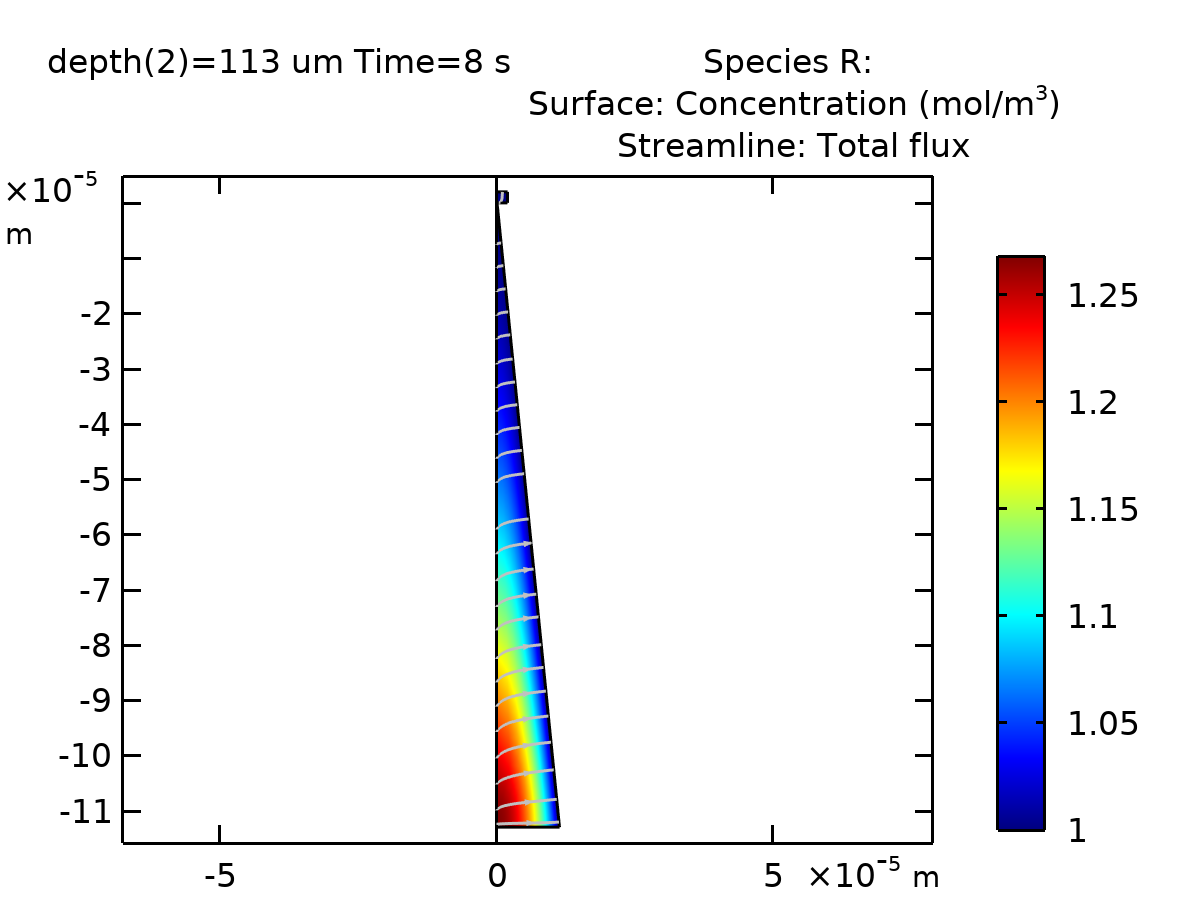


Species R: Surface: Concentration (mol/m^3^) Streamline: Total flux

- - 1. Concentration, R, 3D (chds)


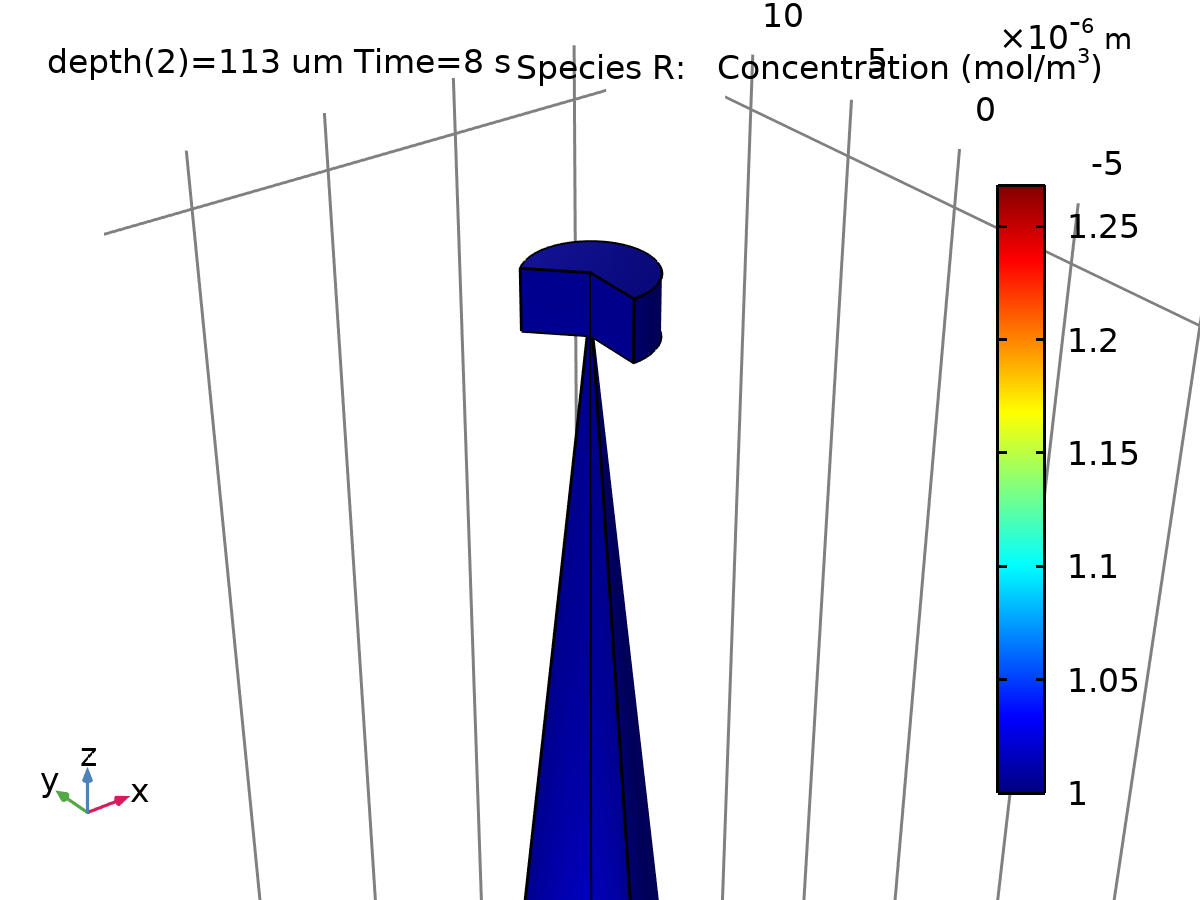


Species R: Concentration (mol/m^3^)
