# Supplementary material for: Interconversions between RNS Revealed by Transient Voltammetry with Porphyrin‐Modified Carbon Nanopipettes in Single Living Cells
Source: Adv Sci (Weinh). 2025 Oct 6;12(48):e12865. doi: 10.1002/advs.202512865 (PMC12752564; doi:10.1002/advs.202512865)
Supplement: Supplementary file 2 — Supporting Information [file ADVS-12-e12865-s002.docx]

Supporting Information

Interconversions Between RNS Revealed by Transient Voltammetry with Porphyrin-Modified Carbon Nanopipettes in Single Living Cells

Hongli Cao, Dehui Yu, Yingjie Zhao, Xiaoyue Shen, Rujia Liu*, Dengchao Wang*

**Table of Contents:**

1. **Experimental Section**

**Figure S1** TEM image of the CNP, and the chemical structure of TMPyP.

**Figure S2** The CVs of different modification times of CNP with 0.05 mM TMPyP in 1 mM K_4_[Fe(CN)_6_.

**Figure S3** The CVs of CNP at different NaNO_2_ concentrations.

**Figure S4** The CVs of TMPyP-CNPs in 1 mM NaNO_2_, (A) with different CNP modification times with 0.1 mM TMPyP, (B) and different TMPyP concentrations after 5 min.

**Figure S5** The CVs of first and 50th cycles for TMPyP-CNP in 1 mM NaNO_2_.

**Figure S6** The CVs of TMPyP-CNP in 1 mM NaNO_2_ and H_2_O_2_.

**Figure S7** (A) The CVs of CNP at different scan rate in 1 mM NaNO_2_. (B) The oxidation peak current at various *v* for the CNP.

**Evaluation of the time duration of the NO_2_ intermediate**

**Figure S8** Corresponding statistical studies of charge and concentration in eight single cells

**Figure S9** The CVs of TMPyP-CNP at different scan rates.

**Table S1** Comparison of current research and previously published studies on the detection of NO_2_⁻.

**Table S2** Calculated Gibbs free energies (ΔG) of elementary steps for NO_2_⁻ oxidation on the TMPyP.

**References**

1. **Experimental section**

**Chemical and Materials.** Potassium chloride (KCl), sodium chloride (NaCl), sodium sulfate (Na_2_SO_4_), sodium nitrite (NaNO_2_), ammonium chloride (NH_4_Cl), L-ascorbic acid (AA), uric acid (UA), dopamine hydrochloride (DA), phosphate buffered saline buffer (PBS buffer, pH 7.4) premixed powder, potassium ferrocyanide trihydrate ([K_4_Fe(CN)_6_·3H_2_O]), sodium nitroprusside (SNP) and hydrogen peroxide(H_2_O_2_) were obtained from Macklin. Norepinephrine hydrochloride (NA) and sodium carbonate (Na_2_CO_3_) were purchased from Aladdin, and Cytochrome *C* (CytC, BR) was from Shanghai yuanye Bio-Technology Co., Ltd. 5,10,15,20-tetra(4-pyridyl)-21H,23H-porphine was acquired from Shanghai Acmec Biochemical Technology Co., Ltd. DMEM basic (1X) and trypsin EDTA solution A (0.25% trypsin & 0.02% EDTA) were purchased from Gibco. Fetal Bovine Serum (Prime) was obtained from ExCell Bio. All other chemicals were used as received. All aqueous solutions were prepared using deionized water (18 MΩ·cm, total organic content < 10 ppb) equipped from a Milli-Q 3 UV system (Millipore).

**Instrumentation and Procedures.** The size and geometry of CNPs were characterized by transmission electron microscopy (TEM) with a JEOL TEM-2100 system. All electrochemical measurements in this work were conducted by a CHI760E potentiostat inside the custom-built Faraday cage. A two-electrode system was employed, with carbon nanopipettes (CNPs) serving as the working electrodes, and the external reference electrodes were homemade Ag/AgCl electrodes. The electrochemical measurements of the cells were performed using an inverted fluorescence microscope (Nikon Eclipse Ti2-U, Japan). The insertion and extraction of the electrode in the HeLa cell were precisely controlled by a motorized 3D micromanipulator (RWD-MM-500). For cyclic voltammetry tests, the sample interval is 0.001 V. For normal pulse voltammetry, the pulse width is 0.06 V, the pulse period is 0.2 s, sample width is 0.02 s and the step E is 0.01 V.

**Fabrication of carbon nanopipettes（CNPs）.** Quartz nanopipettes were prepared by pulling quartz capillaries (Q100-50-7.5, o.d. = 1 mm, i.d. = 0.5 mm and length = 7.5 cm; Sutter Instrument Co), using a laser pipette puller (P-2000, Sutter Instrument Co.). The nanopipettes were produced with the following parameters: HEAT1 = 650, FIL1 = 3, VEL1 = 30, DEL1 = 180, PUL1 = 50; HEAT2 = 655, FIL2 = 4, VEL2 = 45, DEL2 = 130, PUL2 = 110. The carbon nanopipettes (CNPs) were fabricated via chemical vapor deposition, as previously reported.^[1, 2]^ Briefly, pulled quartz nanopipettes were placed in a high-temperature furnace set at 955 °C, where a carbon layer was deposited on their inner surfaces by thermally decomposing methane (CH₄) under a controlled mixed atmosphere of methane and argon (5:3 ratio) for 25 minutes. The thickness of the carbon layer could be adjusted by varying the temperature, deposition time, or gas mixture ratio.

**Synthesis of TMPyP-CNPs.** 5,10,15,20-tetra(4-pyridyl)-21H,23H-porphine modified carbon nanopipettes (TMPyP-CNPs) were synthesized by immersing CNPs in TMPyP solution at room temperature. The TMPyP concentration and modification time were further optimized, and 0.05 mM TMPyP and 5 min modification time were found to lead to the best performance (Figure S4). The chemical structure of TMPyP is shown in Figure S1B.

**Cell Culture and activation.** The human cervical adenocarcinoma HeLa cells were cultured at 37 ℃ under a 5% CO_2_ atmosphere in Dulbecco’s modified Eagle’s medium (DMEM) containing 10% fetal bovine serum (FBS) and 1% penicillin/streptomycin. Then all cells cultured in the dish were digested using a 0.25% trypsin solution. Subsequently, several compounds were selected to modulate intracellular levels of reactive nitrogen species (RNS). First, HeLa cells were replaced with fresh medium, followed by the addition of 10 μM H_2_O_2_ or VC. The cells were incubated in a CO_2_ incubator for 3 and 5 hours to induce or eliminate intracellular reactive nitrogen species. Separately, cells were incubated in a culture medium with 10 μM and 100 μM cytochrome c oxidase for 1 hour to convert intracellular nitrite into nitric oxide (NO). In a different experiment, cells were incubated in a culture medium with 10 μM and 100 μM sodium nitroprusside (SNP) for 1 h to assess the production of NO. After the cell incubations, electrochemical tests were carried out.

**Single-Cell Measurements.** Experiments were performed at controlled room temperature on the stage of an inverted microscope placed in a Faraday cage. Before measurements, the culture medium was removed from a Petri dish containing HeLa cells. The dish was then rinsed three times and partially filled with 10 mM PBS. Then, cells were first identified by optical microscopy and the nanopipette tip positioned above the target cell. Using a micromanipulator, the nanopipette was advanced toward the cell at a controlled, slow descent rate while the microscope focus was continuously adjusted until the tip entered the cytoplasm. Successful insertion was confirmed by a brief, subtle morphological change observed by microscopy and by a concomitant change in the recorded current before versus after insertion. The release and accumulation of reactive nitrogen species (RNS) were monitored in real-time via cyclic voltammetry (CV) with the homemade Ag/AgCl as a reference/conducting electrode in the bulk solution.

**Statistical Analysis.** Cyclic voltammetry (CV) traces were pre-processed by numerical integration of the relevant redox peaks to obtain the integrated peak area; this value was then divided by the scan rate to yield the enclosed charge (Q). Data are presented as mean ± SEM and the sample size for each statistical comparison was n = 8. Statistical significance was assessed by one-sided one-way analysis of variance (ANOVA, *α* = 0.05). The reported significance symbols are: ****p* ≤ 0.001, ***p* ≤ 0.01, **p* ≤ 0.05, n.s.: not significant. All statistical analyses and graphs were produced using OriginPro 2024.

**Calculation details.** The finite-element simulations were also performed to help study the modification efficiency and reaction mechanism, by using a commercial software package COMSOL Multiphysics 5.4 with a 2D-axisymmetric model.

1. For surface coverage evaluation study in Figure 1E:

The mass transport process is described by the diffusion law:

$J_{i}=-D_{i}\nabla c_{i}$ (S1)

Where *J_i_* is the total flux in nanopipette, *D_i_*, and *c_i_* are the diffusion coefficient and concentration of species *i*. At the electrode/solution interface, the flux of the redox molecules follows the Butler-Volmer equation:

$J_{R}= k^{0}c_{O}e^{-\alpha f(E-E_{0})}-k^{0}c_{R}e^{(1-\alpha)f(E-E_{0})}$ (S2)

$J_{O}= {-k}^{0}c_{O}e^{-\alpha f(E-E_{0})}+k^{0}c_{R}e^{(1-\alpha)f(E-E_{0})}$ (S3)

where *k^0^* is the standard rate constant, *E* is the electrode potential, *E_0_* is formal potential, *α* is the transfer coefficient, and *f* = zF/RT. *F* is Faraday constant, *R* is gas constant, *T* is temperature. Then, the current can be derived from the integration of the redox molecules flux:

$i= \int{FJ}_{R}dS$ (S4)

2) For the simulated CV responses of CEC processes in carbon nanopipettes:

A ⇌ R (S5)

R + ne^-^ ⇌ O (S6)

O + Z ⇌ R +Y (S7)

The diffusion of all the species follow the Equation S1, and at the electrode surface, the flux of R and O also follows the Butler-Volmer equation (Equation S2). Two additional chemical reaction occur:

For the reaction between A and R, assuming the forward and backward reaction rate of *k_1_* and *k_2_*:

$J_{A}=-k_{1}c_{A}+k_{2}c_{R}$ (S8)

For the following reaction between O and Z with reaction rate of k_3_ and k_4_:

$J_{O}=J_{Z}=-k_{3}c_{O}c_{Z}+k_{4}c_{R}c_{Y}$ (S9)

$J_{Y}=k_{3}c_{O}c_{Z}-k_{4}c_{R}c_{Y}$ (S10)

$J_{R}=k_{3}c_{O}c_{Z}-k_{4}c_{R}c_{Y}+k_{1}c_{A}-k_{2}c_{R}$ (S11)

The current can be obtained by integrating the total flux of the redox molecules at the electrode surface. More details can be seen from the COMSOL report.

**DFT calculation details:** In Density Functional Theory (DFT) calculations, we conducted structural optimizations using the generalized gradient approximation (GGA) with the Perdew-Burke-Ernzerhof (PBE) ^[3]^ functional. This work was carried out using the Vienna Ab-initio Simulation Package (VASP).^[4, 5]^ To address the interactions between ion cores and valence electrons, we employed the projector augmented-wave (PAW) method^[6, 7]^. The plane-wave cutoff energy was set to 450 eV. To account for Van der Waals interactions, we utilized Grimme's DFT-D3 method^[8, 9]^. The self-consistent calculations applied a convergence energy threshold of 10^-5^ eV, and the equilibrium geometries and lattice constants were optimized with a maximum stress of 0.02 eV Å^-1^ for each atom. During the relaxation process, we employed a 1 × 1 × 1 Gamma-centered grid for the Brillouin zone. Additionally, a 15 Å vacuum layer was included to prevent artificial interactions between periodic images. Spin-polarized calculations were also performed in this study.

The adsorption energy (Eads) is calculated as: Eads = E(total) - E(slab) - E(adsorbate). E(total) is the total energy of an optimized slab with the adsorbate on it, E(slab) is the energy of a relaxed clean slab, and E(adsorbate) is the energy of an adsorbate molecule.

For each elementary step, the Gibbs free energy change, ΔG, is defined as the difference between the free energies of the initial and final states. This is expressed by the following formula:

$\Delta G=\Delta E+\Delta E_{ZPE}\text{ – T}\Delta S.$ (S12)

where ΔE is the reaction energy of reactant and product molecules adsorbed on catalyst surface, obtained from DFT calculations; $\Delta E_{ZPE}$ and $\Delta S$ are the change in zero-point energies and entropy due to the reaction.


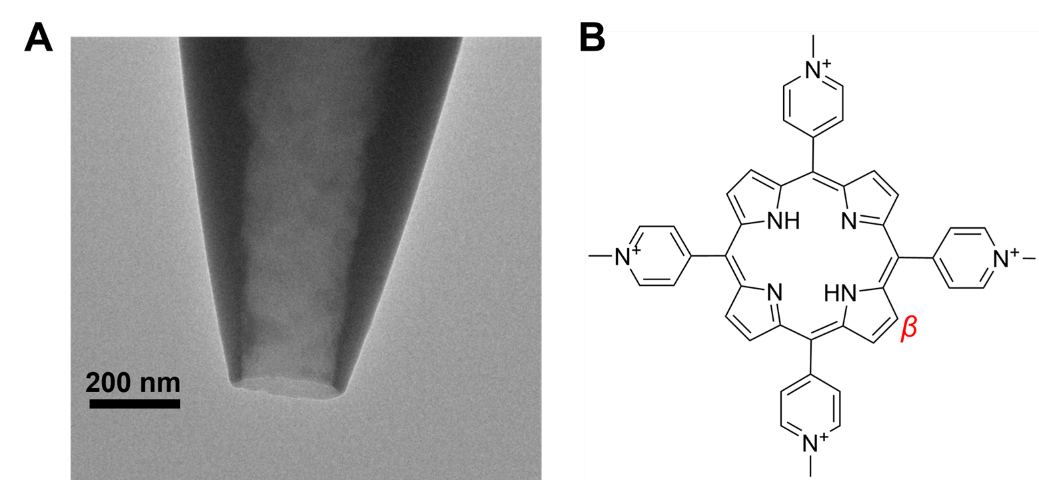


**Figure S1**. (A) TEM image of the CNP. (B) Chemical structure of TMPyP.


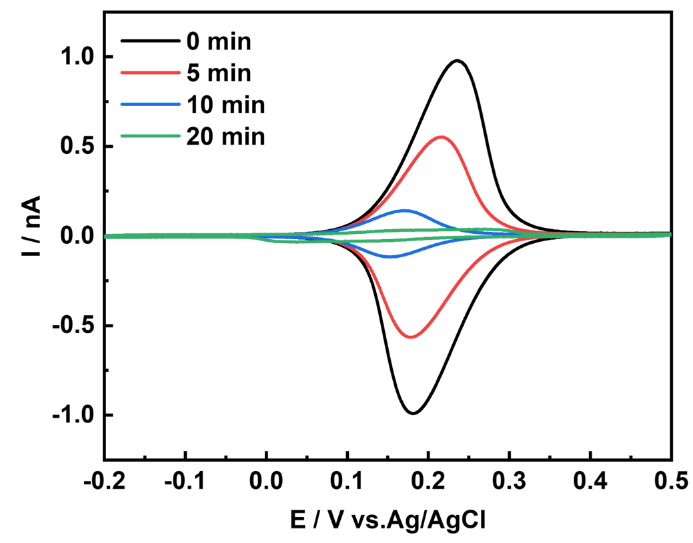


**Figure S2.** The CVs of a CNP at different modification times with 0.05 mM TMPyP. The solution is 1 mM K_4_Fe(CN)_6_ and 0.1 M KCl, and scan rate = 0.1 V/s.





**Figure S3**. The CVs of CNP at different NaNO_2_ concentrations in 10 mM PBS at a scan rate of 0.1 V/s


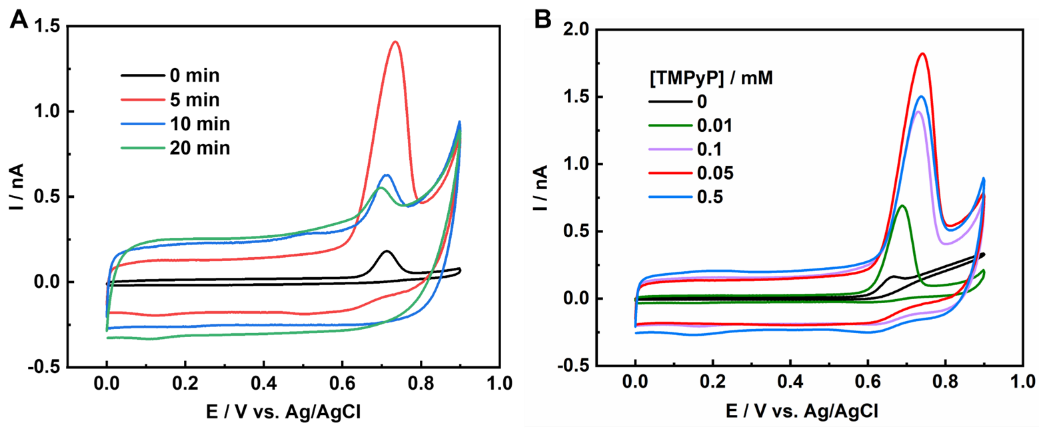


**Figure S4**. The CVs of TMPyP-CNPs in 1 mM NaNO_2_ and 10 mM PBS at a scan rate of 0.1 V/s, (A) different modification times with 0.1 mM TMPyP. (B) and different TMPyP concentrations after 5 min modification.


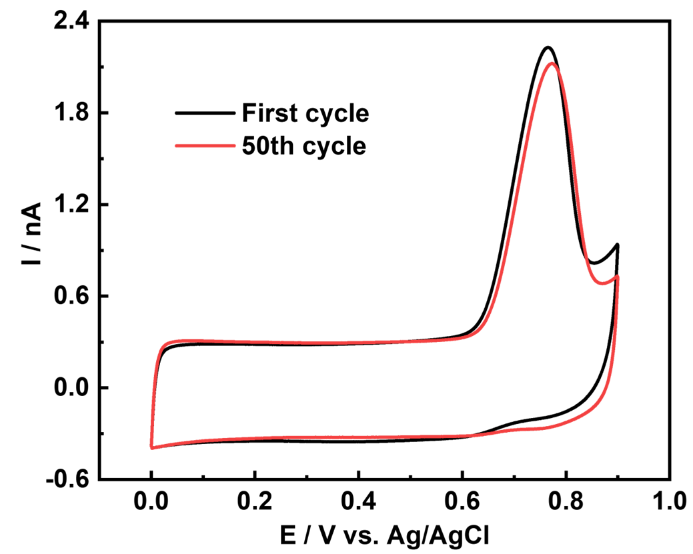


**Figure S5**. The CVs of first and 50th cycles for TMPyP-CNP in 1 mM NaNO_2_ and 10 mM PBS at a scan rate of 0.1 V/s.


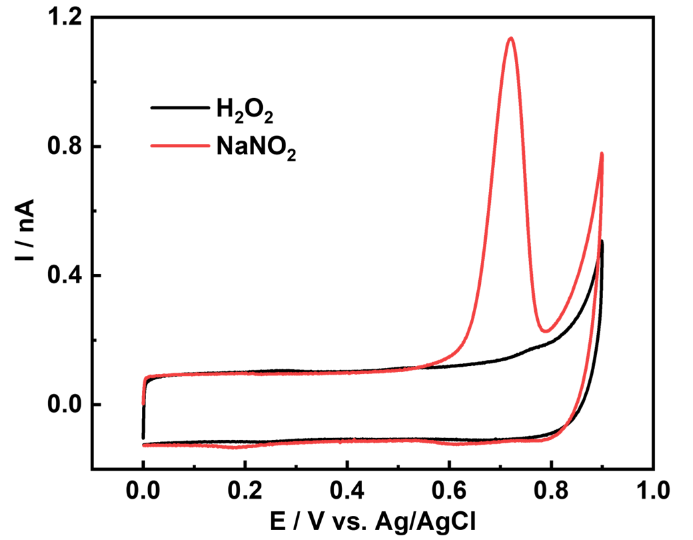


**Figure S6**. The CVs of TMPyP-CNP in 1 mM NaNO_2_ and H_2_O_2_ in 10 mM PBS at a scan rate of 0.1 V/s.


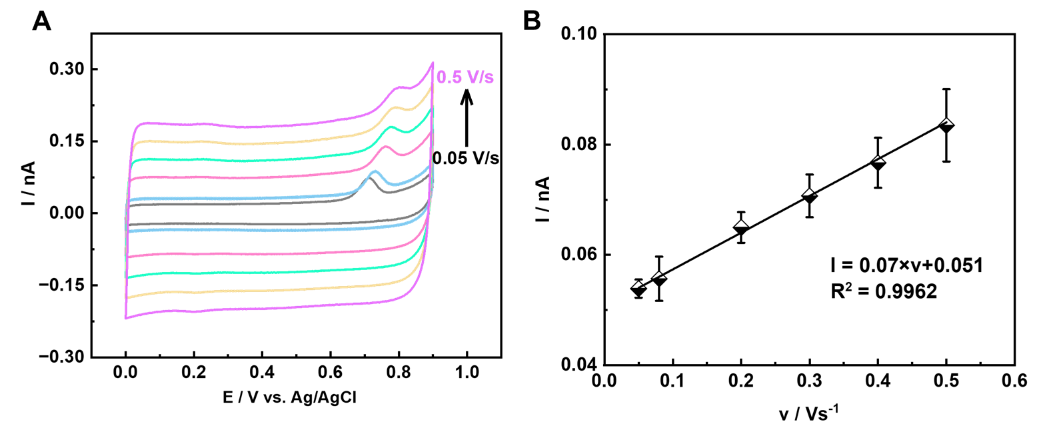


**Figure S7**. (A) CVs of the CNP at different *v*: 0.05, 0.08, 0.2, 0.3, 0.4, and 0.5 V/s, in 1 mM NaNO_2_ and 10 mM PBS. (B) The oxidation peak current at various *v* for CNP.

**Evaluation of the time duration of the NO_2_ intermediate:**

The lifetime of the intermediate NO_2_ can be estimated from the transient reduction peak in the CVs. At 0.15 V/s, we start to see reduction peak around +0.7 V. As the NO_2_⁻ get oxidized into NO_2_ at +0.7 V, when the potential scan from +0.7 to +0.9, and back to +0.7 V, the overall time duration is about (0.4 V) / (0.15 V/s) = 2.67 s.

The reported lifetime of the NO_2_ in the solution can also be estimated. For a reported^[10]^ rate constant of 6.5×10^7^ M^-1^ s^-1^ for the NO_2_ disproportion reaction with water, assuming an initial NO_2_ concentration of 1 mM, the lifetime is about 1/ (6.5×10^7^ M^-1^ s^-1^ × 10^-3^ M) = 0.015 μs.


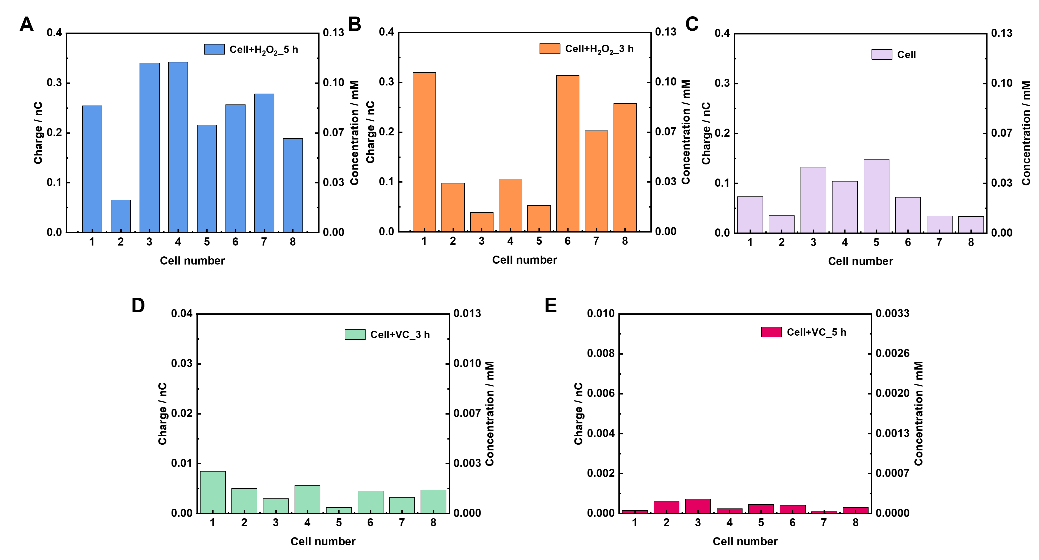


**Figure S8**. Corresponding statistical enclosed charges in the oxidation peak and corresponding NO_2_⁻ concentration in eight single cells treated by (A) 10 μM H_2_O_2_ for 5 h, (B) 10 μM H_2_O_2_ for 3 h, (C) Blank control. (D) 10 μM VC for 3 h, (E) 10 μM VC for 5 h.


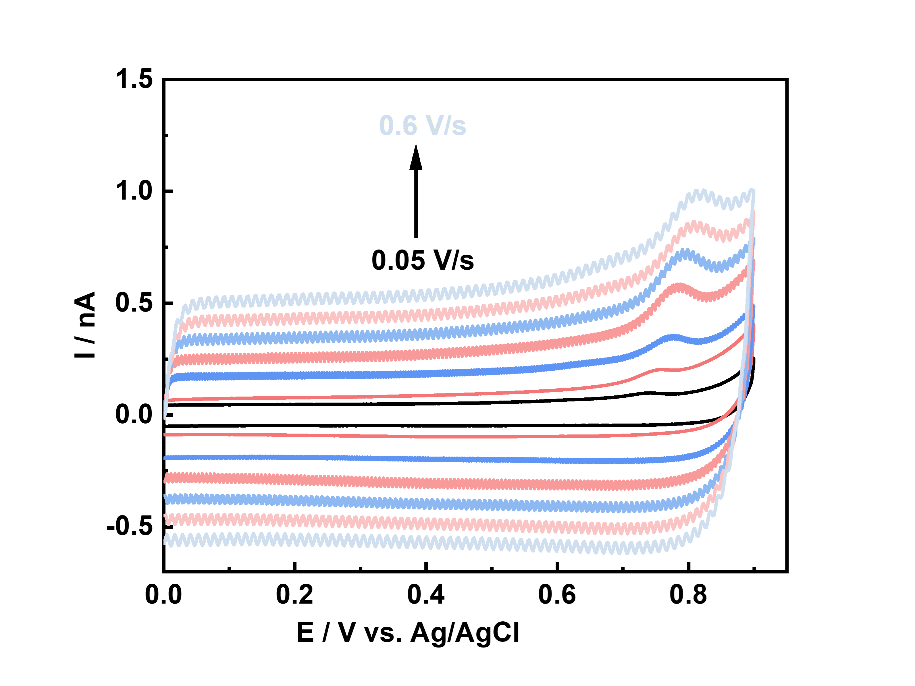


**Figure S9**. The CVs of the TMPyP-CNP in HeLa cell at different scan rates.

**Table S1.** Comparison of current research and previously published studies on the electrochemical detection of NO_2_⁻.

| Electrode | Mothod | Detection range (μmol/L) | LOD(μmol/L) | reference |
| --- | --- | --- | --- | --- |
| G4-NH_4_/MWNT-modified GCE | I-T | 5-1500 | 2.0 | ^[11]^ |
| Cyt c/l-Cys/P3MT/MWCNT/GCE | I-T | 10-100 | 0.5 | ^[12]^ |
| PdNPs-poly(1,5-DNA)/MWCNT | I-T | 0.25-100 | 0.08 | ^[13]^ |
| Porphyrin/MOF-525 | I-T | 20-800 | 2.1 | ^[14]^ |
| FeT_4_MPyP/CuTSPc/GCE | DPV | 0.5-7.5 | 0.14 | ^[15]^ |
| CTAB-GO/MWCNT | DPV | 5-800 | 1.5 | ^[16]^ |
| CNT/PPy nanocomposite | CV | 0.5-2000 | 0.5±0.025 | ^[17]^ |
| TMPyP-CNPs | CV | 1-1000 | 0.2 | This work |

**Table S2.** Calculated Gibbs free energies (ΔG) of elementary steps for NO_2_⁻ oxidation on the TMPyP.

| Elementary reactions | | ­△G / eV |
| --- | --- | --- |
|  |  | TMPyP |
| 1. (2) | * + NO_2_⁻ - e^-^ ⇋ [*(NO_2_)] | -0.7692 |
| (3) | 2[*(NO_2_)] + H_2_O ⇋ 2H^+^ + [*(NO_2_⁻)] + [*NO_3_⁻] | -2.9859 |

**References**

1. Y. Yu, J.-M. Noël, M. V. Mirkin, Y. Gao, O. Mashtalir, G. Friedman, Y. Gogotsi, Carbon pipette-based electrochemical nanosampler. *Anal. Chem.* **2014**, 86, 3365.
2. B. Kim, T. Murray, H. Bau, The fabrication of integrated carbon pipes with sub-micron diameters. *Nanotech.* **2005**, 16, 1317.
3. J. P. Perdew, K. Burke, M. Ernzerhof, Generalized gradient approximation made simple. *Phys. Rev. Lett.* **1996**, 77, 3865.
4. W. Kohn, L. J. Sham, Self-consistent equations including exchange and correlation effects. *Phys. Rev.* **1965**, 140, A1133.
5. P. Raybaud, J. Hafner, G. Kresse, S. Kasztelan, H. Toulhoat, Ab initio study of the H_2_–H_2_S/MoS_2_ gas–solid interface: The Nature of the Catalytically Active Sites. *J. Catal.* **2000**, 189, 129.
6. P. E. Blöchl, Projector augmented-wave method. *Phys. Rev. B* **1994**, 50, 17953.
7. G. Kresse, D. Joubert, From ultrasoft pseudopotentials to the projector augmented-wave method. *Phys. Rev. B* **1999**, 59, 1758.
8. S. Grimme, S. Ehrlich, L. Goerigk, Effect of the damping function in dispersion corrected density functional theory. *J. Comput. Chem.* **2011**, 32, 1456.
9. S. Grimme, J. Antony, S. Ehrlich, H. Krieg, A consistent and accurate ab initio parametrization of density functional dispersion correction (DFT-D) for the 94 elements H-Pu. *J. Chem. Phys.* **2010**, 132, 154104-1.
10. S. Goldstein, G. Czapski, J. Lind, G. Merenyi, Mechanism of decomposition of peroxynitric ion (O_2_NOO^‒^): evidence for the formation of O_2_**^·^**^‒^ and·NO_2_ radicals. *Inorg. Chem.* **1998**, 37, 3943.
11. N. Zhu, Q. Xu, S. Li, H. Gao, Electrochemical determination of nitrite based on poly (amidoamine) dendrimer-modified carbon nanotubes for nitrite oxidation. *Electrochem. Commun.* **2009**, 11, 2308.
12. M. Eguilaz, L. Agüí, P. Yanez-Sedeno, J. Pingarrón, A biosensor based on cytochrome c immobilization on a poly-3-methylthiophene/multi-walled carbon nanotubes hybrid-modified electrode. Application to the electrochemical determination of nitrite. *J. Electroanal. Chem.* **2010**, 644, 30.
13. S. Shi, Z. Li, Y. Chen, J. Yang, H. Xu, J. Huang, Y. Zhang, C. Zou, J. Qiao, Electrochemically co-deposition of palladium nanoparticles and poly (1, 5-diaminonaphthalene) onto multiwalled carbon nanotubes (MWCNTs) modified electrode and its application for amperometric determination of nitrite. *Int. J. Electrochem. Sci.* **2019**, 14, 7983.
14. C.-W. Kung, T.-H. Chang, L.-Y. Chou, J. T. Hupp, O. K. Farha, K.-C. Ho, Porphyrin-based metal-organic framework thin films for electrochemical nitrite detection. *Electrochem. Commun.* **2015**, 58, 51.
15. W. J. Santos, P. R. Lima, A. A. Tanaka, S. M. Tanaka, L. T. Kubota, Determination of nitrite in food samples by anodic voltammetry using a modified electrode. *Food Chem* **2009**, 113, 1206.
16. Y. J. Yang, W. Li, CTAB functionalized graphene oxide/multiwalled carbon nanotube composite modified electrode for the simultaneous determination of ascorbic acid, dopamine, uric acid and nitrite. *Biosens. Bioelectron.* **2014**, 56, 300.
17. S. Rajesh, A. K. Kanugula, K. Bhargava, G. Ilavazhagan, S. Kotamraju, C. Karunakaran, Simultaneous electrochemical determination of superoxide anion radical and nitrite using Cu, ZnSOD immobilized on carbon nanotube in polypyrrole matrix. *Biosens. Bioelectron.* **2010**, 26, 689.
